# Functionalizing DNA Origami by Triplex-Directed Site-Specific Photo-Cross-Linking

**DOI:** 10.1021/jacs.4c03413

**Published:** 2024-05-02

**Authors:** Shantam Kalra, Amber Donnelly, Nishtha Singh, Daniel Matthews, Rafael Del Villar-Guerra, Victoria Bemmer, Cyril Dominguez, Natalie Allcock, Dmitry Cherny, Andrey Revyakin, David A. Rusling

**Affiliations:** † Department of Molecular and Cell Biology, and Leicester Institute of Chemical Biology, University of Leicester, Leicester LE1 7RH, U.K.; ‡ Centre for Enzyme Innovation, School of Biological Sciences, University of Portsmouth, Portsmouth, Hampshire PO1 2DY, U.K.; § Core Biotechnology Services Electron Microscopy Facility, University of Leicester, Leicester LE1 7RH, U.K.; ∥ School of Medicine, Pharmacy and Biomedical Sciences, University of Portsmouth, Portsmouth PO1 2DT, U.K.

## Abstract

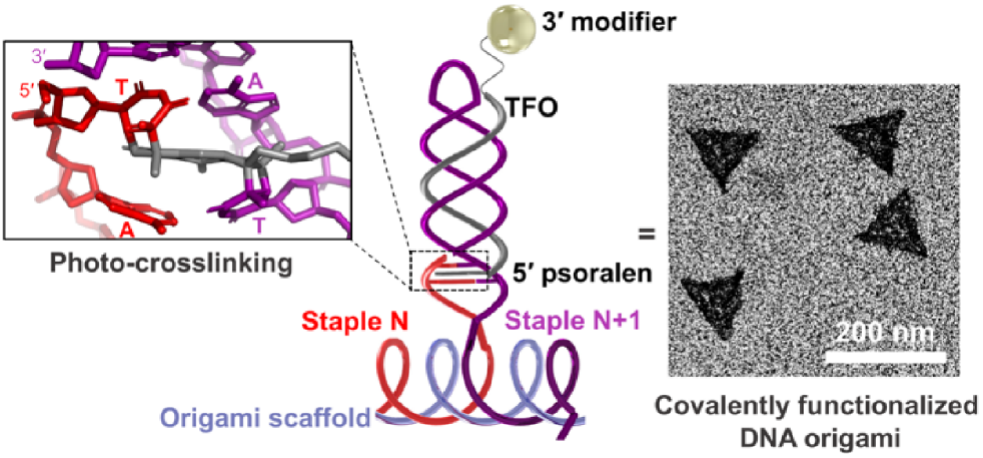

Here, we present a cross-linking approach to covalently functionalize and stabilize DNA origami structures in a one-pot reaction. Our strategy involves adding nucleotide sequences to adjacent staple strands, so that, upon assembly of the origami structure, the extensions form short hairpin duplexes targetable by psoralen-labeled triplex-forming oligonucleotides bearing other functional groups (pso-TFOs). Subsequent irradiation with UVA light generates psoralen adducts with one or both hairpin staples leading to site-specific attachment of the pso-TFO (and attached group) to the origami with ca. 80% efficiency. Bis-adduct formation between strands in proximal hairpins further tethers the TFO to the structure and generates “superstaples” that improve the structural integrity of the functionalized complex. We show that directing cross-linking to regions outside of the origami core dramatically reduces sensitivity of the structures to thermal denaturation and disassembly by T7 RNA polymerase. We also show that the underlying duplex regions of the origami core are digested by DNase I and thus remain accessible to read-out by DNA-binding proteins. Our strategy is scalable and cost-effective, as it works with existing DNA origami structures, does not require scaffold redesign, and can be achieved with just one psoralen-modified oligonucleotide.

## Introduction

Nucleic acid (NA) nanotechnology is a bottom-up nanofabrication approach that harnesses the self-assembly properties and well-understood structure of nucleic acids to create molecular objects or patterns ranging from a few nanometers to a few microns in characteristic size.^1^ DNA origami is a subfield of NA nanotechnology in which a long single-stranded DNA “scaffold” strand is woven in two or three dimensions using a few hundred shorter DNA “staple” strands.^2,3^ DNA origamis are straightforward to design,^4^ relatively cost-effective to produce, and offer potential applications in a wide range of disciplines.^5^ Examples of applications of DNA origami in biomedicine include vaccines,^6^ enzymatic cascades,^7^ biological nanosensors,^8^ drug delivery,^9^ super-resolution microscopy,^10^ structural biology,^11^ basic single-molecule research,^12^ and, perhaps most aptly, delivery vehicles for genetic materials.^13^

Although origamis have been functionalized with numerous types of organic and inorganic moieties, methods that allow their site-specific attachment to the structure are still not trivial.^5^ Such guest molecules are frequently conjugated to oligonucleotides that are introduced before or during the assembly of the nanostructure. However, some molecules cannot tolerate the relatively harsh conditions required for origami folding, *e.g.,* if the guest molecule is a protein, high annealing temperatures can lead to its denaturation. Moreover, the attachment of more than one guest molecule to the structure requires its conjugation to individual staples which can substantially increase cost. A different approach relies on the recruitment of conjugated oligonucleotides *via* Watson–Crick (W–C) hybridization to single-stranded staple overhangs projected from the assembled nanostructure. However, such noncovalent interactions can result in stochastic dissociation of the oligonucleotides and loss of the introduced component. Other post-assembly modification strategies exist, such as the recruitment of cargo to specific sequences or reactive groups introduced into the structure but can result in a low yield of the functionalized complex.^14−18^

Once assembled, functionalized origami structures suffer from several other limitations that have restricted their widespread use.^19^ First, origami structures are not covalently sealed; rather, they are held together by weak (W–C) hydrogen bonding between the single-stranded DNA scaffold and hundreds of staples. This makes the nanostructures sensitive to thermal denaturation,^20^ chaotropic agents,^21^ depletion of metal ions,^22^ changes in pH,^23^ and other treatments. Second, single- and double-stranded regions of origami are susceptible to degradation or disassembly by DNA processing enzymes found in biological media and live cells (*e.g.,* nucleases,^24^ RNA polymerase,^25^*etc.*). This can lead to the loss of guest molecules held in place by the relatively short staple oligonucleotides assembled within or projected from the structure. Various methods have been developed to improve the stability of duplex regions within DNA origami, such as through the enzymatic^26^ or chemical ligation^27,28^ of nick sites located between adjacent staple strands, or the “welding” of strands within or projected from the origami by high-energy ultraviolet light (310 nm, UVB).^20 ,29,30^ While this can lead to dramatic stabilization, these approaches are not always cost-effective and might compromise the application of introduced functionalities (*e.g.,* by photobleaching of incorporated fluorophores)^31^ or alter the sequence and structural integrity of the origami core (*e.g.,* introducing DNA damage impassable for RNA polymerases and other enzymes).^13,20,29,30^ In addition, as far as we are aware, these covalent stabilization strategies have yet to be coupled to the attachment of guest molecules to the nanostructures.

**Figure 1:**
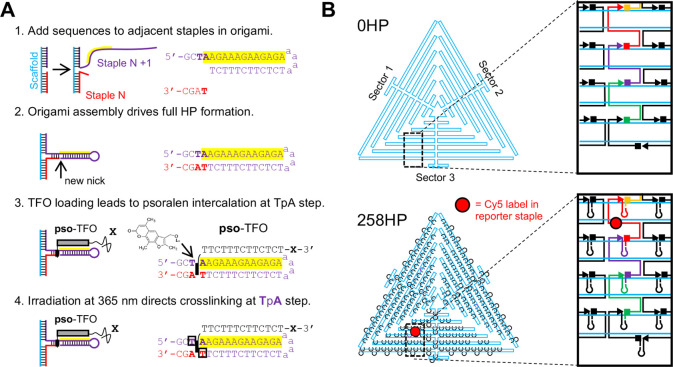
Targeting, cross-linking, and functionalization of DNA origami by psoralen-modified TFOs. (A) targetable hairpins are introduced into origami by the attachment of nucleotide sequences to the 5′ and 3′ ends of any two adjacent staples (*i.e.*, staple *N* and *N*+1). The extension of staple *N*+1 (purple) encodes for a stem-loop hairpin and contains the TFO-binding sequence (shown in yellow) and a 4-nt linker that contains the TpA sequence destined for psoralen cross-linking (in bold). Staple *N* (red) encodes the 4-nt compliment to the linker so that, upon origami assembly, a cross-linkable duplex between adjacent hairpins is formed. Targeting by a pso-TFO leads to psoralen intercalation across the TpA step (shown as black bar). Irradiation with UVA (365 nm) light triggers mono- and bis-adduct formation between psoralen and the thymidine bases in the TpA step (shown by the boxes), covalently attaching the TFO to the origami. Bis-adduct formation between staples in proximal hairpins further tethers the TFO and leads to formation of “super-staples” that enhance the structural integrity of the complex. Functionalization is achieved using pso-TFOs that carry additional moieties (X) at their 3′ end. (B) Design of the prototypical DNA origami triangle used in this study. Scaffold routing for 0HP and 258HP origami is shown in blue, and the 258 hairpins in 258HP are shown as black semicircles. Routing of the staples ensured that staple–staple junctions and TFO-binding hairpins alternated between opposite faces of the triangle to allow the functionalization of origami on both sides. The folding, cross-linking, and degradation of these complexes was monitored using a Cy5-labeled reporter staple 1 (shown in red). Adjacent to staple 1 are reporter staples 0, 2, and 3 (orange, purple, and green, respectively), which are used in later experiments and to illustrate how TFO-binding hairpins are formed by adjacent staples.

Here, we present a strategy based on triplex-directed photo-cross-linking that can be used to introduce functionality and improve the structural integrity of DNA origami in a single step (Figure 1). Triplex-forming oligonucleotides (TFOs) are sequence-specific DNA recognition agents^32,33^ that bind within the major groove of polypurine–polypyrimidine duplex sequences.^34^ TFOs have been used as a means to attach functional groups to DNA by the conjugation of non-nucleic acid components to the 5′ or 3′ ends of the oligonucleotide (*e.g.*, fluorescent dyes, reactive groups, proteins, *etc*.).^34,35^ The site-specific incorporation of the attached functional group is achieved by targeting the TFO to appropriate polypurine–polypyrimidine sites embedded within the DNA sequence.^36−38^ TFOs have also been used to direct site-specific cross-linking reactions within DNA by attaching the photo-cross-linking agent 4,5,8-trimethylpsoralen (psoralen) to the 5′ end of the oligonucleotide.^39−41^ TFO binding directs psoralen intercalation at a TpA step flanking the 5′-end of the triplex–duplex junction. Subsequent irradiation at 365 nm leads to a 2 + 2 cycloaddition reaction between the psoralen and opposing thymidine residues, cross-linking the two duplex strands and the oligonucleotide to the DNA (Figure S1). Free psoralen has already been shown to dramatically enhance origami stability.^20^ Our strategy harnesses both the functionalization and cross-linking properties of pso-TFOs to site-specifically modify DNA origami. Moreover, through appropriate design, we show that this approach can be used to reduce the sensitivity of the functionalized origami to thermal denaturation and to disassembly by T7 RNA polymerase. We also show through susceptibility to DNase I digestion that our directed cross-linking approach does not lead to damage of the underlying origami core. Our strategy is cost-effective, as it works with existing DNA origami structures, does not require scaffold redesign, and can be achieved with a single psoralen-modified oligonucleotide. The cross-linking reaction is fast (seconds) and is therefore scalable, energy-efficient, and leads to only minor photobleaching of fluorescent functionalities introduced into the structure.

## Results

### Design of TFO-Targetable Hairpin-Modified Origami

Due to sequence binding constraints and a requirement for a TpA step for cross-linking, it is not possible to use pso-TFOs to target origami folded from commercially available scaffolds directly, *e.g.,* M13mp18 or its derivatives.^42^ Our strategy therefore involved introducing a cross-linkable TFO target duplex sequence into the stem-loop hairpin(s) (HP) projected from the origami staples. This was achieved by extending the 3′ end of a specific origami staple(s) with a 32-nt sequence that folds intramolecularly into a 12-nt HP separated from the scaffold *via* a 4-nt single-stranded linker sequence (shown in purple in Figure 1A). The HP encodes an appropriate TFO-binding sequence, and the single-stranded linker encodes the “top” strand of the TpA duplex sequence destined for psoralen cross-linking. It also required extending the 5′ end of the adjacent staple(s) with a single-stranded 4-nt sequence, which encodes the complementary “bottom” strand of the TpA sequence (shown in red in Figure 1A). Upon origami assembly, the 3′ linker sequence from one staple (Staple *N*+1) is positioned next to the 5′ linker complement from the adjacent staple (Staple *N*), generating a TpA duplex that can be cross-linked between thymidine residues positioned by the neighboring staple strands. Cross-linking of multiple adjacent HPs was expected to form “super-staples” that further tether the TFO to the structure and increase the structural integrity of the origami. Functionalization is made possible by the attachment of cargo to the free end of the pso-TFO (shown as X in Figure 1A).^40^ Here, we used CT-motif TFOs that bind parallel to their target duplex and exhibit their highest affinity at low pH (pH < 6.0). We used this pH dependence to separate noncovalent binding from covalent attachment to the HP staples. An alternative would be to use AG-motif TFOs that bind antiparallel to their target duplex and are not pH sensitive. However, such oligonucleotides are prone to secondary structure formation, including GA-duplexes and G-quadruplexes.

**Figure 2:**
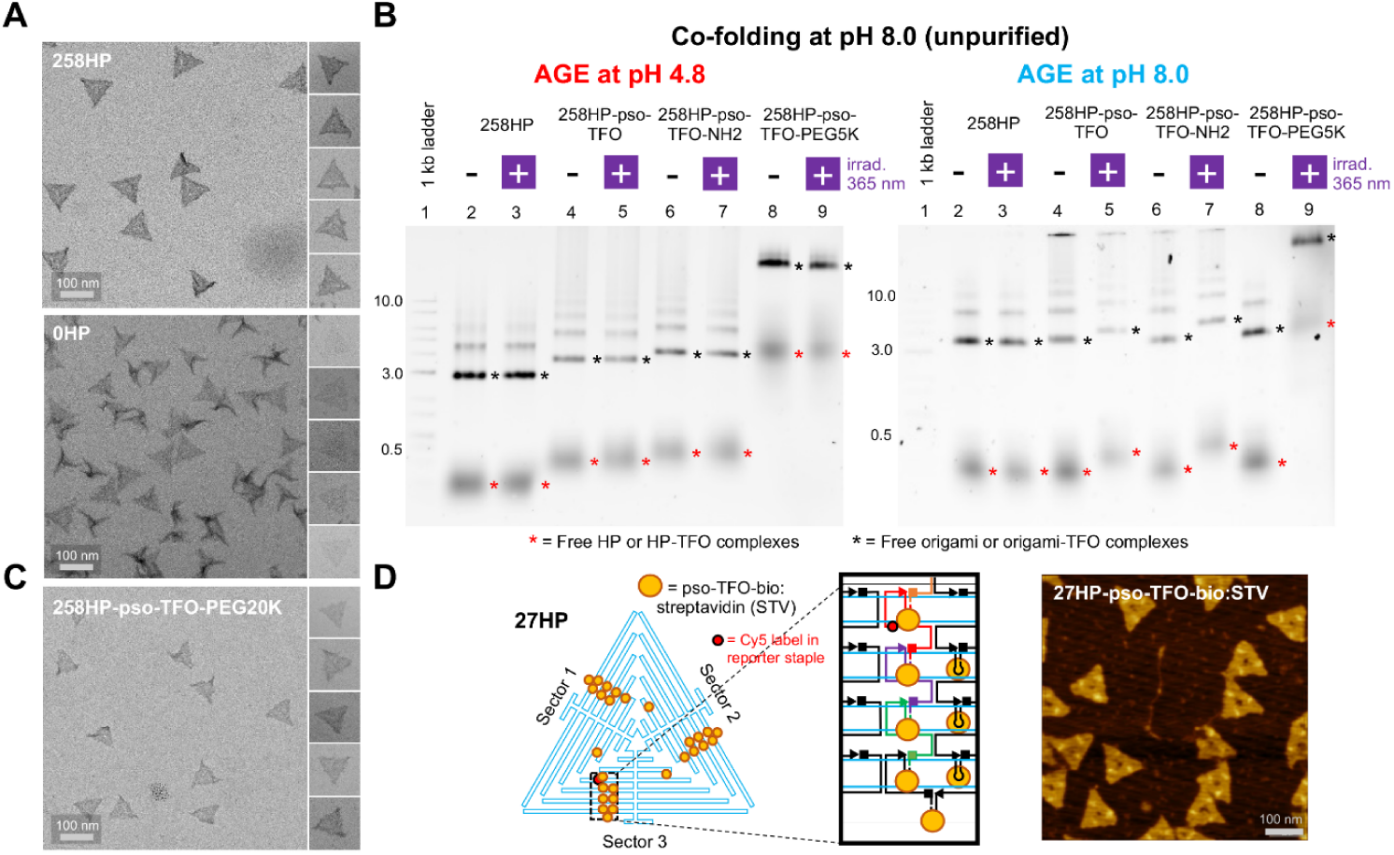
Targeting of origami hairpins by TFOs bearing additional moieties. (A) Representative TEM images of purified 258HP (top image) and 0HP (bottom image) origami triangles. (B) AGE analysis of the cofolded mixture of 258HP origami with pso-TFOs bearing different 3′-modifications. Origami structures were prepared in pH 8.0 TAE-Mg buffer by annealing a 50 nM scaffold with 150 nM staples in the absence or presence of 100 μM pso-TFOs. Samples were irradiated and analyzed by AGE in either pH 4.8 (left gel) or pH 8.0 (right gel) running buffers. Positions of the expected HP and HP-TFO complexes are shown using a red asterisk, while the positions of the origami and origami-TFO complexes are shown using a black asterisk. The bands located above the origami band in each lane are due to misfolded aggregates (expected during origami folding)^43^ and are removed by subsequent purification. Gels were scanned for EtBr fluorescence. (C) Representative TEM images of purified 258HP origami loaded with pso-TFO-PEG20K after psoralen cross-linking. (D) Site-specific targeting of 27HP origami structures with pso-TFO-PEG_4_-biotin. Left: scaffold routing for 27HP origami, which contains 27 TFO-binding hairpins in three clusters. TFO-bound streptavidin is shown as orange circles. Right: Representative AFM images of cross-linked and purified 27HP-pso-TFO-PEG_4_-biotin origami after incubation with 100 μM streptavidin for 30 min.

To test our strategy, we designed^4^ a prototypical origami triangle^3^ held together by 261 staples and comprised of three independently folding sectors (Figure 1B). We extended both the 3′- and 5′-ends of 258 staple strands with the sequences described above and positioned HPs on either side of the origami. The ends of the three remaining staples were left unmodified as place holders for future experiments on origami functionalization. We folded the 258HP nanostructure (258HP) using a 8064-nt M13 single-stranded DNA scaffold^42^ and a 3-fold molar excess of HP-staples (Figure 1B). As a control, we also folded HP-free triangles (0HP) that were identical in staple routing but contained no HPs. Agarose gel electrophoresis (AGE) of purified 0HP and 258HP origami revealed bands consistent with the successful formation of both types of nanostructures (Figure S2). As expected, the mobility of 258HP was reduced compared to 0HP, consistent with the extra molecular mass of 258 HPs (equivalent to the mobility at ∼3.0 *versus* ∼2.5 kb of linear dsDNA, respectively). Subsequent imaging of purified^42^ nanostructures by transmission electron microscopy (TEM) revealed that the 258HP origami formed homogeneous particles of the desired size and shape (Figures 2A top and S3). In contrast, imaging of the 0HP nanostructures revealed that these HP-free triangles often appeared “scrunched” on the TEM grid surface, indicating that, like similar structures, they might be more flexible in solution (Figures 2A bottom and S3).^43,44^ Taken together, these results show that the addition of HPs at the staple–staple junctions is compatible with origami folding and might also rigidify this type of nanostructure.

### HP-Origami Are Targetable by Unmodified and Modified pso-TFOs

We first established conditions for loading and cross-linking of pso-TFOs with an individual oligonucleotide containing just the hairpin sequence. The HP oligonucleotide was cofolded with different concentrations of the pso-TFO in the same pH 8.0 buffer as used for 0HP- and 258HP origami folding above. Although psoralen-modified CT-containing TFOs have been shown to cross-link DNA at neutral pH,^45^ it was not known if they would bind their targets at pH 8.0. Aliquots of the folded HP-pso-TFO complexes were subjected to UV irradiation at 365 nm using a home-built light-emitting diode (LED) setup (Figure S4). It was expected that this would induce formation of either mono- or bis-adducts between the pso-TFO and the full TpA step located in the HP oligonucleotide. To assay the formation of cross-linked and non-cross-linked complexes, the samples were separated by nondenaturing polyacrylamide gel electrophoresis (PAGE) in either pH 4.8 (i.e., triplex favoring) or pH 8.0 (*i.e.*, triplex disfavoring) running buffers (Figure S5). Electrophoresis of non-cross-linked complexes at pH 4.8 revealed a significant shift in the mobility of the HP, with saturation taking place at 10 μM pso-TFO (*i.e.*, a 10-fold excess over HP), consistent with formation of HP-pso-TFO complexes in the original co-folding reaction (Figure S5A). For electrophoresis at pH 8.0, it was anticipated that only cross-linked HP-pso-TFO complexes would remain stable during the experiment, whereas uncross-linked (presumably dynamic) HP-TFO complexes would dissociate upon entering the high-pH gel. Indeed, analysis of the pH 8.0 gels revealed a significant upward shift in mobility of the HP, with saturation taking place at 10 μM pso-TFO, but only if the complexes had undergone UV irradiation, (Figure S5B). The identity of the HP-pso-TFO complexes was confirmed by comparison with a TFO containing the modified “Z” nucleobase 6-amino-5-nitropyridine-2-one (pso-TFO-Z) previously shown to improve parallel triplex formation at higher pH.^46^ The sequence specificity of pso-TFO binding was confirmed by repeating the pH 8.0 electrophoresis experiment with duplexes that differed by a single base-pair from the original HP (Figure S5C). As expected, only minor cross-linking was observed with these duplexes. Overall, these results demonstrate that specific pso-TFO-HP complexes can be cofolded and cross-linked under standard origami folding conditions. In addition, electrophoresis of TFO-HP complexes at different pH values provides a means to assay TFO-HP cross-linking.

We next investigated if HPs located in the 258HP origami structure could be loaded with pso-TFOs bearing different functional groups, and whether cross-linking led to covalent attachment of the functional groups to the hairpins in origami. We investigated binding of pso-TFOs carrying at their 3′ ends either a C6-NH_2_ group (pso-TFO-C6-NH_2_) or bulky poly(ethylene glycol) moieties (pso-TFO-PEG5K and pso-TFO-PEG20K). Such PEG modifications might protect origami from nonspecific protein binding.^47^ As with the individual HPs, we formed the TFO-258HP complexes using a co-folding strategy in which 258HP origamis were annealed in the presence of an excess of pso-TFO at pH 8.0. Experiments were undertaken using a TFO concentration of 100 μM, to achieve a ∼ 7-fold excess of TFO to individual HPs on the 258HP origami and a 2.3-fold excess over free HP staples. We then assayed the cofolded mixture by AGE at different pH (Figures 2B and S6). Analysis of the gel run at pH 4.8 revealed that, in the presence of TFOs, the mobility of the origami and the free HP staples was reduced, with the extent of the shift dependent on the guest group at the 3′-end of the TFO. The shift by the pso-TFO containing a C6-NH_2_ group was more pronounced compared to the shift by the cargo-free pso-TFO due to the higher molecular mass and charge of the 3′modification. The attachment of PEG with a molecular weight of 5,000 (PEG5K) or 20 000 (PEG20K) to TFO 3′-end further, and severely, reduced the mobility of the TFO-origami complexes (Figure S7). In all cases the shift was present with and without cross-linking, due to the TFO stabilized by Hoogsteen hydrogen bonds at the low pH during AGE. In contrast, analysis of the gel run at pH 8.0 revealed that only the irradiated (*i.e.*, cross-linked) pso-TFO-258HP complexes remained stable during electrophoresis, whereas the mobility of non-cross-linked nanostructures returned to that observed for the TFO-free 258HP nanostructure, as expected. These experiments demonstrate that CT-containing pso-TFOs can deliver diverse functional groups onto HP-origami, and that psoralen cross-linking can covalently attach the guest molecule to the nanostructure. Since the pH dependence of the CT-containing TFO binding might not always be advantageous, we also investigated the use of the Z-modified TFO (pso-TFO-Z) that works at higher pH.^46^ This time, the pso-Z-TFO-258HP complex remained stable during electrophoresis at pH 8.0, in the absence of cross-linking (Figures S8A and B). In addition, no mobility shift was observed for either the unmodified or the base-modified pso-TFO-Z when incubated with the HP-free structure, 0HP, confirming the specificity of targeting of HPs in origami by these TFOs (Figure S8C).

To further demonstrate site-specific targeting of the HPs by TFOs, we performed TEM imaging of our cross-linked origami-TFO complexes. For these and later experiments, we first purified the folded TFO-258HP complexes from the free staples and unbound TFO by ultracentrifugation at pH 4.8 (to maintain TFO-HP interactions); this strategy ensured that 90% of the 258 hairpins remained loaded with TFO after purification (Figure S9). We first imaged cross-linked PEGylated complexes by TEM, which revealed homogeneous origami particles essentially identical in shape and contrast to the TFO-free 258HP nanostructures. This showed that TFO loading did not interfere with assembly of 258HP-origami structures but did not provide additional insights into site-specific targeting of HPs, presumably due to the low contrast of the attached PEG moieties (Figures 2C and S10). To reveal site-specific targeting of HPs on the origami by the TFOs, we used pso-TFO labeled with a biotin group at the 3′ end (pso-TFO-bio), which enabled the recruitment of streptavidin tetramers (STV) to the origami-bound TFOs. For these experiments we used triangular origami that contained 27 HPs grouped into three clusters of nine adjacent HPs, with one cluster per triangle sector (27HP, Figure S11). The cross-linked and purified 27HP-pso-TFO-bio origami complex was incubated with STV for 1 h. AGE analysis at pH 8.0 revealed that the complexes were progressively shifted by STV, with apparent saturation achieved at a ratio of ∼10:1 STV to HPs in origami (Figure S12A). No STV-induced shift was observed for TFO-free 27HP (Figure S12B). Quantification of fluorescence of saturated 27HP-pso-TFO-bio:STV complexes stained with a biotin-Alexa647 conjugate revealed an ∼26.7:1 ratio of origami backbone to streptavidin, consistent with the theoretical 27:1 ratio (Figure S12C). TEM imaging of 27HP origami already revealed some contrast at the expected locations of the HPs in the origami and was therefore not used as a positive confirmation of STV recruitment (Figure S12D). In contrast, AFM images of the same complexes revealed no such HP clusters (Figure S12E) and we therefore used AFM as a means to establish positioning of the protein. Indeed, images of STV-saturated 27HP-pso-TFO-bio origami revealed triangles featuring “blobs” in three clusters at the expected locations of all three sectors (Figure 2D).^48^ We also saw no evidence of STV recruitment to the 0HP structure (Figure S12E). We conclude that TFOs can accurately target specific sites in HP-containing DNA origami and that the number and positioning of introduced groups can be varied through HP incorporation.

### Mechanism and Specificity of TFO-Directed Photo-Cross-Linking

**Figure 3:**
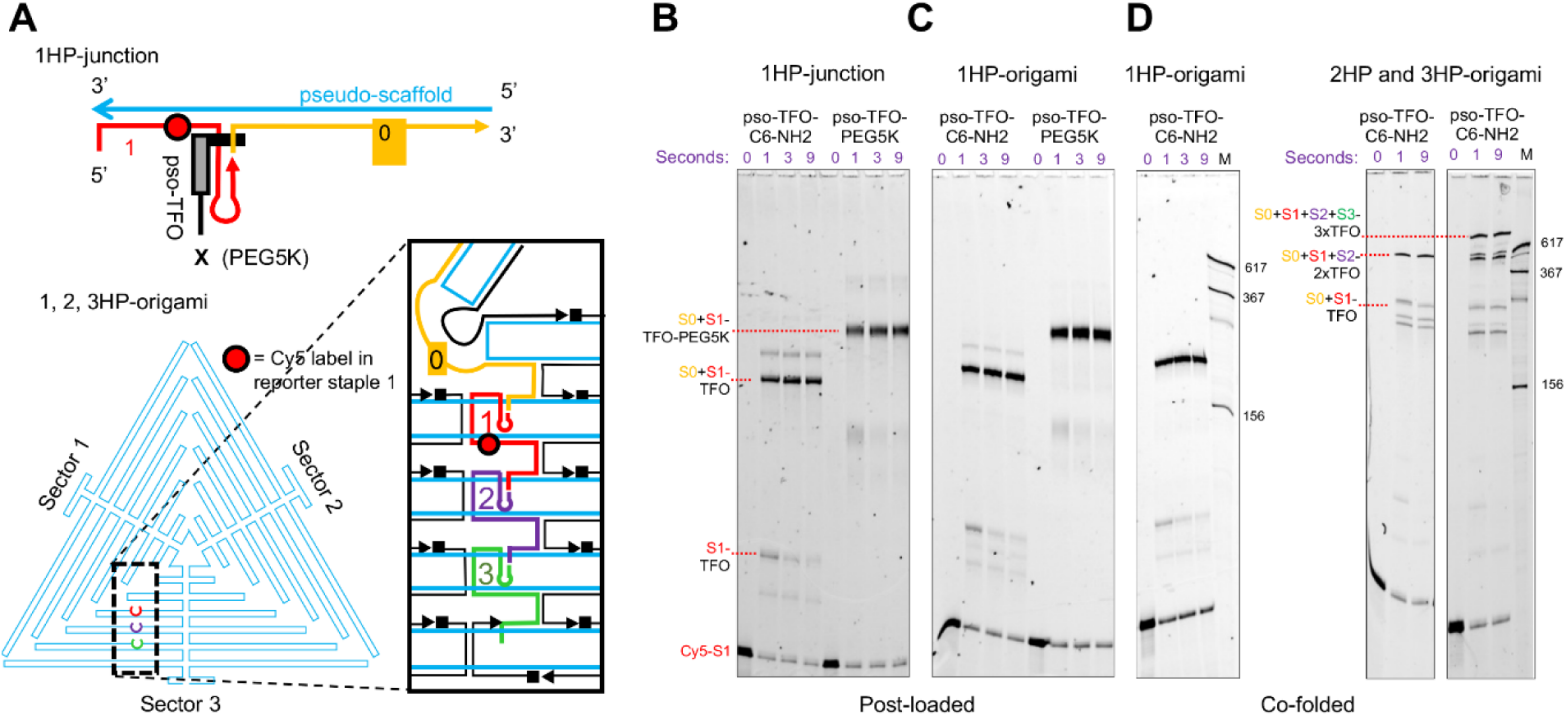
Mechanism and specificity of pso-TFO-driven cross-linking. (A) Experiments were undertaken on 1, 2, and 3HP origami containing one, two, or three TFO-binding hairpins, respectively. HP1 was assembled using 3′-hairpin-modified staples 0 and 1, HP2 – using 3′-hairpin-modified staples 0, 1 and 2, and HP3 – using 3′-hairpin-modified staples 0, 1, 2, and 3. As a control, a 1HP minimal junction (top) was assembled from staples 0 and 1 but used a minimal “pseudo-scaffold” oligo in place of the origami scaffold. Each of these complexes contained staple 0 (orange), which lacked the 3′-hairpin extension. Complexes were either postloaded or cofolded with the pso-TFOs (pso-TFO, pso-TFO-C6-NH_2_, or pso-TFO-PEG5K) as indicated. Samples were subjected to irradiation at 365 nm for the time points shown, and the products of the reaction were denatured and separated on an 8% denaturing PAGE. Bands were visualized by scanning for Cy5-labeled staple 1 fluorescence. Lane M contains linear Cy5-labeled PCR products of the indicated lengths. Full gels for these and other TFOs can be seen in Figures S13, S14. (B–C) Experiments on 1HP-origami and 1HP-minimal junction. (D) Experiments on 1HP, 2HP and 3HP-origami.

Since irradiation of pso-TFO-loaded origami with 365 nm light stabilized the interactions between pso-TFO-PEG/bio and HP-origami, we sought further insight into the cross-linking mechanism. We first analyzed the cross-linking products of pso-TFO-258HP complexes by denaturing PAGE, revealing the generation of long adducts containing multiple staple strands (“super-staples,” Figure S13). It also showed that the cross-linking reaction was complete within 10 s. In addition, the scaffold strand was not cross-linked even after 90 s of UV exposure, confirming that the “core” of the DNA origami structure remains intact upon UV irradiation. To simplify analysis of cross-linking products, we then designed a further DNA origami containing exactly one hairpin (1HP) (Figures 3A and S11). In this origami, the hairpin is formed between a reporter staple labeled with Cy5 (staple 1, shown in red) and an adjacent staple (staple 0, shown in orange). It was expected that irradiation of 1HP origami loaded with pso-TFO would result in the formation of mono- and bis-adducts between the 13-mer pso-TFO and these two staples. These adducts would then be detectable by PAGE as an upward shift in the mobility of fluorescent staple 1. To minimize the ambiguity in identifying cross-linking products, we also created a minimal three-way origami module (1HP-junction) (Figure 3A). 1HP-minimal junction contains the same hairpin formed by staples 0 and 1 but uses a short “pseudo-scaffold” to mimic the scaffold in the 1HP origami. If the mechanisms of pso-TFO-driven cross-linking within the minimal 1HP junction and the full 1HP origami were the same, we would expect to observe identical patterns of Cy5-labeled adducts in PAGE analysis of both irradiated constructs.

We first folded the 1HP-minimal junction and loaded it with an excess of pso-TFO. We then irradiated the complexes for 1, 3, and 9 s and separated the products using PAGE (Figures 3B and S14). Analysis of the gel revealed that, after 1 s of irradiation, ∼89% of the Cy5-labeled staple 1 was converted into products of higher molecular weight. The main product contained ∼60% of signal and was consistent with the formation of a bis-adduct comprised of staple 0, staple 1, and the TFO.^40,41^ An additional product of intermediate molecular weight that contained 19% of the signal likely corresponded to the monoadduct formed between the Cy5-labeled staple 1 and the TFO. To support this, it was observed that the proportion of the monoadduct decreased by ∼7% upon irradiation for 9 s, while the proportion of the bis-adduct increased by ∼8%. In addition, both products migrated slower when pso-TFO-PEG5K was used in place of the pso-TFO. Overall, these results indicate that the 1HP-junction loaded with pso-TFO gets rapidly and specifically cross-linked upon irradiation, with formation of a bis-adduct containing pso-TFO and the two hairpin-forming staples.

We then asked if 1HP origami is cross-linked by a mechanism identical to the minimal 1HP-junction. We found that, for the 1HP origami-pso-TFO complex, the patterns of the Cy5-labeled products, their mobilities, and the kinetics of their formation were essentially indistinguishable from those in the minimal 1HP junction-pso-TFO complex (Figures 3C and S14). The observed efficiencies of the bis-adduct formation increased for irradiation times of 1, 3, and 9 s (74%, 80% and 81%, respectively), while the efficiencies of the intermediate monoadduct formation decreased for the same time points (10%, 4%, and 3%, respectively). The same cross-linking patterns and kinetics were observed when we repeated the 1HP origami experiment with pso-TFO-PEG5K (Figures 3C and S14), except that the mobilities of all detected adducts were shifted according to the extra molecular weight of PEG5K, indicating that the bulky PEG5K residue did not interfere with the cross-linking. Cross-linking of 1HP-pso-TFO complexes prepared by the cofold method followed the same kinetics as cross-linking of 1HP origami saturated by pso-TFO (compare 1HP origami gels in Figure 3C,D). In all cases, the pso-TFO-dependent cross-linking of 1HP was highly specific, since no significant amounts of adducts of sizes bigger than the expected bis-adduct were detectable on the PAGE gels.

In origami containing adjacent TFO-binding hairpins, a single staple can participate in the formation of two hairpins–one hairpin at the 5′ end (formed by the 4-nt staple extension) and another one at the 3′ end (formed by the 32-nt extension). We therefore asked if “super-staples” can form upon cross-linking of adjacent hairpins. Thus, we purified origami in which we added 1 (2HP) or 2 (3HP) additional hairpins adjacent to the hairpin formed by staple 0 and staple 1 (Figures 3A and S11). These time experiments were performed on origami prepared using cofold assembly (Figures 3D and S15). It was observed that, after irradiation for 1 and 9 s, both the 2HP and 3HP origami formed cross-linked species that migrated much slower than those observed for 1HP. This is consistent with formation of a three-staple adduct for 2HP, presumably mediated by two pso-TFOs, and a four-staple adduct for 3HP, presumably mediated by three pso-TFOs. Longer irradiation times did not significantly affect the pattern of cross-linking products, indicating that the formation of superstaples went to saturation. Precise assignment of cross-linking products for 2- and 3HP is complicated by incomplete mono- (∼80%) and bis-adduct (∼60%) formation for each of the 1, 2, and 3HP structures which leads to the formation of multiple possible products (see Figure S16 for a schematic of the potential cross-linking products). We conclude that the targeting of proximal HPs in origami leads to the formation of “super-staples” and that this site-specifically tethers the pso-TFO (and attached functionality) to precise locations on the origami. We also note that irradiation of the Cy5-labeled DNA constructs for 3 s did not cause significant changes in Cy5 fluorescence in species detectable by PAGE, while irradiation for 9 s resulted in a minor ∼20% decrease in Cy5 fluorescence (Figure S17). This indicates that the 365 nm irradiation power density used in our LED setup is mostly benign to fluorophores attached to origami.

### Improving the Structural Integrity of Functionalized Origami

**Figure 4:**
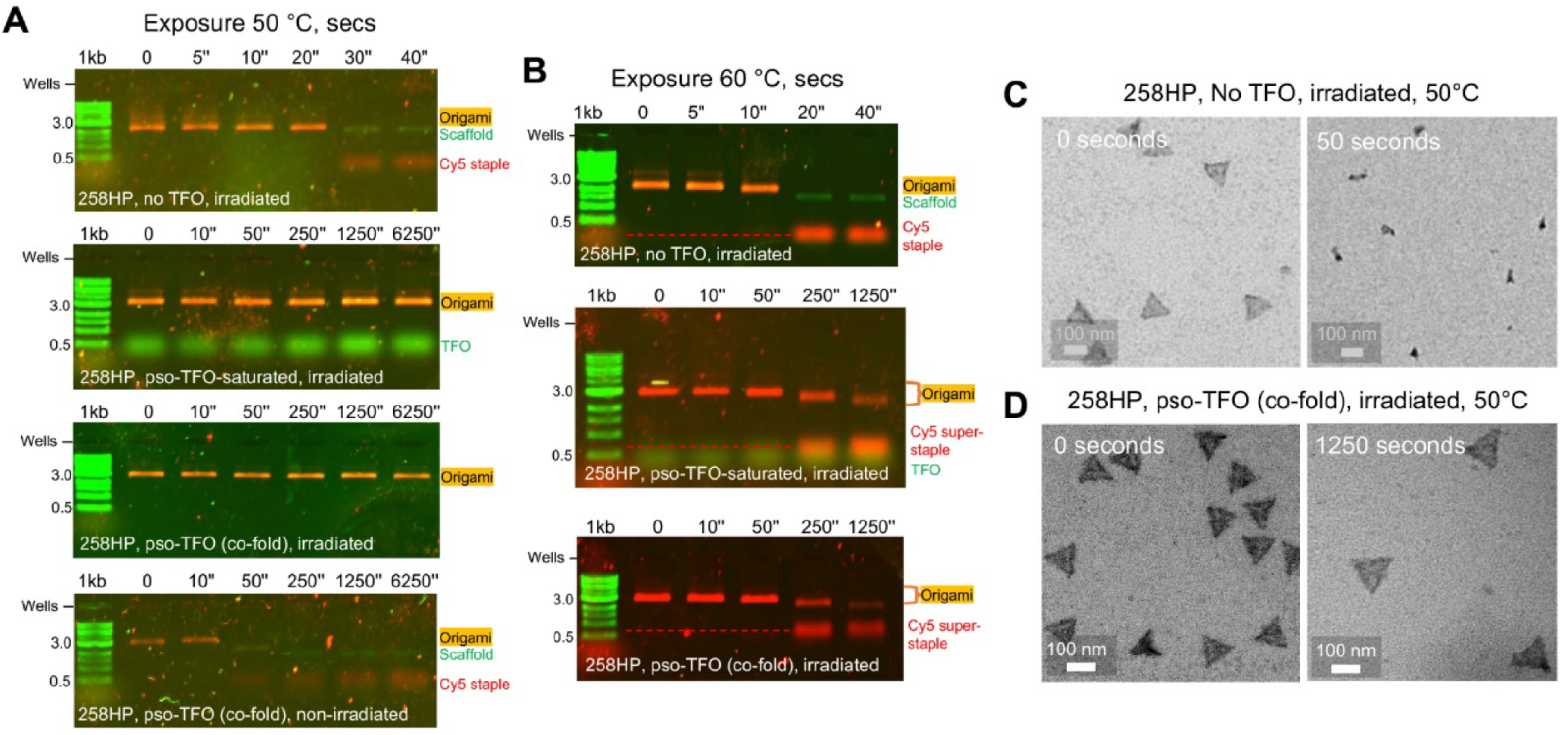
Heat challenge of 258HP origami subjected to pso-TFO driven cross-linking. (A) and (B) Experiments were undertaken on origami folded in the absence (top panel) or presence of pso-TFO-PEG_4_-NH_2_ under saturating (upper middle panel) or co-folding (lower middle and bottom panel) conditions and where indicated, subjected to irradiation for 10 s. Aliquots of 2 nM origami were then subjected to heat challenge at either 50 °C (A) or 60 °C. (B) for the time points indicated. Samples were separated on a 1% agarose gel in a pH 4.8 running buffer at room temperature. Bands for the origami and “super-staples” were visualized by scanning for Cy5reporter staple fluorescence, while the scaffold strand, free TFO, and dsDNA markers were visualized by scanning for ethidium bromide fluorescence. (C) Representative TEM images of 258HP origami prepared in the absence of TFO, irradiated, and subjected to heat challenge for 0 and 50 s. (D) Representative TEM images of 258HP origami prepared in the presence of TFO, irradiated, and subjected to heat challenge for 0 and 1250 s.

Since our cross-linking strategy leads to the formation of “super-staples” within HP-origami, we next asked if this increases the thermal stability of the functionalized nanostructures (Figures 4 and S18). We used AGE analysis to monitor origami denaturation at specific temperatures using the following as signals: (i) the release of the Cy5-labeled staple 1, which migrates below the 0.5 kb dsDNA marker; (ii) the decrease of the Cy5 signal in the origami; and (iii) the conversion of the origami into the faster migrating ssDNA scaffold. Where appropriate, AGE analysis was supported by TEM imaging of the heat-treated nanostructures.

We first determined the effect of a 50 °C temperature challenge on 258HP origami that had been irradiated for 10 s in the presence and absence of a pso-TFO carrying a PEG_4_-NH_2_ group (pso-TFO-PEG_4_-NH_2_). AGE analysis of the TFO-free origami revealed that the nanostructure underwent rapid unfolding between the 20- and 30-s time points, with no intermediates detectable within the time resolution of this assay (Figure 4A top). This agrees with previously reported studies that show that the melting of origami structures occurs cooperatively.^49^ TEM imaging also confirmed that after a 50 s incubation there were no intact origami remaining in the TFO-free origami sample (Figures 4C and S19). We next investigated if saturating and cross-linking the purified 258HP origami in the presence of a ∼ 70-fold excess of pso-TFO over HPs led to an enhancement in the stability of the complexes. Strikingly, we found that irradiated pso-TFO-loaded 258HP remained intact for at least 6250 s (∼1.7 h), consistent with at least a 250-fold increase in the lifetime (Figure 4A top middle). We also asked if complexes prepared and cross-linked using the cofold assembly method exhibited the same level of stabilization. AGE analysis revealed that the structures appeared as stable as the TFO-saturated origami, and no unfolding products were again detectable for at least ∼1.7 h of the temperature challenge (Figure 4A bottom middle). This was confirmed by TEM imaging which showed that after incubation for 1250 s the complexes remained intact (Figures 4D and S20). Finally, we investigated if cross-linking was a requirement for stabilization by assessing whether the TFO-bound but not cross-linked 258HP origami structure was unfolded in a similar fashion to TFO-free 258HP. As expected, comparison between gels revealed similar unfolding kinetics for both types of complexes, demonstrating that cross-linking is a prerequisite for improved stability (Figure 4A bottom).

To probe the limits of pso-TFO-driven stabilization, we repeated the panel of thermal challenge experiments at a higher temperature of 60 °C, using the same three types of irradiated origami. This time, AGE analysis showed that the TFO-free origami unfolded completely in 10–20 s, *i.e.*, at an expectedly faster rate than at 50 °C (Figure 4B top). In contrast, the TFO-cross-linked origami remained intact for at least 50 s, irrespective of whether the complexes had been saturated with TFO or had been prepared by cofold assembly. Further incubation at 60 °C caused partial unfolding of the cross-linked origami which was detected as (i) a ∼3-fold and 8-fold reduction in fluorescence in the Cy5-labeled origami at 250 and 1250 s, respectively, and (ii) a progressive downward shift in the Cy5-labeled origami at 250 and 1250 s (Figure 4B middle and bottom). Despite partial unfolding, the migration of both types of cross-linked origami never reached the level of the Cy5-free ssDNA scaffold, indicating that they might have retained some structural elements despite prolonged incubation at 60 °C. In addition, a Cy5-labeled species appeared below the origami band which migrated between the 0.5 and 1 kb dsDNA markers. This species is likely to be similar in nature to the “super-staples” observed in the 2HP and 3HP origami cross-linking experiments (Figure 3D). Overall, these temperature challenge experiments indicate that pso-TFO-mediated cross-linking leads to a dramatic stabilization of the HP-origami and is not affected by the method of their assembly. These experiments also confirmed that irradiated origami undergo incomplete cross-linking, as the fully cross-linked superstaple (comprised of 258 HPs) would be expected to migrate at the rate of the ssDNA scaffold (at ∼1.7 kb dsDNA) or a heavier species.

**Figure 5:**
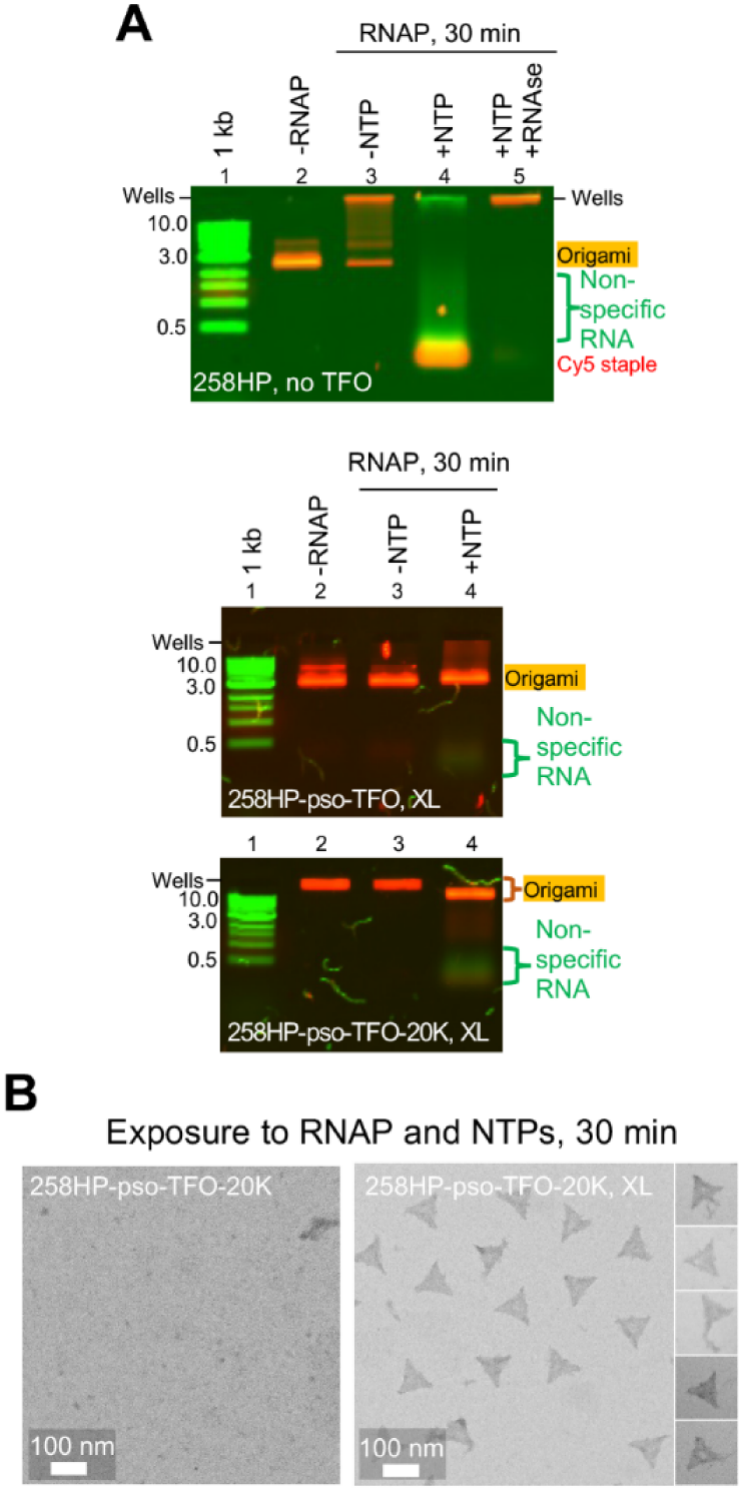
RNAP challenge of 258HP origami subjected to pso-TFO-driven cross-linking. (A) Experiments were undertaken on origami folded in the absence (top panel) or presence of a pso-TFO (middle) or its PEG20K conjugate (bottom panel). They were prepared under cofold annealing conditions, and the origami containing pso-TFOs were subjected to irradiation for 10 s. Samples of 2–5 nM folded origami were then incubated with 2.5 μM T7 RNAP in the presence or absence of 1 mM NTPs at 37 °C for 30 min and then subjected to proteinase K treatment for 15 min to degrade the protein. An additional sample was subjected to RNase A treatment before proteinase K digestion. Samples were separated on a 1% agarose gel in a pH 4.8 running buffer at room temperature. Bands for the origami and the Cy5-labeled staple 1 were visualized by scanning for Cy5 fluorescence, while the scaffold, RNA transcripts, and dsDNA markers were visualized by scanning for ethidium bromide fluorescence. (B) Representative TEM images of nonirradiated (left image) and irradiated (right image) 258HP-pso-TFO-PEG20K origami structures that had been subjected to RNAP challenge in the presence of NTPs.

We then asked if TFO-directed cross-linking improves the resistance of functionalized origami to DNA-processing enzymes (Figure 5). We chose phage T7 RNA polymerase (T7 RNAP) as a suitable test since it has been shown to induce disassembly of DNA nanostructures by promoter-independent transcription.^25^ We reasoned that formation of the “superstaples” might provide “self-healing” properties to the origami HPs and protect the structures from RNAP-dependent strand displacement. To test this, we first incubated purified TFO-free 258HP origami with a 2.5 μM T7 RNAP (500-fold molar excess), conditions that mimicked the estimated *in vivo* concentrations of commonly studied RNAPs.^50,51^ The origami was incubated with RNAP at 37 °C for 30 min and then treated with proteinase K before analyzing the samples by AGE (Figures 5A top and S21). Analysis showed that, in the absence of nucleoside triphosphates (NTPs), most of the origami became distorted, but not disassembled by RNAP, as evidenced by the appearance of a step/smear pattern from the origami band to the well in the gel and by the absence of the released Cy5-labeled reporter staple (compare lanes 2 and 3). In contrast, incubation with RNAP in the presence of 1 mM NTPs resulted in the complete destruction of the TFO-free origami, as seen from the full transition of the Cy5 signal from the origami band into the released Cy5-staple (lane 4). In addition, a strong smear detectable by ethidium bromide, migrating between the Cy5-labeled staple and the 0.5 kb dsDNA marker, appeared after incubation. Further incubation with RNase removed the smear, indicating that it represented the RNA nonspecifically transcribed by RNAP from the nanostructures (lane 5). We conclude that TFO-free 258HP origami are bound by RNAP in the absence of NTPs and are disassembled under conditions permitting transcription.^25^

We next investigated if TFO-cross-linked origami became resistant to RNAP transcription (Figures 5A middle and S22). We found that, in the absence of NTPs, the cross-linked complexes appeared intact after the RNAP challenge, indicating that, under NTP-free conditions, TFO loading and/or cross-linking reduces the affinity of RNAP for the origami (compare lanes 2 and 3). Most importantly, in the presence of NTPs, the cross-linked complexes appeared resistant to RNAP disassembly (lane 4). Interestingly, the RNA smear was still detectable after the transcription challenge, indicating that RNAP could still transcribe the cross-linked origami, but presumably could not displace the “super-staples” from the scaffold (lane 3). We thus investigated whether we could eliminate this residual transcriptional activity by attaching the bulky PEG20K to the 3′ end of the TFO (Figures 5A bottom and S22). We found that the cross-linked 258HP-TFO-PEG20K complexes were indistinguishable from the PEG-free 258HP-TFO complexes; *i.e.*, they remained intact after transcription and still supported RNAP transcriptional activity. TEM imaging showed mostly intact 258HP-pso-TFO-PEG20K complexes after transcription, with minor damage in the triangle centers where the 3 central staples lacked HPs (see Table S1) and at the sector joints (perhaps due to the low density of TFO-binding HPs at these positions) (Figures 5B and S23). We conclude that internal cross-linking of HPs by pso-TFOs and their conjugates protects HP origami from destruction by transcribing RNAP.

**Figure 6:**
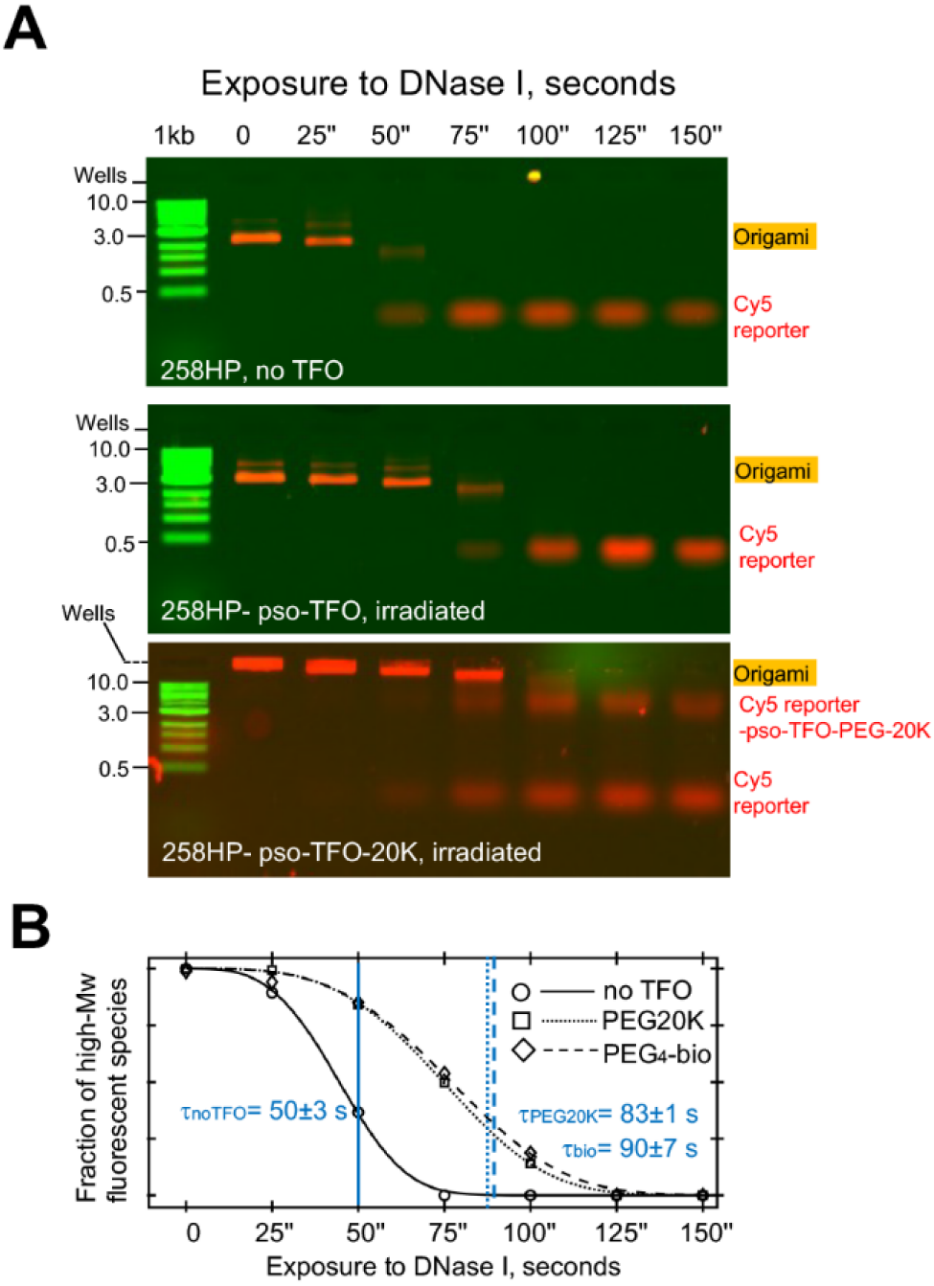
DNase I challenge of 258HP origami subjected to pso-TFO-driven cross-linking. (A) Experiments were undertaken on origami folded in the absence (top panel) or presence of a pso-TFO (middle) or its PEG20K conjugate (bottom panel). They were prepared under co-folding conditions and - were subjected to irradiation at 365 nm for 10 s. Samples of 2–5 nM folded origami were then incubated with 20 nM DNase I at 37 °C for 30 min and then stopped by addition of SDS. Samples were run on a 1% agarose gel in a pH 4.8 running buffer at room temperature. Bands for the origami and the Cy5-labeled staple 1 were visualized by scanning for Cy5 fluorescence, while the scaffold and dsDNA markers were visualized by scanning for ethidium bromide fluorescence. (B) Plots of the fractions of the surviving slow-migrating species against exposure to DNase I.

Established methods of UVB-directed nanostructure stabilization rely on indiscriminate pyrimidine dimer formation throughout the origami core.^29,30^ Although this leads to dramatic stabilization of the origami, it also alters the sequence information and the structure of the underlying scaffold,^31^ preventing read-out by DNA-binding proteins and DNA-processing enzymes^29,30^ (and thus reducing the biofunctionality of the cross-linked structure). Indeed, such structures are protected from digestion by DNase I (and other nucleases) and have been shown to be inefficiently expressed when delivered inside cells.^13^ Since our TFO-driven cross-linking reaction is directed to hairpins outside of the origami core, unwanted scaffold cross-linking by psoralen will be avoided. In addition, since cross-linking relies on low-energy UVA (365 nm), it does not promote formation of pyrimidine dimers. To demonstrate this, we assessed the susceptibility of 258HP origami cross-linked with pso-TFO-C6-biotin or pso-TFO-PEG20K to DNase I digestion. We incubated the origami with 20 nM DNase I (*i.e.*, a ∼ 10-fold molar excess over origami) at 37 °C and stopped the reaction by addition of the denaturing agent SDS. We used AGE to assess the kinetics of degradation of the nanostructures by DNase I, using as signals the decrease of Cy5 staple fluorescence in the slow-migrating origami species and the reciprocal increase of Cy5 fluorescence in the fast-migrating species at the bottom of the gel (Figure 6A). The plots of the fractions of the surviving slow-migrating species displayed a pronounced inverted S-shape for all three types of origami (Figure 6B), a behavior that could not be described by a simple single-exponential decay model. Instead, the decay plots fit quite well (*R*^2^ > 0.997) with a two-parameter Weibull distribution defined by the lifetime parameter τ and the shape factor *k* (at *k* > 1, the distribution describes breakdown of a multicomponent system at a failure rate that increases at power of *k*).^52^ The fits revealed that both TFO-functionalized origami showed only a modest ∼2-fold reduction in the DNase I decay rate compared to the TFO-free origami (τ = ∼90 ± 7, ∼83 ± 1, and ∼50 ± 3s for TFO-C6-biotin, TFO-PEG20K, and no TFO, respectively), and the complexes were completely degraded. Minor protection was expected at the triplex regions of the TFO-bound hairpins, as well as due to the presence of the 3′-modifiers. We had anticipated that the bulky PEG20K modification might provide greater protection against degradation but, as with the RNAP experiments, this was not the case. We conclude that our directed cross-linking approach does not lead to unwanted cross-linking of duplex regions located in the origami core.

## Discussion

Here, we have developed an approach that extends triplex-directed functionalization and cross-linking from DNA tiles to DNA origami.^34,37,38^ We have shown that TFOs can target hairpin duplexes introduced at single, multiple, or almost all of the nick sites between staples within a prototypical DNA origami structure, with ∼90% loading efficiency of the purified constructs. Functionalization of origami with amine and biotin groups was achieved by attaching cargo to the 3′ of the TFO and was effective even for bulky PEG5K/20K groups. Introduction of the groups was possible during or after assembly, without disruption to the underlying origami scaffold. Most importantly, we show that the TFO (and attached groups) can be covalently cross-linked to the origami with >80% cross-linking efficiency using psoralen-5′-labeled TFOs. We show that 60–80% of the adducts formed by psoralen are bis-adducts that tie the TFO to both staple strands of the assembled hairpins, and the yield of the cross-linked products was consistent with previous studies.^53^ However, the yield may vary, depending on the location of the HP within the origami structure. Directing cross-linking reactions between proximal hairpins led to the generation of “super-staples” that prevented the stochastic dissociation of the staple (and attached TFO conjugate) from the structure. Cross-linking efficiency could be improved by using TFOs containing multiple psoralen molecules (*e.g.*, “triplex staples”)^54^ or using TFOs that contain multiple stabilizing nucleotide analogues, such as the Z nucleobase used in this work.^46,55^ Such TFOs would extend applications to much higher pH (>8.0). Since psoralen cross-linking utilizes mild UVA light, we also demonstrated that efficient cross-linking was possible using fast irradiation times (<10 s) and a simple narrow-band LED as a light source which leaves the DNA origami core scaffold intact and leads to only minor photobleaching of fluorescent labels attached to the DNA.

In this study, we used HPs containing the same TFO target sequence, but future applications may require the introduction of HPs containing different sequences. For example, a typical 2D origami structure containing 250 staples would allow the introduction of ca. 250 unique HPs. However, a typical 3D origami structure would require a smaller number of unique HPs, since HPs would need to be positioned only on the outside. In either case, it will be possible to design such numbers of unique TFO target sequences as TFO selectivity is similar to the association of duplex strands and even single triplet mismatches are destabilizing (as evidenced in Figure S5C).^33−35^ Such design rules have recently been described elsewhere for both CT- and AG-motif triplexes.^56,57^ It should be noted that, compared to other noncovalent oligonucleotide-directed approaches, the covalent aspect of our functionalization approach comes at a price: the attachment of psoralen (available commercially) to the TFO. This might restrict the number of guest molecules that can be cost effectively tied to the origami structure.

In addition to functionalization, we have also demonstrated that, through the appropriate positioning of hairpins, such TFO-cross-linked structures become less susceptible to thermal denaturation and completely resistant to disassembly by concentrated phage T7 RNAP. Our RNAP sensitivity assay was inspired by the motivation to employ DNA origami structures as platforms for single-molecule analyses of transcription regulation at single-gene resolution. We anticipated that uncross-linked origami would be highly vulnerable to RNAPs, since origami, by definition, have numerous nicks and often have ssDNA modules, whereas RNAPs are known to nonspecifically initiate from nicks^58−61^ and ssDNA.^62^ Sensitivity of nanostructures to viral RNAPs has been recently documented.^25^ We showed that TFO-driven cross-linking protected origami structures from destruction by the transcribing T7 RNAP, consistent with the notion that the superstaples formed by cross-linking could not be irreversibly displaced by the transcribing enzyme. The finding that the cross-linked origami structures were still transcribed by RNAP showed that the nanostructure remained accessible to RNAP despite the presence of TFO or the functionalization of the TFO 3′ end with the bulky PEG20K group. Optimization of the hairpin placement and/or the TFO functionalities will be needed to fully control nonspecific origami binding by RNAP and other DNA-processing enzymes. We envisage that our origami structure could be site-specifically functionalized with a dsDNA fragment containing a specific promoter. Cross-linking the structure *via* the pso-TFO-driven approach would leave the origami-tethered dsDNA promoter fragment intact and available for transcription. Such structures could be used as confinement “nano-reactors” to visualize single-molecule assembly of transcription complexes, both *in vitro* and in live cells. Overall, the RNAP sensitivity assay offers more nuanced metrics of origami functionality in biological media compared to nuclease sensitivity assays,^24^ since it provides three separate read-outs – nonspecific RNAP binding/distortion of origami structures, staple displacement, and RNA production.

While other UV-based cross-linking strategies (*e.g.,* welding or the use of free psoralen) could be used to stabilize Watson–Crick functionalized staple extensions, they would do this at a cost to the sequence and structural integrity of the underlying origami scaffold, *i.e.* through indiscriminate cross-linking of TpA (psoralen) or TpT (welding) steps throughout the structure.^20,29−31^ This would be problematic, for example, for downstream applications that require read-out or manipulation of the duplex regions of the origami core by DNA-binding/processing enzymes (*e.g.,* for gene delivery and expression).^13^ By contrast, our approach requires both the presence of a TpA step (for the psoralen) and a binding sequence (for the TFO) for cross-linking. Since they are only present on the hairpin staple extensions, the pso-TFO does not lead to unwanted scaffold cross-linking. Indeed, we see no evidence of nonspecific TFO cross-linking throughout this study, and we also see no evidence that our scaffold is damaged using low energy UVA light. We also demonstrate that, unlike structures that have been UV welded,^29^ the underlying duplex regions of the origami complex are susceptible to DNase I digestion. This suggests that they can still be read and manipulated by DNA-binding proteins.

More recently, triplex motifs have also been developed as secondary structural elements capable of compacting dsDNA into various new DNA origami architectures.^34,56,57^ Such structures are assembled from designer scaffolds containing multiple distinct polypurine–polypyrimidine TFO target sequences folded by triplex-mediated crossover strand exchange. Our functionalization strategy could extend this design process, *e.g.,* by attaching psoralen and other cargo to the crossover TFOs. The combination of such triplex technologies would result in a new generation of functionalized origami nanostructures that exhibit enhanced configurability and stability over those obtained with their duplex-only counterparts. This is likely to have the most benefit with origami structures designed for use in biomedical science, where maintaining the structural integrity and function of the underlying DNA is paramount.

## Supporting Information Available

Contains experimental methods, oligonucleotide and staple sequences, and additional AGE, PAGE, TEM, and AFM data (PDF)
