# Supplementary material for: Functionalizing DNA Origami by Triplex-Directed Site-Specific Photo-Cross-Linking
Source: J Am Chem Soc. 2024 May 2;146(19):13617–28. doi: 10.1021/jacs.4c03413 (PMC11100008; doi:10.1021/jacs.4c03413)
Supplement: Supplementary file 1 — ja4c03413_si_001.pdf [file ja4c03413_si_001.pdf]

Supporting Information

Functionalizing DNA Origami by Triplex-Directed Site-Specific Photo-Crosslinking

Shantam Kalra,[1$] Amber Donnelly,[1$] Nishtha Singh,[1$] Daniel Matthews,[1] Rafael del Villar-Guerra,[1**] Victoria Bemmer,[2] Cyril Dominguez,[1] Natalie Allcock,[3] Dmitry Cherny,[1] Andrey Revyakin,[1*%] and David A. Rusling[4*]

[1]Department of Molecular and Cell Biology, and Leicester Institute of Chemical Biology, University of Leicester, Leicester, LE1 7RH, UK.

[2]Centre for Enzyme Innovation, School of Biological Sciences, University of Portsmouth, Portsmouth, Hampshire, PO1 2DY, UK.

[3]Core Biotechnology Services Electron Microscopy Facility, University of Leicester, Leicester, LE1 7RH, UK.

[4]School of Medicine, Pharmacy and Biomedical Sciences, University of Portsmouth, Portsmouth, PO1 2DT, UK.

[$]Equal contribution

**Present address: Biopharmaceuticals Development, R&D, AstraZeneca, Cambridge, UK.

[%]Present address: Max Planck Institute of Immunobiology and Epigenetics, Stübeweg 51, D-79108 Freiburg, Germany

*Corresponding authors: revyakin@ie-freiburg.mpg.de, david.rusling@port.ac.uk

Materials and Methods

Unless noted otherwise, salts, acids, bases, buffer components, solvents and other consumable chemicals were from Sigma-Aldrich. Water-based buffers were prepared using deionized water (diH_2O) (18.2 MΩ·cm, ELGA) and filtered with 0.2 μm disposable filter units before use (Starlab).

Origami design and synthesis. Staple routing for 0HP and 258HP origami was designed by Tilibit Nanosystems. Staple routing was visualized and modified in CadNano 2 (square lattice layout).[1] Staple strands were provided by Tilibit Nanosystems. Staples were shipped as solutions in diH_2O at 100 μM or 200 μM. Two sets of staples were synthesized – a set of 261 extension-free staples in three 96-well plates (**Table 1**) and another set of 261 control staples (258 of which were with 5′ and 3′ extensions) in three 96-well plates.. CadNano source file is available upon request.

Folding and agarose gel electrophoresis of origami. Origami was folded in a 50 μL or 100 μL reaction prepared using high-pH origami buffer (OB-8.0: 18 mM $MgCl_2$, 1 mM EDTA, 10 mM Tris-HCl, pH 8.0) or low-pH origami folding buffer (OB-4.8: 18 mM Mg-Acetate, 10 mM Tris-acetate, pH 4.8). Folding was carried out in a hot-lid thermal cycler (80°C for 10 min, then cooling from 80°C to 20°C at 0.2°C per min) in clear thin-wall 0.2 mL PCR tubes (Starlab). Folding mixtures and co-fold loading mixtures contained 50 nM ssDNA scaffold 8064 (Tilibit Nanosystems), 150 nM pre-mixed staples, and 1 mM triplex-forming oligonucleotide (TFO where necessary). Origami was visualized using electrophoresis in 1% agarose (Melford) mini-gels (~75x75 mm) at room temperature in a buffer comprised of 12 mM Mg-Acetate, 50 mM Tris-acetate (pH 8.0 or 4.8, AGEB-4.8 or AGEB-8.0, respectively), and 0.5 μg/mL ethidium bromide. During agarose gel electrophoresis (AGE, 50V), the tank was cooled with a 120mm USB-powered PC enclosure fan (Eluteng) placed next to the tank. Temperature of the gel was checked periodically with a non-contact infrared thermometer (Fluke) and typically remained

under 24°C during 2-3 hours of electrophoresis. The magnesium hydroxide precipitate was periodically scraped off the cathode with a plastic pipette to ensure stable current. The gels were scanned using the Typhoon scanner (Cytiva) at 100 μm per pixel, using built-in Cy5 settings for reporter Cy5-labeled staple 1 and built-in Cy3 settings for ethidium bromide. Gels were quantified in ImageJ (NIH) and Excel (Microsoft).

Folding of oligonucleotide containing only HP sequence (Fig S5) was carried out in OB-8.0 buffer containing 1 μM HP in the presence of 0.001-100 uM TFO as indicated.

- HP oligonucleotide: 5′-GC**T**AAGAAARAAGAGAaaaaTCTCTTYTTTCTT**T**AGC-3′ (Sigma, UK)

Unlike other HP sequences used in this study this oligo contained the full TpA step for crosslinking (highlighted in bold). The standard target contains a GC base pair at position RY (underlined) and is a perfect match for the TFO sequence. For selectivity experiments, similar sequences were used that contained the remaining CG, AT, and TA base pairs at this position. generating mismatched triplet C-CG, C-AT, and C-TA.

Annealing was carried out using the same thermal cycler program as the one used with DNA origami. Samples were used without further purification.

Folding of 1HP minimal junction (Fig 3B) was carried out in OB-8.0 buffer containing 0.4 μM final concentration of each of the following three oligonucleotides:

- 1HP minimal scaffold oligonucleotide: 5′ GCAAGGTTGTTTCATCATCAAAAAAAAAAAAAAAAAAAAAATTGCCATCATTTCGGGCAAAGGATTTATTATCTATTGACGGCGAGGGTTT-3′, Integrated DNA technologies, IDT,

- Cy5-labeled reporter Staple 1 (3-84 in Table 1, has the 4-nt 5′-TAGC-3′ extension and the 32-nt TFO hairpin extension, Tilibit Nanosystems),
- Reporter Staple 0 (3-51 in Table 1, has the 5′-TAGC-3′ extension and no 32-nt TFO hairpin extension, Integrated DNA technologies).

Annealing was carried out using the same thermal cycler program as the one used with DNA origami. Annealing of the minimal 1 HP junction was verified using AGE alongside staple 1 annealed by itself (scanning with default Cy5 settings), and the 1HP junction was used without further purification.

Purification of origami. 10-50% glycerol gradients based on the OB-4.8 or the OB-8.0 buffer were prepared in 4 mL Beckman polypropylene round-bottom ultracentrifuge tubes using a Gradient Master 107 gradient maker (BioComp). 50-100 µL of origami folding mixture were loaded on top of the gradient using a 20 µL pipet, and the tube was placed into a pre-cooled ultracentrifuge bucket, which was then placed into a TH660 swing-bucket rotor (Thermo Fisher Scientific). Centrifugation was for 1.5-2 hrs at 47,500 RPM, at 4°C (Sorvall Discovery 100 SE). If the origami contained the Cy5-labeled staple 1 reporter, the peak fraction (corresponds to the monomer origami) was typically found below the middle of the gradient[7] and was visualized as a deep red fluorescent band using a hobby-grade fan-cooled 638 nm laser diode module powered with a 12V AC-DC power supply (Ebay) and 633nm notch filter (Semrock). Origami fractions were withdrawn from the top of the gradient at 200 µL per fraction, analysed using AGE, and the nucleic acid (NA) concentration in fractions was quantified using a Nanodrop spectrophotometer (Thermo Fisher Scientific) by measuring optical density at 260 nm. Peak fractions containing origami monomers (typically at 2-5 nM) were stored at -20°C and used for AGE, temperature and RNAP challenges and TEM imaging.

Loading of 258HP origami with TFO-A647 and estimation of loading efficiency. Co-fold conditions were used to fold 50ul of 258HP-TFO-A647 complex in OB-8.0 as described above. The complexes were loaded onto a 10-50% glycerol gradient based on OB-4.8 and centrifuged

for 2 hours as described. Top 2 ml of the gradient was carefully removed with a pipet, and three 200 μl fractions containing the blue band in the lower half of the gradient (visible to the naked eye against white background) were merged. Optical density at 260 nm (OD_{260}) was measured using Nanodrop spectrophotometer (ThermoFisher), and origami concentration was calculated assuming 50 μg/ml for OD_{260}=1 (for origami core and hairpins) and 33 μg/ml for OD_{260}=1 for ssDNA (for TFO). To estimate TFO-A647 loading efficiency, the 258HP-TFO-A647 purified fraction was analysed using AGE (pH 4.8) alongside standards of TFO-A647 in the same buffer (OB-4.8, 25% glycerol) and of the same volume as the origami. Standard TFO-A647 concentrations were typically 62.5, 125, 250, 500, and 1000 nM. The gel was scanned in the Cy5 channel of the Cytiva Typhoon scanner, and intensities of the origami band and the standard TFO-A647 bands were quantified and corrected by background subtraction in ImageJ (NIH). Data of background-subtracted fluorescence intensity versus TFO-A647 concentration were fit to a linear regression (Fig S9), giving the estimated concentration of Alexa Fluor 647 in the origami band. Theoretical ratio of Alexa Fluor 647 molar concentration to origami molar concentration at 100% loading was 259:1 (includes the additional Cy5-labeled staple 1)

Synthesis and purification of pso-TFO-PEG5K and pso-TFO-PEG20K. To 45 μL of 1 mM solution of pso-TFO-C6-NH_2 in diH_2O (Fidelity Oligos, Gaithersburg, USA) was added 5 μL of 1M $NaHCO_3$ (pH 9.0) and 5 mg of solid mPEG-Succinimidyl Valerate (MW 5,000) or mPEG-Succinimidyl Valerate (MW 20,000) (Laysan Bio, Arab, USA). The reaction was thoroughly mixed and left at room temperature for 3 hrs (overnight incubation did not improve the yield). The reaction volume was then brought to 800 μL (*ca.* 50 μM oligonucleotide) with 0.1 M $NaHCO_3$ (pH 9.0) and the reaction was left at -20°C until purification. PEG-modified oligonucleotides were purified on an analytical HPLC system (UltiMate 3000 UHPLC, Thermo Fisher Scientific) equipped with an analytical manual injector, an analytical binary pump (UltiMate HPG-3200SD), a variable wavelength detector (UltiMate 3000 VWD), a

thermostatted column compartment set at 30°C (TCC-3000RS) and an automated fraction collector (AFC-3000). Buffer solutions were prepared with diH_2O and filtered as described above. Purification was by anion exchange HPLC chromatography with an analytical column (DNAPac PA-100 BioLC, 4×250 mm; Thermo Scientific) coupled with a safeguard precolumn (DNAPac PA-100 BioLC, 4×50 mm; Thermo Scientific). Before injection, the columns were equilibrated for 15 min with 100% buffer A at a flow rate of 1mL/min. After equilibration of the columns, ~400 μL of sample (*ca.* 50 μM oligonucleotide) were manually injected into the system with a 400 μL sample loop and the purified samples were eluted and collected as 1 mL fractions at a flow rate of 1mL/min. The gradient elution program consisted of a linear gradient from 0 min (100% buffer A) to 30 min (50% buffer A, 50 % buffer B). After the elution of the sample, the column was washed with 60% buffer B (10 min) followed by a re-equilibration of the column with 100% buffer A for 15 min and injection of the next portion of the sample. The compositions of the elution buffers were: buffer A (25 mM Tris-HCl, pH=7.0) and buffer B (25 mM Tris-HCl, 2M NaCl, pH=7.0). The unreacted PEGylation reagent remained in the flow-through, the PEG-modified oligo typically eluted at ~7 min of the gradient, and the unreacted oligo typically eluted at ~14 min of the gradient. Peak fractions containing the PEG-modified oligo were merged, dialyzed against 2L of diH_2O twice using a membrane with 10 kDa cut-off (Spectrum Labs), lyophilized, dissolved at 1 mM, and stored in aliquots of 5 μL at -80°C until further use. Typical yield was 30-50%. Additional amount of PEG-modified oligo could be obtained by dialyzing and lyophilizing the purified unreacted amine-modified oligo (50-70%) side by side with the purified modified oligo, and repeating the reaction as described.

Crosslinking of oligonucleotides, origami, and minimal 1HP junction. Pilot experiments with minimal HP junctions were carried out with a commercial crosslinking setup[2] equipped with an add-on bandpass filter (Edmund optics, Hoya B-370). The commercial setup required irradiation of a minimal HP junction for 10-30 minutes for the formation of the bis-adduct which

was deemed impractical for this work. Thus, a crosslinking light source was home-built around a 365 nm light-emitting diode (LED) mounted on a 20 mm star-shaped metal-core printed circuit board (110° viewing angle, RS Components "LZ4-44UV00-0000 LedEngin LZ Series 4-UV LED Array, 2000 → 3800mW"). The LED was mounted on a round finned aluminum LED heat sink (Thorlabs) and driven with a 15.2V home-built DC power supply [20V laptop AC-DC power supply equipped with a DC-DC regulator (Anyvolt, Dimension Engineering]) at 0.9A (monitored by a RC hobby Amp meter), controlled with a manual on/off switch (RS Components). Irradiation was carried out in a cold room for the times specified (using a laboratory timer). For irradiation, 2.5-15 µL of sample mixture (after folding or after purification) were placed at the bottom of a thin-wall clear 0.2 mL PCR tube. If larger volumes were to be crosslinked (*e.g.,* 50-100 µl of co-fold folded mixtures), the sample was divided into 15 µL aliquots in separate PCR tubes, and each aliquot was crosslinked separately. In an early protype of the crosslinking setup, two aspheric lenses (Edmund Optics #88-284, 12 mm diameter, 7.5 mm focal length) and a bandpass filter (Edmund Opics, Hoya B-370, 12 mm diameter) connected with 0.5-inch and 1-inch lens tubes (Thorlabs) were used to collect, filter, and focus the 365 nm light onto the origami sample at the bottom of the PCR tube. However, measurements with a 365 nm lux meter (DELO, 9 mm pancake sensor) showed that placing the sample (located in the conical tip of the 0.2 mL PCR tube) directly next to the LED emitter window (0.5 mm from the centre of the emitter) provided the highest power, fastest crosslinking and most reproducible placement of the sample with respect to the emitter. Thus, all crosslinking experiments reported in the manuscript were performed with the optics-free setup (Fig S4). Tube holder was a custom-machined (Ultraturn Engineering Ltd, Manchester, UK) tight-fitting aluminium bushing (12 mm OD) which ensured reproducible placement of the PCR tube with respect to the emitter and acted as an additional heat sink. The position of the tube holder with respect to the emitter was aligned with an basic XYZ translation stage (Thorlabs). Temperature of the LED heat sink was monitored with a contactless thermometer, and the setup was cooled by blowing air from a 75mm axial extractor fan (Manrose, UK) for few seconds, if necessary. For eye safety, during irradiation the setup was covered with a box

home-made of 5mm-thick orange acrylic sheets (CutMyPlastic, UK) glued together with dichloromethane.

Loading with streptavidin and AGE analysis of 27HP-pso-TFO-biotin origami. 27HP-pso-TFO-biotin origami were prepared by co-folding, *i.e.,* by mixing 50 nM phage scaffold, 150 nM staples and 100 μM pso-TFO-PEG$_4$-biotin (pso-TFO-bio). Upon folding, the mixtures were crosslinked by 365 nm irradiation for 10 seconds and purified by ultracentrifugation in OB-4.8 as described. Formation of hairpins and loading of pso-TFO-bio were verified by detectable mobility shifts in AGE analyses (AGEB-4.8) alongside 0HP origami and TFO-free 27HP origami. 10x solutions of streptavidin were prepared by diluting a 5 mg/ml stock solution of recombinant streptavidin in diH$_2$O (Thermo Fisher #21122, stored in single-use 1 µl aliquots at -80°C to ensure reproducibility) with OB-8.0 containing 25% glycerol. To 10 µl of 3-5nM purified 0HP, 27HP, or 27HP-pso-TFO-bio origami sample were added 1 μl of 0.5 M Tris-HCl (pH 8.0) and 1 μl of 10x stock solutions of streptavidin to set the final streptavidin concentration at 0.1, 0.3, and 1 μM. The reactions were incubated at room temperature for 30 min. Then, 1 µl of 50 µM solution of a DNA oligonucleotide modified with biotin and Alexa 647 (5′-BiosG-AATGTGAATAAAGGCCGGATAAAACTTGTGC-Alexa647-3′, Integrated DNA Technologies (IDT)) in 0.5x OB-8.0 containing 25% glycerol was added to the reaction, and the reaction was incubated for an additional 20 min. The reactions were then directly (no additional dyes or buffers) loaded onto a 2% agarose mini-gel (prepared with AGEB-8.0) and ran in AGEB-8.0 at 60V with a cooling fan. The gel was stopped when the free Alexa Fluor 647/biotin-labelled oligo (visible as a blue blob by eye) ran out of the gel, while the free streptavidin (labelled with the oligo and visible as a smaller blue blob in the 1 uM streptavidin lane) remained in the gel. The gel was scanned with the Typhoon scanner (Cytiva) in the Cy5 and Cy3 channels. The origami bands fluorescent in the Cy5 channel were quantified in ImageJ using rectangular ROI of the same size for each band (raw integrated signal), with subtraction of background (ROI of same size) defined separately for each lane. The ratio of the Cy5 fluorescence signal of the

streptavidin-free 27HP-pso-TFO-bio origami (in which fluorescence was emitted only by the Cy5-labeled reporter stable) to the streptavidin-bound 27HP-pso-TFO-biotin (in which fluorescence was emitted by the reporter staple and by the streptavidin-bound Alexa647/biotin-labelled oligo) was calculated and compared to the theoretical ratio of 1:(27+1). The theoretical ratio is based on the assumption that Alexa Fluor 647 and Cy5 emit at the same intensity, and that each origami-bound streptavidin tetramer can accept exactly one Alexa Fluor 647/biotin-labelled oligo.

Atomic force microscopy (AFM) of 27HP-pso-TFO-biotin origami loaded with streptavidin. A streptavidin solution was first prepared in OB-8.0 buffer and then added to 3-5nM purified 0HP and 27HP-pso-TFO-biotin origami at a final concentration of 1 µM. The reaction was then left to equilibrate at room temperature for 30 min. Samples were spotted onto freshly cleaved mica and allowed to adsorb for 5 min. Buffer salts were removed by addition of 5–10 drops of diH_2O; the drop was shaken off and the sample dried using compressed air. Imaging was undertaken by tapping mode in air on a Digital Instruments (Bruker) Multimode AFM with a Nanoscope VI controller using silicon probes (Nanosensor PPP-NCHR k=42 N/m).

Denaturing electrophoresis of crosslinked 1, 2, 3HP origami and the 1HP minimal junction. 2.5 µL of sample was crosslinked in a thin-wall PCR tube as described above, mixed with 10 µL of deionized formamide containing 10 mM sodium hydroxide and 0.1% bromophenol blue dye, heated for 3 min at 95°C in a dry heat block with tight-fitting slots, and then placed on ice. 20 cm x 20 cm x 1 mm 8% polyacrylamide gel (19:1 acrylamide:bis acrylamide) containing 8M urea in 0.5x Tris-borate-EDTA buffer[3] was pre-run at 40W until temperature reached 50°C. After loading the 12.5 µL of samples, the gel was run with two heat sinks (10 cm x 20 cm x 2 mm aluminium plates) at 40W. To monitor progress, 1 µL of 1 µM Cy5-labeled staple 1 was run alongside the samples, the gel was monitored using the 638 nm diode laser (see above) and was stopped when the Cy5-labeled reporter staple was ~20 mm

from the bottom of the gel (about 1 hr). The gel was carefully transferred onto the Typhoon scanner plate using a 1000 µL pipet tip (the scanner plate was pre-wet with diH_2O to avoid cracking and breaking of the gel) and scanned for fluorescence using standard Cy5 settings.

Quantification of band intensities in denaturing gels to estimate crosslinking efficiencies at different irradiation times. Band intensities in the Cy5 fluorescence scan of the gel were measured using the Image J "ROI" tool and the crosslinking efficiencies were quantified in Excel. For each time point of irradiation (1, 3, and 9 seconds), rectangular regions of interest (ROI) of the same size were positioned manually around bands corresponding to the following five species: un-crosslinked Cy5-labeled staple 1, the mono-adduct, the bis-adduct, and two minor bands migrating just below the mono- and just above the bis-adduct. For each ROI, a background ROI of the same size was positioned in the same lane. The raw integrated intensities of each band (based on a 16-bit grayscale image) was calculated in Image J and imported into Excel. Then, the intensity of each band was corrected by subtracting the respective background ROI intensity, and the intensities of the five corrected signals were summed up to obtain the total signal per lane. This signal was then assigned to unity (or 100%) for each time point. The percentages of each band intensities were then calculated using the total signal as 100%. The combined intensities of the two minor contaminating bands typically added up to about 10-11% which explains why the total reported yields of the mono- and bis-adducts do not appear to add up to 100%.

Temperature challenge of origamis was carried out in a thermal cycler pre-heated and running at constant temperature (50°C or 60°C) with a heated lid. 15 µL of purified origami (2-5 nM) in OB-4.8 was divided into six 2.5 µL samples in individual thin-wall 0.2 mL PCR tubes. Tube 1 was kept on ice as unchallenged sample. The remaining five tubes were simultaneously (to within 1 second) placed into the preheated thermal cycler and pressed in with the heated lid. Tubes were then removed at time points indicated, one by one, and placed on ice. The samples were loaded onto a 1% agarose gel (AGEB-4.8) without additional

dyes/loading buffers and electrophoresed for 2-3 hours. Alternatively, the challenged samples were used in TEM (see below).

RNA polymerase (RNAP) challenge of origamis. T7 RNAP was expressed and purified as an N-terminal HaloTag fusion using Ni-NTA (Qiagen) chromagrography.[4] Peak Ni-NTA protein fraction was dialyzed overnight against dialysis buffer (50 mM Tris-HCl pH 7.9, 100 mM NaCl, 14 mM β-mercaptoethanol, 1 mM EDTA, 10% glycerol, 1mM phenylmethylsulfonyl fluoride). After dialysis, RNAP concentration was measured against standard concentrations of bovine serum albumin (New England Biolabs) using Bradford Dye Reagent (Bio-Rad Laboratories). The protein was diluted to 25 µM using dialysis buffer, flash-frozen in liquid nitrogen, and stored at -80°C in single-use 5 µL aliquots (to ensure reproducibility). RNAP challenge was carried out in 0.2 ml PCR tubes in a preheated tight-fitting aluminium rack to ensure optimal heat transfer. RNAP challenge was carried out directly in 10 µL of origami solutions containing 2-5 nM origami (ultracentrifuge fraction in OB-4.8 containing ~25% glycerol). Prior to RNAP challenge, pH of origami was adjusted by adding 1M Tris-HCl (pH 7.9) to 50 mM. In addition, reactions contained 2.5 mM dithiothreitol, 1 mM of each nucleoside triphosphate [ATP, CTP, UTP, and GTP (New England Biolabs)], and 2.5 µM RNAP. After incubation for 30 min at 37°C, 1 µL of proteinase K solution (Abcam 281339) was supplied, and the reaction was incubated for an additional 15 min at 37°C. Then, the reaction was transferred on ice, and loaded directly onto AGE (pH 4.8) or used for TEM imaging (see below). When needed, 1 µL of RNAse A (New England Biolabs) was supplied prior to proteinase K treatment, and the sample was incubated for 15 min at 37°C.

DNase I challenge of origamis. The volume of the master mix for the DNA I challenge (at t=0 sec) was 42 µL and contained 2 nM purified DNA origami (ultracentrifuge fraction in OB-4.8 containing ~25% glycerol), 2.5 mM $MnCl_2$, and 1.4 units of DNase I (New England Biolabs). The reaction was carried out in a 0.2 mL thin-wall PCR tube which was placed into a tight-fitting aluminum rack at 37°C at t=0 sec. At time points indicated, 4.8 µL of the reaction were

quickly transferred to a PCR tube on ice containing 1μL of 0.5% solution of sodium dodecyl sulphate in diH_2O and rapidly mixed. Once all time points were collected on ice, the samples were loaded directly onto AGE (pH 4.8) or used for TEM imaging (see below).

Transmission electron microscopy (TEM). Samples for microscopy were prepared as described,[5,6] with minor modifications. Carbon films 3-4 nm thick were deposited onto 600 mesh EM grids and activated by glow discharge in air, making them hydrophilic and negatively charged (250V, 25 mA, 75 sec, Q150T PLUS coating unit), allowing for adhesion of origami molecules in the presence of magnesium ions. DNA origami samples were ultracentrifugation fractions in OB-4.8/OB-8.0 containing ~25% glycerol, or samples challenged with heat or RNAP. Origami sample was diluted 5-10-fold in deposition buffer (0.5xOB-4.8 or 0.5xOB-8.0) to 0.2-0.5 nM final origami concentration. A 20 μl droplet of diluted origami sample was placed onto a Parafilm sheet (Bemis), and an activated grid was placed on top of the droplet for 5 min. After that, the grid was briefly rinsed with deposition buffer (by touching a 20 μl droplet of the buffer) and rinsed for few seconds with aqueous solution of 2% UO_2Ac (by touching a 20 μl droplet of the solution). The rest of the stain was removed by blotting with filter paper. The procedure ensured positive staining of origami particles, though, in rare instances, islands containing negatively stained particles were observed as well. Samples were analysed with JEM 1440 electron microscope (Jeol, Japan) operated at 120 kV equipped with EMSIS Xarosa 20MP camera operated with Radius software. 16-bit TIFF images were processed in ImageJ.

Identities of staples for different origami variants. Sequences of 261 DNA origami staples for the 3-sector triangle are shown in **Table 1**, free of 5′ and 3′ extensions. Start and end of each staple in the table correspond to the helix/nucleotide at the staple 5′ and 3′ ends, per CadNano 2 output (square lattice layout[1]). Staple names include the sector ID (1, 2, or 3) and the unique ID of the staple (in order of output by CadNano). Staples relevant for folding of

1HP, 2HP, 3HP, and 27HP origami are highlighted in black (also see **Fig S11**). Staples 0, 1, 2, and 3 are highlighted with the same colours as in Figs 1B and 3A.

To fold 0HP origami, an equimolar mixture of all 261 staples in Table 1 (all extension-free) was prepared.

1HP origami equimolar staple mixture was comprised of (also see Supplementary Figure S11):

- Staples in Table 1 (259 extension-free), except 2 staples (3-51 and 3-84).
- 3-51 (reporter Staple 0) with 5′-TAGC-3′ (hairpin linker, no TFO hairpin) at 5′.
- 3-84 (Cy5-labeled reporter Staple 1) with 5′-TAGC-3′ (hairpin linker) at 5′ and 5′-GCTAAGAAAGAAGAGAAAAATCTCTTCTTTCT-3′ (TFO hairpin) at 3′.

2HP origami equimolar staple mixture was comprised of (also see Supplementary Figure S11):

- Staples in Table 1 (258 extension-free), except 3 staples (3-51, 3-57, 3-84).
- 3-51 (reporter Staple 0) with hairpin linker and no TFO hairpin;
- 3-84 (Cy5-labeled reporter Staple 1) with hairpin linker and TFO hairpin;
- 3-57 (reporter Staple 2) with hairpin linker and TFO hairpin;

3HP origami equimolar staple mixture was comprised of (also see Supplementary Figure S11):

- Staples in Table 1 (257 extension-free), except 4 staples (3-51, 3-57, 3-84, 3-15).
- 3-51 (reporter Staple 0) with hairpin linker and no TFO hairpin;
- 3-84 (Cy5-labeled reporter Staple 1) with hairpin linker and TFO hairpin;
- 3-57 (reporter Staple 2) with hairpin linker and TFO hairpin;
- 3-15 (reporter Staple 3) with hairpin linker and TFO hairpin;

27HP origami equimolar staple mixture was comprised of 234 extension-free staples in Table 1 and the following 27 staples (9 staples per triangle sector) containing the 5′-TAGC-3′ extension at the 5′ end (hairpin linker) and the 5′-

GCTAAGAAAGAAGAGAAAAATCTCTTCTTTCT-3′ at the 3′ end (TFO hairpin) (also see Supplementary Figure S11):

> Sector 1: 1-5, 1-19, 1-24, 1-26, 1-30, 1-37, 1-43, 1-53, and 1-86;
>
> Sector 2: 2-12, 2-18, 2-20, 2-25, 2-44, 2-48, 2-57, 2-65, and 2-86;
>
> Sector 3: 3-1, 3-15, 3-15, 3-34, 3-49, 3-51, 3-57, 3-74, and 3-84.

To fold 258HP origami, 258HP DNA origami staple sequences were obtained by adding 5′-TAGC-3′ to the 5′ end and 5′-GCTAAGAAAGAAGAGAAAAATCTCTTCTTTCT-3′ to the 3′ end of each staple in Table 1, except the first three staples in the table (“hole-1”, “hole-2”, and “hole-3”).

Table 1. Sequences and coordinates of DNA origami staples

Start	End	Name: -sector-ID	Sequence (free of 5′ TAGC and 3′ TFO hairpin extensions)	Description	Other
**31[248]**	1[215]	-hole-1	GAAACAATCGGTTTTCACGACGTTG	Always hairpin-free	central hole
**15[248]**	31[215]	-hole-2	GGGAGAAACAATTTAACAGAAATAA	Always hairpin-free	central hole
**1[248]**	15[215]	-hole-3	ATGAATTTTCTTTTCTGTAGCATTC	Always hairpin-free	central hole
**27[212]**	27[239]	-1-1	TTTAGAGATAACCCACAAGAAAGCGCAT	has 5′ TAGC and 3′ TFO hairpin in 258HP	
**28[279]**	25[271]	-1-2	AAAAATGAAAATAGCACCGTTTTT	has 5′ TAGC and 3′ TFO hairpin in 258HP	
**25[96]**	28[88]	-1-3	AAAGACACTAATAACGGAATACCC	has 5′ TAGC and 3′ TFO hairpin in 258HP	
**17[187]**	19[191]	-1-4	CAGGGATAGCAAGACCCTCAGAACCGCCAAGAGGGTT	has 5′ TAGC and 3′ TFO hairpin in 258HP	
**23[176]**	21[175]	-1-5	ACCACCGGCATTGACAGGAGGTTGGATACAGG	has 5′ TAGC and 3′ TFO hairpin in 258HP and 27HP (Fig 2)	
**25[59]**	28[55]	-1-6	ATACATACATAAATTAAGACTCCTTATTAC	has 5′ TAGC and 3′ TFO hairpin in 258HP	
**28[54]**	11[399]	-1-7	GCAGTATGTTTTTTTTTTTTTTTTTTTTTAAATAGCGAG AGGCTTTTGCAAAAGAATCGTTTAC	has 5′ TAGC and 3′ TFO hairpin in 258HP	links sectors
**21[123]**	23[127]	-1-8	TCTCTGAATTTACAAACAAATAAATCCTCGCCCCCTT	has 5′ TAGC and 3′ TFO hairpin in 258HP	
**23[208]**	21[207]	-1-9	CCTCAGAAGCCACCAGAACCACCAGGGGTCAG	has 5′ TAGC and 3′ TFO hairpin in 258HP	
**25[320]**	28[312]	-1-10	TATAGAAGATATTATTTATCCCAA	has 5′ TAGC and 3′ TFO hairpin in 258HP	
**21[160]**	23[159]	-1-11	CTTTTGATAGGCAGGTCAGACGATATCAAAAT	has 5′ TAGC and 3′ TFO hairpin in 258HP	
**17[272]**	33[186]	-1-12	AATTACCTGAGCAAAAGAATTTCATCAATATAATC	has 5′ TAGC and 3′ TFO hairpin in 258HP	
**25[288]**	28[280]	-1-13	TCATTACCTTTTTTGTTTAACGTC	has 5′ TAGC and 3′ TFO hairpin in 258HP	
**21[256]**	23[255]	-1-14	GTTATATAGTTTAGTATCATATGCGCTCAACA	has 5′ TAGC and 3′ TFO hairpin in 258HP	
**22[239]**	21[223]	-1-15	AGACTACCAAACAGTTAATGCCCCGTAACAGT	has 5′ TAGC and 3′ TFO hairpin in 258HP	
**25[352]**	28[344]	-1-16	GAGGCGTTAGCCTAATTTGCCAGT	has 5′ TAGC and 3′ TFO hairpin in 258HP	
**25[272]**	23[271]	-1-17	ATTTTCATAATCAATAATCGGCTGTAATTGAG	has 5′ TAGC and 3′ TFO hairpin in 258HP	
**23[352]**	25[351]	-1-18	ACAAAAGGACGCGCCTGTTTATCAAAGAACGC	has 5′ TAGC and 3′ TFO hairpin in 258HP	
**25[192]**	28[184]	-1-19	AGGGAGGGCAATGAAATAGCAATA	has 5′ TAGC and 3′ TFO hairpin in 258HP and 27HP (Fig 2)	
**25[384]**	28[376]	-1-20	AGGTTTTGAATCTTACCAACGCTA	has 5′ TAGC and 3′ TFO hairpin in 258HP	
**23[128]**	25[127]	-1-21	ATTAGCGTCCAATGAAACCATCGATTTGTCAC	has 5′ TAGC and 3′ TFO hairpin in 258HP	
**19[288]**	21[287]	-1-22	TTCTGTAATAGATTAAGACGCTGAGATGCAAA	has 5′ TAGC and 3′ TFO hairpin in 258HP	
**23[144]**	21[143]	-1-23	TTTTCATATGGCCTTGATATTCACCGTTCCAG	has 5′ TAGC and 3′ TFO hairpin in 258HP	
**25[176]**	23[175]	-1-24	GACATTCAGCAAAATCACCAGTAGCCAGAGCC	has 5′ TAGC and 3′ TFO hairpin in 258HP and 27HP (Fig 2)	
**16[223]**	15[239]	-1-25	TAACGATCCACAGACAGCCCTCATCAGTACCT	has 5′ TAGC and 3′ TFO hairpin in 258HP	
**21[176]**	19[175]	-1-26	AGTGTACTTGAAAGTATTAAGAGGCGGATAAG	has 5′ TAGC and 3′ TFO hairpin in 258HP and 27HP (Fig 2)	
**19[256]**	21[255]	-1-27	ATAAATCAATTTATCAAAATCATATAGGTTGG	has 5′ TAGC and 3′ TFO hairpin in 258HP	
**25[400]**	41[58]	-1-28	AATCAAGATTAGTTGCTATCGCTTAATGCGCCG	has 5′ TAGC and 3′ TFO hairpin in 258HP	
**19[240]**	20[224]	-1-29	TTAATGGAACAATTTCATTTGAATGGAGGTTT	has 5′ TAGC and 3′ TFO hairpin in 258HP	
**28[183]**	25[175]	-1-30	GCTATCTTACCGAAGCAAAAGGGC	has 5′ TAGC and 3′ TFO hairpin in 258HP and 27HP (Fig 2)	
**21[272]**	19[271]	-1-31	TAAATGCTGAAGAGTCAATAGTGAATATATGT	has 5′ TAGC and 3′ TFO hairpin in 258HP	
**19[155]**	21[159]	-1-32	GCTCAGTACCAGGCTGAGACTCCTCAAGAATACATGG	has 5′ TAGC and 3′ TFO hairpin in 258HP	
**18[239]**	17[223]	-1-33	CTTTGAATTTTCGTCACCAGTACAGTACCGTA	has 5′ TAGC and 3′ TFO hairpin in 258HP	
**25[208]**	23[207]	-1-34	TATTGACGCACCGACTTGAGCCATGCCGCCAC	has 5′ TAGC and 3′ TFO hairpin in 258HP	
**20[223]**	19[239]	-1-35	AGTACCGCTGTATCACCGTACTCATACCTTTT	has 5′ TAGC and 3′ TFO hairpin in 258HP	
**28[87]**	25[79]	-1-36	AAAAGAACTGGCATGAGGTGGCAA	has 5′ TAGC and 3′ TFO hairpin in 258HP	
**21[192]**	23[191]	-1-37	GTTTTAACCCAGAGCCGCCGCCAGAACCGCCT	has 5′ TAGC and 3′ TFO hairpin in 258HP and 27HP (Fig 2)	

**28[311]**	25[303]	-1-38	TCCAAATAAGAAACGAGCGCCCAA	has 5' TAGC and 3' TFO hairpin in 258HP	
**25[112]**	23[111]	-1-39	AAGTTTATTAGCAGCACCGTAATCGGCATTTT	has 5' TAGC and 3' TFO hairpin in 258HP	
**17[208]**	3[255]	-1-40	GAACCCATAACTACAACGCTTTTTTTTTTTTTTTTTTT TTGTATGGGATTTGAGAATAG	has 5' TAGC and 3' TFO hairpin in 258HP	links sectors
**26[239]**	25[223]	-1-41	GGTATTAAGGTGAATTATCACCGTGAAATTAT	has 5' TAGC and 3' TFO hairpin in 258HP	
**15[240]**	16[224]	-1-42	TTTACATCGATGAATATACAGTAAAGTTAGCG	has 5' TAGC and 3' TFO hairpin in 258HP	
**19[176]**	5[287]	-1-43	TGCCGTCGCCCTCAGAGCCTTTTTTTTTTTTTTTTTTT TTTTCACGTTGAGTTGCGCC	has 5' TAGC and 3' TFO hairpin in 258HP and 27HP (Fig 2)	links sectors
**28[375]**	25[367]	-1-44	ACGAGCGTCTTTCCAGTTAGCGAA	has 5' TAGC and 3' TFO hairpin in 258HP	
**25[144]**	23[143]	-1-45	ATATGGTTAAGGCCGGAAACGTCATTGCCATC	has 5' TAGC and 3' TFO hairpin in 258HP	
**21[320]**	23[319]	-1-46	TTTTCAAACCGTGTGATAAATAAGAGGCATTT	has 5' TAGC and 3' TFO hairpin in 258HP	
**25[336]**	23[335]	-1-47	GGTATTCTACAATAGATAAGTCCTGTAATAAG	has 5' TAGC and 3' TFO hairpin in 258HP	
**23[336]**	21[335]	-1-48	AGAATATATGGTTTGAAATACCGATATATTTT	has 5' TAGC and 3' TFO hairpin in 258HP	
**25[160]**	28[152]	-1-49	CCAAAGACCCTTTTTAAGAAAAGT	has 5' TAGC and 3' TFO hairpin in 258HP	
**30[231]**	30[212]	-1-50	TTGAGCGCTAATATCAGTTT	has 5' TAGC and 3' TFO hairpin in 258HP	
**23[272]**	21[271]	-1-51	AATCGCCATTACTAGAAAAAGCCTACTATATG	has 5' TAGC and 3' TFO hairpin in 258HP	
**23[91]**	25[95]	-1-52	GCGCGTTTTCATCAGTAGCGACAGAATCAAGAAACGC	has 5' TAGC and 3' TFO hairpin in 258HP	
**23[192]**	25[191]	-1-53	CCCTCAGATTGGGAATTAGAGCCAACCGATTG	has 5' TAGC and 3' TFO hairpin in 258HP and 27HP (Fig 2)	
**21[288]**	23[287]	-1-54	TCCAATCGAACACCGGAATCATAATATTTAAC	has 5' TAGC and 3' TFO hairpin in 258HP	
**23[256]**	25[255]	-1-55	GTAGGGCTTCTTTCCTTATCATTCATCGAGAA	has 5' TAGC and 3' TFO hairpin in 258HP	
**23[368]**	39[90]	-1-56	TTCTGTCCAGACGACGACACCTACATTTTGACG	has 5' TAGC and 3' TFO hairpin in 258HP	
**30[261]**	30[232]	-1-57	TTTAACACCCTGAACAAAGTCAGAGGGTAA	has 5' TAGC and 3' TFO hairpin in 258HP	
**23[320]**	25[319]	-1-58	TCGAGCCAGAACAAGAAAAATAATAAATCAGA	has 5' TAGC and 3' TFO hairpin in 258HP	
**23[240]**	24[224]	-1-59	AAGCCAACGTTATACAAATTCTTACACCACCC	has 5' TAGC and 3' TFO hairpin in 258HP	
**25[368]**	23[367]	-1-60	CCTCCCGAGTTCAGCTAATGCAGATAAAGTAA	has 5' TAGC and 3' TFO hairpin in 258HP	
**21[224]**	22[240]	-1-61	GCCCGTATTTTTTAACCTCCGGCTGGTCTGAG	has 5' TAGC and 3' TFO hairpin in 258HP	
**21[208]**	19[207]	-1-62	TGCCTTGACTGCCTATTTCGGAACTATAGCCC	has 5' TAGC and 3' TFO hairpin in 258HP	
**25[304]**	23[303]	-1-63	TAGCAAGCATCCCATCCTAATTTACATGTAAT	has 5' TAGC and 3' TFO hairpin in 258HP	
**28[223]**	25[207]	-1-64	AAGCCCAATAATAAGAGCAAGAAAAAGGTAAA	has 5' TAGC and 3' TFO hairpin in 258HP	
**25[224]**	26[240]	-1-65	TCATTAAAACCAAGTACCGCACTCCAAGAACG	has 5' TAGC and 3' TFO hairpin in 258HP	
**21[144]**	7[319]	-1-66	TAAGCGTCGAAGGATTAGGTTTTTTTTTTTTTTTTTTT TATATTCGGTCACTACGAA	has 5' TAGC and 3' TFO hairpin in 258HP	links sectors
**28[119]**	25[111]	-1-67	AACCGAGGAAACGCAACACGGAAT	has 5' TAGC and 3' TFO hairpin in 258HP	
**25[80]**	11[383]	-1-69	CATATAAAAGTTTGCCTTTTTTTTTTTTTTTTTTTTTA CAAAGCTGCTAACACTAT	has 5' TAGC and 3' TFO hairpin in 258HP	links sectors
**25[256]**	27[261]	-1-68	CAAGCAAGGCCTTTACAGAGAGAAAGAATTAACTGTTT	has 5' TAGC and 3' TFO hairpin in 258HP	
**19[208]**	17[207]	-1-70	GGAATAGGCACCCTCAGAACCGCCCCCAATAG	has 5' TAGC and 3' TFO hairpin in 258HP	
**19[304]**	35[154]	-1-71	TATTAATTAATTTTCCCTTTTTTACAAACAATTC	has 5' TAGC and 3' TFO hairpin in 258HP	
**23[160]**	25[159]	-1-72	CACCGGAACACCATTACCATTAGCTACCAGCG	has 5' TAGC and 3' TFO hairpin in 258HP	
**28[151]**	25[143]	-1-73	AAGCAGATAGCCGAACGAAAATTC	has 5' TAGC and 3' TFO hairpin in 258HP	
**21[336]**	37[122]	-1-74	AGTTAATTTCATCTTCTGAAGTGCCACGCTGAG	has 5' TAGC and 3' TFO hairpin in 258HP	
**23[288]**	25[287]	-1-75	AACGCCAACGAGCATGTAGAAACCCGTAGGAA	has 5' TAGC and 3' TFO hairpin in 258HP	
**19[272]**	17[271]	-1-76	GAGTGAATAAACATCAAGAAAACATTCATTTC	has 5' TAGC and 3' TFO hairpin in 258HP	
**17[256]**	19[255]	-1-77	GCGAATTAAAATTAATTACATTTAAACAGTAC	has 5' TAGC and 3' TFO hairpin in 258HP	
**24[223]**	23[239]	-1-78	TCAGAGCCCCGCCACCCTCAGAGCCCAGTATA	has 5' TAGC and 3' TFO hairpin in 258HP	
**27[240]**	28[224]	-1-79	TAGACGGGTAACATAAAAACAGGGATTGAGTT	has 5' TAGC and 3' TFO hairpin in 258HP	
**23[112]**	9[351]	-1-80	CGGTCATAATTAAAGCCAGTTTTTTTTTTTTTTTTTTA CTAAAACACTTAATCTTG	has 5' TAGC and 3' TFO hairpin in 258HP	links sectors
**28[343]**	25[335]	-1-81	TACAAAATAAACAGCCGCTTATCC	has 5' TAGC and 3' TFO hairpin in 258HP	
**23[304]**	21[303]	-1-82	TTAGGCAGGCGTTAAATAAGAATACAAGACAA	has 5' TAGC and 3' TFO hairpin in 258HP	

**25[128]**	28[120]	-1-83	AATCAATAAAAGTTACCAGAAGGA	has 5′ TAGC and 3′ TFO hairpin in 258HP	
**17[224]**	18[240]	-1-84	ACACTGAGACCAAGTTACAAAATCCCTGATTG	has 5′ TAGC and 3′ TFO hairpin in 258HP	
**21[304]**	19[303]	-1-85	AGAACGCGAAACATAGCGATAGCTATCGTCGC	has 5′ TAGC and 3′ TFO hairpin in 258HP	
**19[192]**	21[191]	-1-86	GATATAAGCTATTATTCTGAAACAGGTAATAA	has 5′ TAGC and 3′ TFO hairpin in 258HP and 27HP (Fig 2)	
**12[223]**	11[207]	-2-1	CGGAAGCAAACTCCAACAGGTCAGCAGGCAAG	has 5′ TAGC and 3′ TFO hairpin in 258HP	
**9[144]**	7[143]	-2-2	CTGATAAAGGAAGATTGTATAAGCTTAAATCA	has 5′ TAGC and 3′ TFO hairpin in 258HP	
**12[343]**	11[335]	-2-3	AATACTGCGGAATCGTTAATGCAG	has 5′ TAGC and 3′ TFO hairpin in 258HP	
**5[288]**	7[287]	-2-4	GACAATGAAGTTAAAGGCCGCTTTGTTTCCAT	has 5′ TAGC and 3′ TFO hairpin in 258HP	
**11[160]**	12[152]	-2-5	TTCTACTATAAGAGGTCATTTTTG	has 5′ TAGC and 3′ TFO hairpin in 258HP	
**11[80]**	41[383]	-2-6	ATTAGATAGTGTAGGTAAATTTTTTTTTTTTTTTTTTC CGGGGGTTTCGGTTTGCG	has 5′ TAGC and 3′ TFO hairpin in 258HP	links sectors
**12[151]**	11[143]	-2-7	CGGATGGCTTAGAGCTCGCGAGCT	has 5′ TAGC and 3′ TFO hairpin in 258HP	
**9[352]**	11[351]	-2-8	ACAAGAACCTTGCCCTGACGAGAACGCCAAAA	has 5′ TAGC and 3′ TFO hairpin in 258HP	
**12[54]**	41[399]	-2-9	AACAGTTGATTTTTTTTTTTTTTTTTTTGAGACGGGCA ACAGCTGATTGCCCTTCGCCAGGGT	has 5′ TAGC and 3′ TFO hairpin in 258HP	links sectors
**12[375]**	11[367]	-2-10	TAATAGTAAAATGTTTGAGGCATA	has 5′ TAGC and 3′ TFO hairpin in 258HP	
**3[256]**	5[255]	-2-11	AAAGGAACGGCTCCAAAAGGAGCCAGGTGAAT	has 5′ TAGC and 3′ TFO hairpin in 258HP	
**12[183]**	11[175]	-2-12	TAATTGCTCCTTTTGAATAGTAGT	has 5′ TAGC and 3′ TFO hairpin in 258HP and 27HP (Fig 2)	
**14[261]**	14[232]	-2-13	TTTAAAGATTAAGAGGAAGCCCGAAAGACT	has 5′ TAGC and 3′ TFO hairpin in 258HP	
**11[304]**	9[303]	-2-14	AGATTTAGTGGTTTAATTTCAACTGAACGGTG	has 5′ TAGC and 3′ TFO hairpin in 258HP	
**11[208]**	9[207]	-2-15	GCAAAGAATAAAGCTAAATCGGTTAGGTCATT	has 5′ TAGC and 3′ TFO hairpin in 258HP	
**7[304]**	5[303]	-2-16	GTAATGCCGCTGAGGCTTGCAGGGCAACAACC	has 5′ TAGC and 3′ TFO hairpin in 258HP	
**13[212]**	13[239]	-2-17	TTTATTCGAGCTTCAAAGCGAGTCAGAA	has 5′ TAGC and 3′ TFO hairpin in 258HP	
**5[176]**	35[287]	-2-18	TCGGCCTCAGGGCGATCGGTTTTTTTTTTTTTTTTTTT TAGAGACGCAGAGGCGGCCT	has 5′ TAGC and 3′ TFO hairpin in 258HP and 27HP (Fig 2)	links sectors
**5[304]**	19[154]	-2-19	ATCGCCCACGCATAACCGATATTAGCGGGGTTTT	has 5′ TAGC and 3′ TFO hairpin in 258HP	
**9[192]**	11[191]	-2-20	AGGCTATCGTACCAAAAACATTATCATCCAAT	has 5′ TAGC and 3′ TFO hairpin in 258HP and 27HP (Fig 2)	
**14[231]**	14[212]	-2-21	TCAAATATCGCGTTTTATTT	has 5′ TAGC and 3′ TFO hairpin in 258HP	
**9[91]**	11[95]	-2-22	AGAAAGGCCGGAGGCAATGCCTGAGTAATCATTTCGC	has 5′ TAGC and 3′ TFO hairpin in 258HP	
**11[288]**	12[280]	-2-23	AAAGATTCTTAAACAGTTCAGAAA	has 5′ TAGC and 3′ TFO hairpin in 258HP	
**11[384]**	12[376]	-2-24	CATAACCCGTTTTGCCAGAGGGGG	has 5′ TAGC and 3′ TFO hairpin in 258HP	
**7[176]**	5[175]	-2-25	AAATAATTGTAATGGGATAGGTCACGACAGTA	has 5′ TAGC and 3′ TFO hairpin in 258HP and 27HP (Fig 2)	
**11[224]**	10[240]	-2-26	ATTAAGCAGGAAGAAAAATCTACGACCAGTCA	has 5′ TAGC and 3′ TFO hairpin in 258HP	
**11[128]**	12[120]	-2-27	ATTTGGGGTAATTGCTGAATATAA	has 5′ TAGC and 3′ TFO hairpin in 258HP	
**2[239]**	3[223]	-2-28	AACTTTCAAGTTGGGTAACGCCAGTGCTGCAA	has 5′ TAGC and 3′ TFO hairpin in 258HP	
**3[272]**	17[186]	-2-29	GAATTGCGAATAATAATTTTTACCACCCTCATTTT	has 5′ TAGC and 3′ TFO hairpin in 258HP	
**9[128]**	11[127]	-2-30	TATTCAACAAAAATTTTTAGAACCATATTTTC	has 5′ TAGC and 3′ TFO hairpin in 258HP	
**9[320]**	11[319]	-2-31	AGGCTGGCTAAATTGGGCTTGAGAGAATACCA	has 5′ TAGC and 3′ TFO hairpin in 258HP	
**3[224]**	2[240]	-2-32	GGCGATTAACAGTTTCAGCGGAGTTGCTAAAC	has 5′ TAGC and 3′ TFO hairpin in 258HP	
**11[336]**	9[335]	-2-33	ATACATAAACACCAGAACGAGTAGTGACCTTC	has 5′ TAGC and 3′ TFO hairpin in 258HP	
**7[288]**	9[287]	-2-34	TAAACGGGGAGATTTGTATCATCGTTGAAAGA	has 5′ TAGC and 3′ TFO hairpin in 258HP	
**9[256]**	11[255]	-2-35	ATAAGGGAAGAACTGGCTCATTATTTAATAAA	has 5′ TAGC and 3′ TFO hairpin in 258HP	
**11[352]**	12[344]	-2-36	GGAATTACAGACTGGATAGCGTCC	has 5′ TAGC and 3′ TFO hairpin in 258HP	
**7[224]**	6[240]	-2-37	ATGTGAGCAGCAACGGCTACAGAGGCATCGGA	has 5′ TAGC and 3′ TFO hairpin in 258HP	
**9[160]**	11[159]	-2-38	GGAGAGGGTGCGGGAGAAGCCTTTGGCATCAA	has 5′ TAGC and 3′ TFO hairpin in 258HP	
**7[144]**	37[319]	-2-39	GCTCATTTCGCATCGTAACTTTTTTTTTTTTTTTTTTT GCCGTTCCGGCGCAACCAG	has 5′ TAGC and 3′ TFO hairpin in 258HP	links sectors

**0[239]**	1[223]	-2-40	TGTCGTCTGCCAAGCTTTCAGAGGTAAAACGA	has 5' TAGC and 3' TFO hairpin in 258HP	
**3[187]**	5[191]	-2-41	CTATTACGCCAGCTGCGCAACTGTTGGGAAGGAAGAT	has 5' TAGC and 3' TFO hairpin in 258HP	
**10[239]**	11[223]	-2-42	GGACGTTGATAAAGCCTCAGAGCATTAGCAAA	has 5' TAGC and 3' TFO hairpin in 258HP	
**5[256]**	7[255]	-2-43	TTCTTAAATCAGCAGCGAAAGACAGCTTTGAG	has 5' TAGC and 3' TFO hairpin in 258HP	
**7[192]**	9[191]	-2-44	GCCTTCCTTGTCAATCATATGTACATCTACAA	has 5' TAGC and 3' TFO hairpin in 258HP and 27HP (Fig 2)	
**11[144]**	9[143]	-2-45	GAAAAGGTATTTCAACGCAAGGATCGTTCTAG	has 5' TAGC and 3' TFO hairpin in 258HP	
**11[59]**	12[55]	-2-46	ACGAGTAGATTTAGAAGTTTCATTCCATAT	has 5' TAGC and 3' TFO hairpin in 258HP	
**7[320]**	9[319]	-2-47	GGCACCAACGATTATACCAAGCGCAGGCGCAT	has 5' TAGC and 3' TFO hairpin in 258HP	
**5[192]**	7[191]	-2-48	CGCACTCCAAACGGCGGATTGACCCGCGTCTG	has 5' TAGC and 3' TFO hairpin in 258HP and 27HP (Fig 2)	
**11[96]**	12[88]	-2-49	AAATGGTCGTTTTAAATATGCAAC	has 5' TAGC and 3' TFO hairpin in 258HP	
**11[112]**	9[111]	-2-50	GTTTAGCTCTCATATATTTTAAATACAGTCAA	has 5' TAGC and 3' TFO hairpin in 258HP	
**9[368]**	23[90]	-2-51	CATTACCCAAATCAACGTAAGCGTCAGACTGTA	has 5' TAGC and 3' TFO hairpin in 258HP	
**9[208]**	7[207]	-2-52	GCCTGAGAAATCGTAAAACTAGCAGTAGCCAG	has 5' TAGC and 3' TFO hairpin in 258HP	
**12[87]**	11[79]	-2-53	TAAAGTACGGTGTCTGGTTTGACC	has 5' TAGC and 3' TFO hairpin in 258HP	
**12[119]**	11[111]	-2-54	TGCTGTAGCTCAACATAATAACCT	has 5' TAGC and 3' TFO hairpin in 258HP	
**9[336]**	7[335]	-2-55	ATCAAGAGCATCTTTGACCCCCAGCCTAAAAC	has 5' TAGC and 3' TFO hairpin in 258HP	
**6[239]**	7[223]	-2-56	ACGAGGGTGAGTAACAACCCGTCGAACATTAA	has 5' TAGC and 3' TFO hairpin in 258HP	
**11[192]**	12[184]	-2-57	AAATCATAGATTAGAGAGTACCTT	has 5' TAGC and 3' TFO hairpin in 258HP and 27HP (Fig 2)	
**13[240]**	12[224]	-2-58	GCAAAGCGTTACCCTGACTATTATAACCAGAC	has 5' TAGC and 3' TFO hairpin in 258HP	
**12[311]**	11[303]	-2-59	ATCCCCCTCAAATGCTATCAGTTG	has 5' TAGC and 3' TFO hairpin in 258HP	
**11[400]**	25[58]	-2-60	CAGACGACGATAAAAACCAAGCAAACGTAGAAA	has 5' TAGC and 3' TFO hairpin in 258HP	
**11[320]**	12[312]	-2-61	CATTCAACCATAAATATTCATTGA	has 5' TAGC and 3' TFO hairpin in 258HP	
**4[223]**	5[239]	-2-62	CAAAGCGCCCGCTTCTGGTGCCGGTATCAGCT	has 5' TAGC and 3' TFO hairpin in 258HP	
**11[256]**	13[261]	-2-63	ACGAACTAATCAAAAATCAGGTCTGATTGCATCAATTT	has 5' TAGC and 3' TFO hairpin in 258HP	
**8[223]**	9[239]	-2-64	TGAACGGTGTCTGGAGCAAACAAGGGAACGAG	has 5' TAGC and 3' TFO hairpin in 258HP	
**11[176]**	9[175]	-2-65	AGCATTAAGACCCTGTAATACTTTTAGCTATT	has 5' TAGC and 3' TFO hairpin in 258HP and 27HP (Fig 2)	
**12[279]**	11[271]	-2-66	ACGAGAATGACCATAAACGGAACA	has 5' TAGC and 3' TFO hairpin in 258HP	
**11[368]**	9[367]	-2-67	GTAAGAGCCATTCAGTGAATAAGGCGGATATT	has 5' TAGC and 3' TFO hairpin in 258HP	
**5[155]**	7[159]	-2-68	TTTGAGGGGACGACGTTGGTGTAGATGGGTTTAACCA	has 5' TAGC and 3' TFO hairpin in 258HP	
**9[304]**	7[303]	-2-69	TACAGACCGAAACAAAGTACAACGTAAAATAC	has 5' TAGC and 3' TFO hairpin in 258HP	
**5[240]**	4[224]	-2-70	TGCTTTCGTTTAATTGTATCGGTTAAACCAGG	has 5' TAGC and 3' TFO hairpin in 258HP	
**9[288]**	11[287]	-2-71	GGACAGATTTAATCATTGTGAATTACAGGTAG	has 5' TAGC and 3' TFO hairpin in 258HP	
**7[272]**	5[271]	-2-72	ATGAGGAATGCGGGATCGTCACCCCAGCTTGA	has 5' TAGC and 3' TFO hairpin in 258HP	
**9[112]**	39[351]	-2-73	ATCACCATAACGTTAATATTTTTTTTTTTTTTTTTTTT TGCAGGCGCTTGGTGCTG	has 5' TAGC and 3' TFO hairpin in 258HP	links sectors
**3[208]**	33[255]	-2-74	GGGGGATGGGTTTTCCCAGTTTTTTTTTTTTTTTTTTT TTCGAAACGTACAAGATAGAC	has 5' TAGC and 3' TFO hairpin in 258HP	links sectors
**1[224]**	0[240]	-2-75	CGGCCAGTTTCCAGACGTTAGTAATAAAGTTT	has 5' TAGC and 3' TFO hairpin in 258HP	
**7[160]**	9[159]	-2-76	ATAGGAACAAAAGCCCCAAAAACATTAATGCC	has 5' TAGC and 3' TFO hairpin in 258HP	
**9[240]**	8[224]	-2-77	GCGCAGACTCCATGTTACTTAGCCAGAATCGA	has 5' TAGC and 3' TFO hairpin in 258HP	
**7[208]**	5[207]	-2-78	CTTTCATCGATTCTCCGTGGGAACAGCCAGCT	has 5' TAGC and 3' TFO hairpin in 258HP	
**5[272]**	3[271]	-2-79	TACCGATAAAATCTCCAAAAAAAAAACTAAAG	has 5' TAGC and 3' TFO hairpin in 258HP	
**7[123]**	9[127]	-2-80	CATTAAATTTTTGAAATATTTAAATTGTACAATATGA	has 5' TAGC and 3' TFO hairpin in 258HP	
**11[272]**	9[271]	-2-81	ACATTATTACCTTATGCGATTTTAACCGAACT	has 5' TAGC and 3' TFO hairpin in 258HP	
**7[336]**	21[122]	-2-82	GAAAGAGGCAAAAGAATACAATGGAAAGCGCAG	has 5' TAGC and 3' TFO hairpin in 258HP	
**5[208]**	3[207]	-2-83	TTCCGGCACATTCGCCATTCAGGCTGGCGAAA	has 5' TAGC and 3' TFO hairpin in 258HP	
**9[272]**	7[271]	-2-84	GACCAACTCCTGATAAATTGTGTCACTTTTTC	has 5' TAGC and 3' TFO hairpin in 258HP	

**7[256]**	9[255]	-2-85	GACTAAAGGAAATCCGCGACCTGCGGTCAATC	has 5' TAGC and 3' TFO hairpin in 258HP	
**9[176]**	7[175]	-2-86	TTTGAGAGCCCGGTTGATAATCAGGCCATCAA	has 5' TAGC and 3' TFO hairpin in 258HP and 27HP (Fig 2)	
**37[192]**	39[191]	-3-1	TCTGGTCAGCCCTAAAACATCGCCCTTCTGAC	has 5' TAGC and 3' TFO hairpin in 258HP and 27HP (Fig 2)	
**44[343]**	41[335]	-3-2	CCCAGCAGGCGAAAATCTGTCGTG	has 5' TAGC and 3' TFO hairpin in 258HP	
**39[208]**	37[207]	-3-3	ACGTGGCAAATGCGCGAACTGATAGTTGGCAA	has 5' TAGC and 3' TFO hairpin in 258HP	
**42[239]**	41[223]	-3-4	ACATACGAGTCCATCACGCAAATTCCGAGTAA	has 5' TAGC and 3' TFO hairpin in 258HP	
**41[160]**	44[152]	-3-5	TAGACAGGTTAGAGCTTGACGGGG	has 5' TAGC and 3' TFO hairpin in 258HP	
**39[352]**	41[351]	-3-6	CGGCCAGATGTTCTTCGCGTCCGTATTAATGA	has 5' TAGC and 3' TFO hairpin in 258HP	
**41[59]**	44[55]	-3-7	CTACAGGGCGCGTCGCTGCGCGTAACCACC	has 5' TAGC and 3' TFO hairpin in 258HP	
**39[336]**	37[335]	-3-8	GCACTCTGTTCGCACTCAATCCGCTGGAGGTG	has 5' TAGC and 3' TFO hairpin in 258HP	
**35[288]**	37[287]	-3-9	TTAGTGATTTCGTCTCGTCGCTGGCAACGGCA	has 5' TAGC and 3' TFO hairpin in 258HP	
**37[320]**	39[319]	-3-10	CTTACGGCCGGGCGCGGTTGCGGTAGCGCAGT	has 5' TAGC and 3' TFO hairpin in 258HP	
**37[144]**	21[319]	-3-11	CTAAAGCAAAGTATTAGACTTTTTTTTTTTTTTTTTTT AGAATCCTTGAAGAAAACT	has 5' TAGC and 3' TFO hairpin in 258HP	links sectors
**39[160]**	41[159]	-3-12	ATTCTGGCAGTAGAAGAACTCAAAAGGGATTT	has 5' TAGC and 3' TFO hairpin in 258HP	
**36[223]**	35[239]	-3-13	CCAGAAGGTCATTTTGCGGAACAAGCGGTTGT	has 5' TAGC and 3' TFO hairpin in 258HP	
**39[240]**	40[224]	-3-14	AAATCGTTATCCCTTACACTGGTGATGGCTAT	has 5' TAGC and 3' TFO hairpin in 258HP	
**41[176]**	39[175]	-3-15	GCCAGAATTAACATCACTTGCCTGCAACAGAG	Reporter staple 3 (Fig 3). Has 5' TAGC and 3' TFO hairpin in 258HP and 27HP (Fig 2). Has 5' TAGC and 3' TFO hairpin in 3HP origami Extension-free in 1HP and 2HP origami (Fig 3).	
**39[288]**	41[287]	-3-16	GGTGCCCCATCATGGTCATAGCTGCTCACATT	has 5' TAGC and 3' TFO hairpin in 258HP	
**44[311]**	41[303]	-3-17	TCCGAAATCGGCAAAATGCGCTCA	has 5' TAGC and 3' TFO hairpin in 258HP	
**37[192]**	41[191]	-3-18	CTGAAAGCCTTCTTTGATTAGTAACCTGAGAA	has 5' TAGC and 3' TFO hairpin in 258HP and 27HP (Fig 2)	
**37[208]**	35[207]	-3-19	ATCAACAGTCTTTAGGAGCACTAAAGTTTGAG	has 5' TAGC and 3' TFO hairpin in 258HP	
**41[400]**	11[58]	-3-20	GGTTTTTCTTTTCACCAGTTCCCAATTCTGCGA	has 5' TAGC and 3' TFO hairpin in 258HP	
**33[224]**	34[240]	-3-21	TTAGAACCTCCCGGAATTTGTGAGGCGCCATG	has 5' TAGC and 3' TFO hairpin in 258HP	
**31[240]**	32[224]	-3-22	CCTCACCGTGGAGCCGCCACGGGATTCAGGTT	has 5' TAGC and 3' TFO hairpin in 258HP	
**34[239]**	33[223]	-3-23	TTTACCAGTACCATATCAAAATTATGGAAGGG	has 5' TAGC and 3' TFO hairpin in 258HP	
**38[239]**	37[223]	-3-24	GGACTTGTAAGGTTATCTAAAATATTGAAAGG	has 5' TAGC and 3' TFO hairpin in 258HP	
**41[336]**	39[335]	-3-25	CCAGCTGCGAGCCTCCTCACAGTTGCGCCTGT	has 5' TAGC and 3' TFO hairpin in 258HP	
**41[80]**	25[383]	-3-26	TGCTTTGATCATGGAAATATTTTTTTTTTTTTTTTTTA TAAACAACATCTTGCGGG	has 5' TAGC and 3' TFO hairpin in 258HP	links sectors
**35[208]**	33[207]	-3-27	TAACATTAAGCGGAATTATCATCATTATACTT	has 5' TAGC and 3' TFO hairpin in 258HP	
**41[368]**	39[367]	-3-28	GGAGAGGCTGCCAGCACGCGTGCCATGCGGCG	has 5' TAGC and 3' TFO hairpin in 258HP	
**41[384]**	44[376]	-3-29	TATTGGGCACCGCCTGGCCCTGAG	has 5' TAGC and 3' TFO hairpin in 258HP	
**41[304]**	39[303]	-3-30	CTGCCCGCCGAGCTCGAATTCGTACTGCATCA	has 5' TAGC and 3' TFO hairpin in 258HP	
**44[87]**	41[79]	-3-31	GCAAGTGTAGCGGTCAACTATGGT	has 5' TAGC and 3' TFO hairpin in 258HP	
**41[256]**	43[261]	-3-32	TGGGGTGCGGGTTGAGTGTTGTTCGTGGACTCCAATTT	has 5' TAGC and 3' TFO hairpin in 258HP	
**44[151]**	41[143]	-3-33	AAAGCCGGCGAACGTGACAGGAGG	has 5' TAGC and 3' TFO hairpin in 258HP	
**41[192]**	44[184]	-3-34	GTGTTTTTACTAAATCGGAACCCT	has 5' TAGC and 3' TFO hairpin in 258HP and 27HP (Fig 2)	
**39[304]**	37[303]	-3-35	GACGATCCATGAGCCGGGTCACTGGGTGGTGC	has 5' TAGC and 3' TFO hairpin in 258HP	
**41[272]**	39[271]	-3-36	TGAGCTAATTTCCTGTGTGAAATTGGGTTACC	has 5' TAGC and 3' TFO hairpin in 258HP	
**43[212]**	43[239]	-3-37	TTTGTGAACCATCACCCAAATCCACTAT	has 5' TAGC and 3' TFO hairpin in 258HP	
**44[375]**	41[367]	-3-38	AGAGTTGCAGCAAGCGACGCGCGG	has 5' TAGC and 3' TFO hairpin in 258HP	
**39[256]**	41[255]	-3-39	CAGATGCCGTTATCCGCTCACAATGTAAAGCC	has 5' TAGC and 3' TFO hairpin in 258HP	
**37[224]**	38[240]	-3-40	AATTGAGGAGAACGTCAGCGTGGTAACGTGCC	has 5' TAGC and 3' TFO hairpin in 258HP	

**41[96]**	44[88]	-3-41	TATAACGTGGGCGCTAGGGCGCTG	has 5' TAGC and 3' TFO hairpin in 258HP	
**33[272]**	3[186]	-3-42	GGATAGCTCTCACGGAAAATTTGCGGGCCTCTTCG	has 5' TAGC and 3' TFO hairpin in 258HP	
**44[279]**	41[271]	-3-43	AGAATAGCCCGAGATACTAATGAG	has 5' TAGC and 3' TFO hairpin in 258HP	
**39[91]**	41[95]	-3-44	CTCAATCGTCTGAGCAACAGGAAAAACGCCGAGCACG	has 5' TAGC and 3' TFO hairpin in 258HP	
**35[240]**	36[224]	-3-45	GTACATCGTGCCGCCAGCAGTTGGAGAAACCA	has 5' TAGC and 3' TFO hairpin in 258HP	
**43[240]**	44[224]	-3-46	TAAAGAACCAGTTTGGAACAAGAGTCAAGTTT	has 5' TAGC and 3' TFO hairpin in 258HP	
**37[336]**	7[122]	-3-47	TCCAGCATCAGCGGGGTCATTTGTTAAAATTCG	has 5' TAGC and 3' TFO hairpin in 258HP	
**44[54]**	25[399]	-3-48	ACACCCGCCGTTTTTTTTTTTTTTTTTTTTTGCACCCA GCTACAATTTTATCCTGAAGCCTTA	has 5' TAGC and 3' TFO hairpin in 258HP	links sectors
**35[192]**	37[191]	-3-49	ATTTTAAACAACTAATAGATTAGAAATCAATA	has 5' TAGC and 3' TFO hairpin in 258HP and 27HP (Fig 2)	
**39[320]**	41[319]	-3-50	GTCACTGCGAGGATCCCCGGGTACTTTCCAGT	has 5' TAGC and 3' TFO hairpin in 258HP	
**35[176]**	19[287]	-3-51	TGCCCGAAATGATGGCAATTTTTTTTTTTTTTTTTTTT TGATGATGAAACAACCTTGC	Reporter staple 0 (Fig 3). Has 5' TAGC and 3' TFO hairpin in 258HP and 27HP (Fig 2). Has 5' TAGC and no 3' TFO hairpin to form 1HP minimal junction with reporter Cy5-labeled Staple 1 (3-84) (Fig 3). Has 5' TAGC and no 3' TFO hairpin in 1, 2, 3 HP origami (Fig 3).	links sectors
**35[272]**	33[271]	-3-52	GCCGCACAAACAGCGGATCAAACTTGGTGAAG	has 5' TAGC and 3' TFO hairpin in 258HP	
**46[231]**	46[212]	-3-53	GGGCGATGGCCCACTACTTT	has 5' TAGC and 3' TFO hairpin in 258HP	
**37[304]**	35[303]	-3-54	CATCCCACAAACGCGGTCCGTTTTGAAGGGTA	has 5' TAGC and 3' TFO hairpin in 258HP	
**37[256]**	39[255]	-3-55	GGTCAGCATTTGCTCGTCATAAACAACGGCAT	has 5' TAGC and 3' TFO hairpin in 258HP	
**37[160]**	39[159]	-3-56	CTGAACCTGAACCACCAGCAGAAGAAAGGGAC	has 5' TAGC and 3' TFO hairpin in 258HP	
**39[176]**	37[175]	-3-57	ATAGAACCATTAAAAATACCGAACCAAATATC	Reporter staple 2. Has 5' TAGC and 3' TFO hairpin in 258HP and 27HP (Fig 2). Has 5' TAGC and 3' TFO hairpin in 2HP and 3HP origami. Extension-free in 1HP origami.	
**46[261]**	46[232]	-3-58	TTTCGTCAAAGGGCGAAAAACCGTCTATCA	has 5' TAGC and 3' TFO hairpin in 258HP	
**37[288]**	39[287]	-3-59	GCACCGTCTTGCCCTGCGGCTGGTGCGGTGCC	has 5' TAGC and 3' TFO hairpin in 258HP	
**41[128]**	44[120]	-3-60	GGAGCTAAGCGAGAAAGGAAGGGA	has 5' TAGC and 3' TFO hairpin in 258HP	
**41[320]**	44[312]	-3-61	CGGGAAACCCTGTTTGATGGTGGT	has 5' TAGC and 3' TFO hairpin in 258HP	
**41[144]**	39[143]	-3-62	CCGATTAACTATCGGCCTTGCTGGCACACGAC	has 5' TAGC and 3' TFO hairpin in 258HP	
**41[224]**	42[240]	-3-63	AAGAGTCTGCCGGAAGCATAAAGTTCCACACA	has 5' TAGC and 3' TFO hairpin in 258HP	
**33[187]**	35[191]	-3-64	CTGATTGTTTGGATATTCCTGATTATCAGCGTTATTA	has 5' TAGC and 3' TFO hairpin in 258HP	
**39[128]**	41[127]	-3-65	TCACCAGTTAATATCCAGAACAATTCAGAGCG	has 5' TAGC and 3' TFO hairpin in 258HP	
**41[352]**	44[344]	-3-66	ATCGGCCAGTCCACGCTGGTTTGC	has 5' TAGC and 3' TFO hairpin in 258HP	
**35[304]**	5[154]	-3-67	AAGTTAAACGATGCTGATTTCGTGCATCTGCCAG	has 5' TAGC and 3' TFO hairpin in 258HP	
**40[223]**	39[239]	-3-68	TAGTCTTTCAGACAATATTTTTGATGTTCAGC	has 5' TAGC and 3' TFO hairpin in 258HP	
**39[368]**	9[90]	-3-69	GGCCGTTTTCACGGTCATAGATTCAAAAGGGTG	has 5' TAGC and 3' TFO hairpin in 258HP	
**32[223]**	31[239]	-3-70	TAACGTCAAGAAATTGCGTAGATTACGGATAA	has 5' TAGC and 3' TFO hairpin in 258HP	
**35[155]**	37[159]	-3-71	GACAACTCGTATTACATTTGAGGATTTAGTCACCTTG	has 5' TAGC and 3' TFO hairpin in 258HP	
**35[256]**	37[255]	-3-72	AAATCCCGCACATCCTCATAACGGGCTGGTCT	has 5' TAGC and 3' TFO hairpin in 258HP	
**33[208]**	17[255]	-3-73	CTGAATAATTTGCACGTAATTTTTTTTTTTTTTTTTTT TTTAACGGATTCGGCGCAGAG	has 5' TAGC and 3' TFO hairpin in 258HP	links sectors
**44[183]**	41[175]	-3-74	AAAGGGAGCCCCCGATAACGGTAC	has 5' TAGC and 3' TFO hairpin in 258HP and 27HP (Fig 2)	
**37[123]**	39[127]	-3-75	AGCCAGCAGCAAAGCGGTCAGTATTAACATGGCAGAT	has 5' TAGC and 3' TFO hairpin in 258HP	
**44[119]**	41[111]	-3-76	AGAAAGCGAAAGGAGCGCTTTCCT	has 5' TAGC and 3' TFO hairpin in 258HP	
**33[256]**	35[255]	-3-77	TTTCTCCGTAAATTTCTGCTCATTACATAAAA	has 5' TAGC and 3' TFO hairpin in 258HP	

**39[272]**	37[271]	-3-78	TGCAGCCAAATGGGTAAAGGTTTCGCAACCGC	has 5′ TAGC and 3′ TFO hairpin in 258HP	
**41[288]**	44[280]	-3-79	AATTGCGTTCCCTTATAAATCAAA	has 5′ TAGC and 3′ TFO hairpin in 258HP	
**41[208]**	39[207]	-3-80	TGAGGCCAAACCGTTGTAGCAATAGTAAGAAT	has 5′ TAGC and 3′ TFO hairpin in 258HP	
**39[144]**	37[143]	-3-81	CAGTAATAATAAAACAGAGGTGAGTGAAAAAT	has 5′ TAGC and 3′ TFO hairpin in 258HP	
**37[272]**	35[271]	-3-82	AAGAATGCCAGCCTCCGGCCAGAGTAAAAAAA	has 5′ TAGC and 3′ TFO hairpin in 258HP	
**41[112]**	39[111]	-3-83	CGTTAGAAATTACCGCCAGCCATTAATGGATT	has 5′ TAGC and 3′ TFO hairpin in 258HP	
**37[176]**	35[175]	-3-84	AAACCCTCGCCGTCAATAGATA**AT**AAATCCTT	Reporter staple 1. Cy5-labeled reporter staple in 1, 2, 3, 27, and 258HP origami and in minimal junction HP1. In these constructs, it has 5′ TAGC and 3′ TFO hairpin. Position of internal Cy5 label (IDT "iCy5" modification) is between A and T in the bold underlined sequence.	
**44[223]**	41[207]	-3-85	TTTGGGGTCGAGGTGCCGTAAAGCATAATCAG	has 5′ TAGC and 3′ TFO hairpin in 258HP	
**39[112]**	23[351]	-3-86	ATTTACATCCGCCTGCAACTTTTTTTTTTTTTTTTTTTCCTAAATTTAAAAGTACCG	has 5′ TAGC and 3′ TFO hairpin in 258HP	links sectors

4. Structures of triplex-forming oligonucleotides (TFOs).

The sequence of unmodified TFO (Fig 2B) was 5′-TTCTTTCTTCTCT-3′ (IDT).

The sequence of TFO modified with Alexa Fluor 647 at the 3′ end (TFO-A647) was 5′-TTCTTTCTTCTCT-3′-Alexa647 (IDT).

The sequence of base modified TFO (TFO-Z) was 5′-TTZTTTZTTZTZT-3′ where Z is 6-amino-5-nitropyridine-2-one (Biotage, Sweden).

The sequence of the base modified 5′ psoralen- and 3′ biotin-modified TFO (pso-TFO-Z) was 5′-psoralen-C_6-TTZTTTZTTZTZT-C_6-biotin-3′ where Z is 6-amino-5-nitropyridine-2-one (Biotage, Sweden).

The sequence of 5′ psoralen- and 3′ amine-modified TFO (pso-TFO-C6-NH2) was 5′-Psoralen-C_6-TTCTTTCTTCTCT-C_6-NH_2-3′ (Fidelity Oligos, Gaithersburg, USA). This oligo was also used as precursor for in-house synthesis of pso-TFO-PEG5K and pso-TFO-PEG20K, and as precursor for synthesis of pso-TFO-biotin.

The sequence of 5′ psoralen- and 3′ biotin-modified TFO (pso-TFO-biotin) was 5′-psoralen-C_6-TTCTTTCTTCTCT-C_6-PEO_4-biotin-3′ (Fidelity Oligos, Gaithersburg, USA).

The sequence of 5′ psoralen- and 3′-PEG4-NH2-modified TFO was 5′-psoralen-C6-TTCTTTCTTCTCTAACU*T (Fidelity oligos, Gaithersburg. USA), where U* is

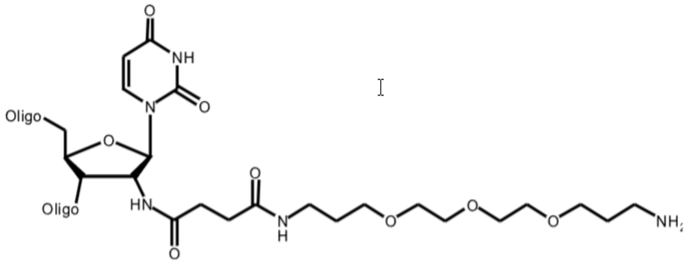

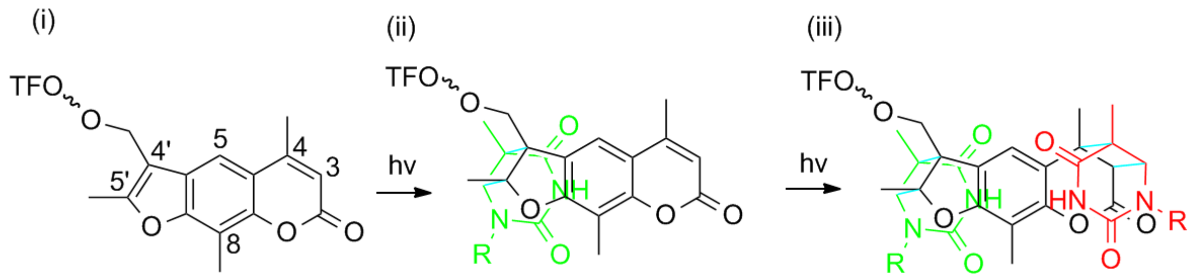

Figure S1. TFO-directed psoralen cross-linking. 4,5′,8-trimethyl psoralen (i) can be attached to a triplex-forming oligonucleotide (TFO) in such a way as to allow the 3,4 and 4′,5′-double bonds to react with one thymine on each strand at a 5′-TpA sequence located at the duplex-triplex junction. Upon intercalation the absorption of a single UV photon of wavelength >310 nm (typically around 365 nm) leads to the generation of a monoadduct *via* a cycloaddition reaction at one of these bonds, with the 4′,5′-double bond most susceptible to mono-adduct formation (ii). The subsequent absorption of a second photon leads to a second cycloaddition reaction at the 3,4 double bond, and the formation of a bis-adduct, with the psoralen-TFO cross-linked to both DNA strands (iii). The psoralen molecule is shown in black and the two T's within the 5′-TpA step in green and red. In this study the two T's stem from proximal staple strands, mono-adduct formation will attach the TFO to the one staple, whilst bis-adduct formation will crosslink the TFO and the two staple strands together.

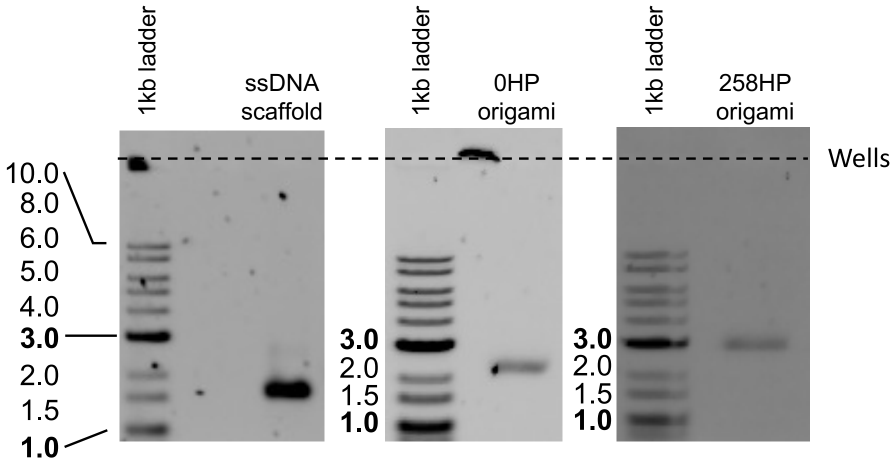

Figure S2. **Relative mobilities of folded and purified 0HP and 258HP origami during agarose gel electrophoresis at pH 4.8**. 0HP and 258 HP origami were folded in OB-8.0 using 50 nM 8064 ssDNA scaffold and a 150 nM extension-free (0HP) and extension-containing (258HP) staples. Both nanostructures were purified by ultracentrifugation in a 10-50% glycerol gradient in OB-4.8, and the peak fractions were run alongside 8064 ssDNA scaffold and dsDNA length markers on 1% agarose gels in a pH 4.8 running buffer at room temperature. Bands were visualised by scanning for ethidium bromide fluorescence.

Commentary: Bands for origami ran with the same mobilities (compared to the linear marker) as the furthest migrating origami bands in the un-purified co-fold experiments (*e.g.,* Fig 2B, Fig S6, S7, S12), as well as other experiments in the manuscript (*e.g.,* Fig 4, 5, 6).

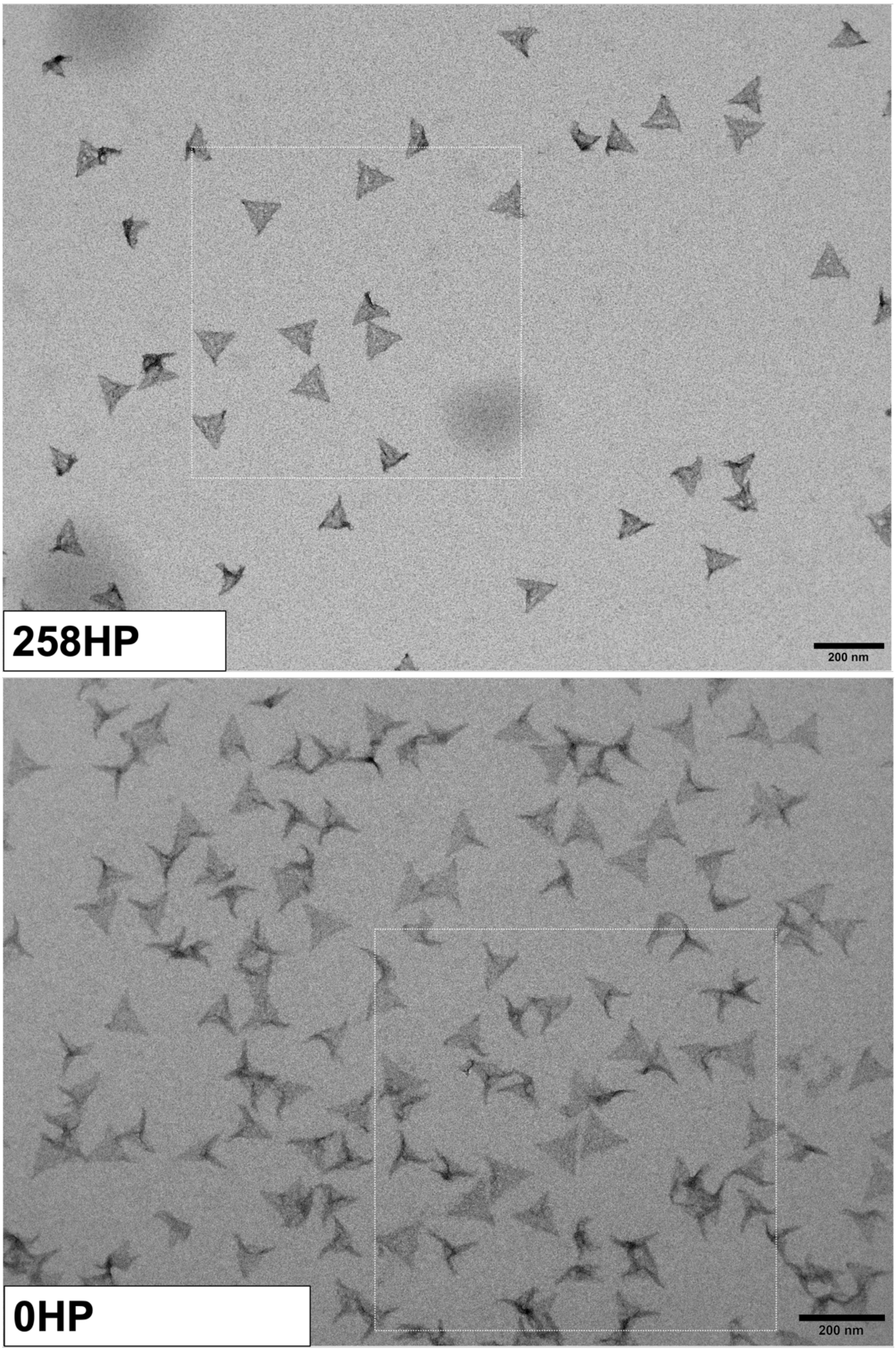

Figure S3. Representative TEM images of purified 258HP (top image) and 0HP (bottom image) origami triangles. The dashed box indicates the cut-out region used in Fig 2A in the main text.

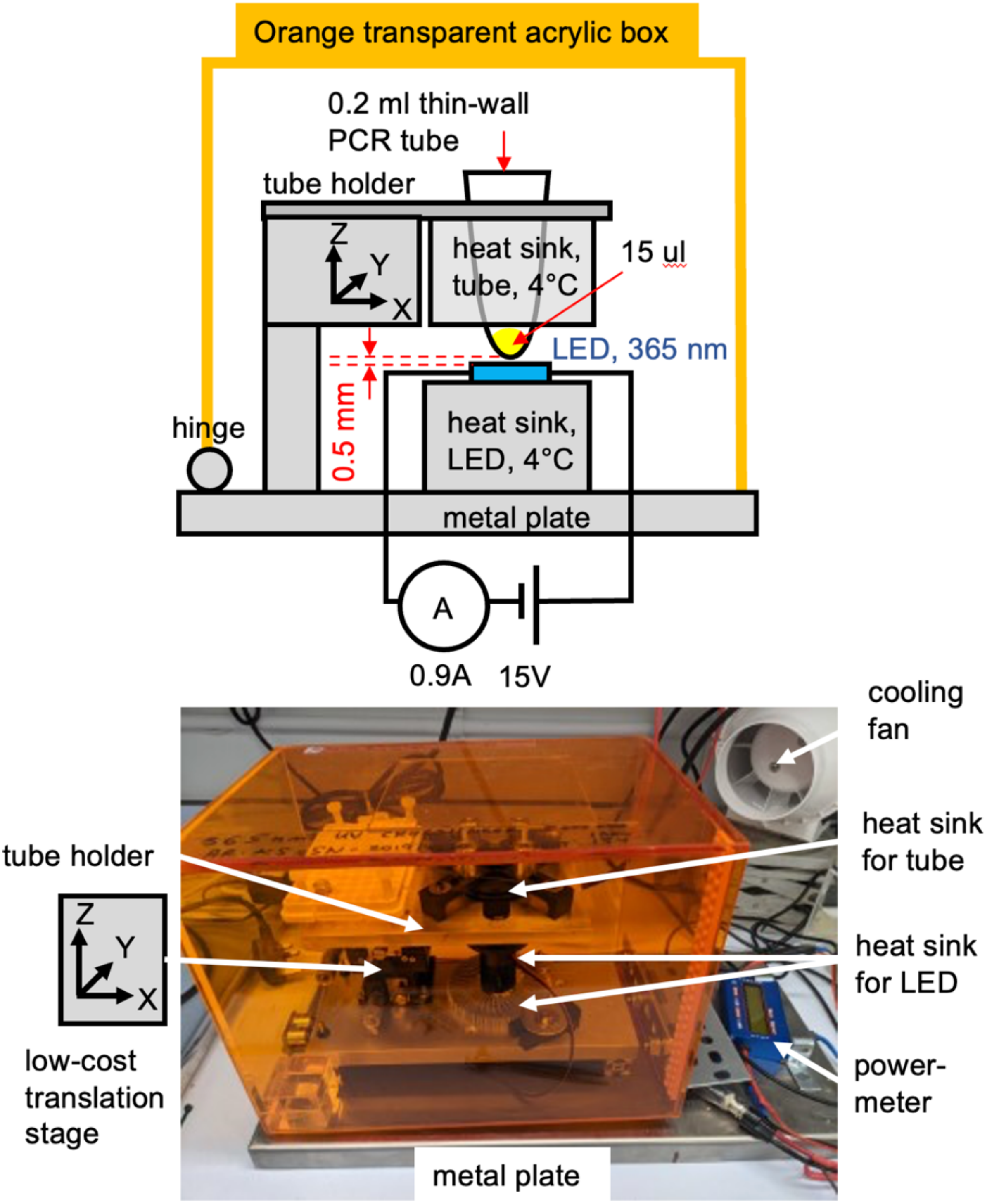

Figure S4. Schematic (top) and picture (bottom) of a home-built UVA photo-crosslinking setup based on a 2000→3800 mW 365 nm LED chip.

Co-folding at pH 8.0 (unpurified)

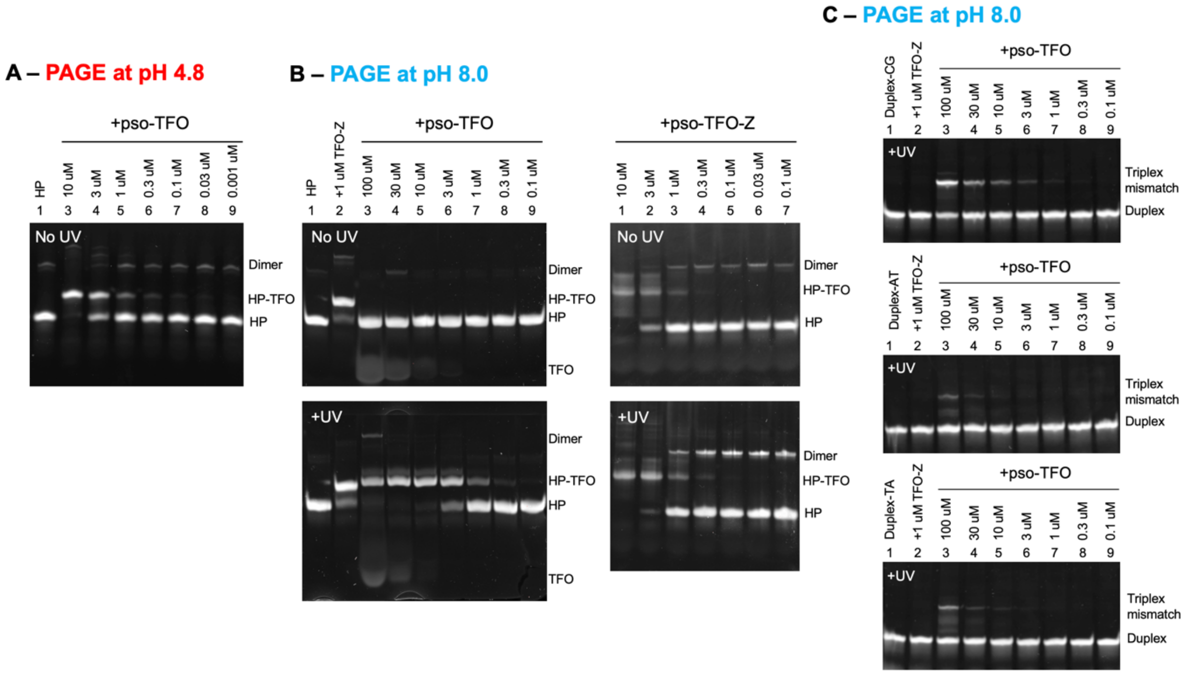

Figure S5. Targeting of individual hairpins by TFOs at different pH. All complexes were annealed in OB-8.0 and then separated on a 15% non-denaturing polyacrylamide gel in a running buffer at either pH 4.8 (A) or pH 8.0 (B+C). The final concentration of duplex was 1 uM and the final concentration of TFO varied between 1 and 100 uM as indicated. Samples in the gels labelled with +UV were irradiated with 365 nm light for 10 seconds to crosslink the complexes. Gels were post stained with GelRed and imaged using a GelDoc imager. The position of the expected HP and HP-TFO complexes are shown using the labels on the right of each gel and the position of a minor band representing a potential HP dimer is also indicated.

Commentary: As a control, each gel contains a sample generated with a triplex containing a TFO synthesised with a nucleobase analogue ("Z") that works at high pH values (TFO-Z, lane 2). Unlike the unmodified pso-TFO, these complexes remained stable when run on a pH 8.0 gel in the absence of crosslinking and confirms the identity of the crosslinked triplex bands. Under these experimental conditions the equivalent psoralen-modified TFO (pso-TFO-Z) bearing a 3′- biotin crosslinked DNA with a similar efficiency to the unmodified pso-TFO (bottom panel B). The duplex used in panel A and B contained the intended target sequence for the TFO, whilst the three duplexes used in panel C generated triplexes containing a mismatched C-CG (top), C-AT (middle), and C-TA (bottom) triplet. Only minor crosslinking is evident with these sequences compared to full crosslinking with the intended target duplex (compare with bottom panel B at same concentrations).

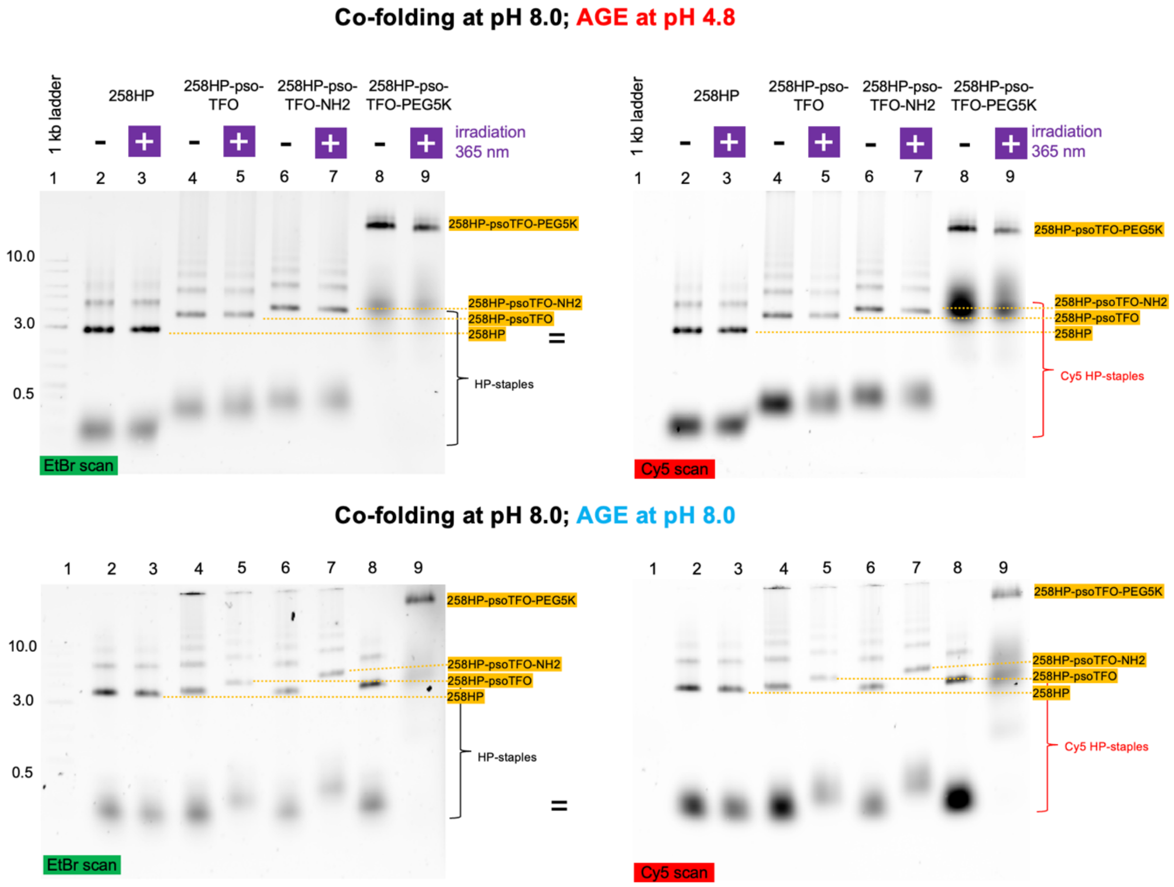

Fig S6. Targeting of origami hairpins by TFOs bearing additional moieties (EtBr and Cy5 scans). AGE analysis of the unpurified co-folded mixture of 258HP origami loaded with pso-TFOs bearing different 3′-modifications. Origamis were first prepared in OB-8.0 buffer by annealing 50 nM scaffold with 150 nM staples in the absence, or the presence of 100 uM pso-TFO. The samples were then irradiated with 365 nm light for 10 seconds and analysed by AGE at pH 4.8 (left gel, annotated in red) and pH 8.0 (right gel, annotated in blue). The positions of the expected HP and HP-TFO complexes are shown using a red asterisk, whilst the positions of the origami and origami-TFO complexes are shown using a black asterisk. The bands located above the origami band in each lane are due to misfolded aggregates (expected to form during origami folding[7]) and are removed by ultracentrifugation during purification. The gels were scanned for EtBr fluorescence and the Cy5 reporter strand.

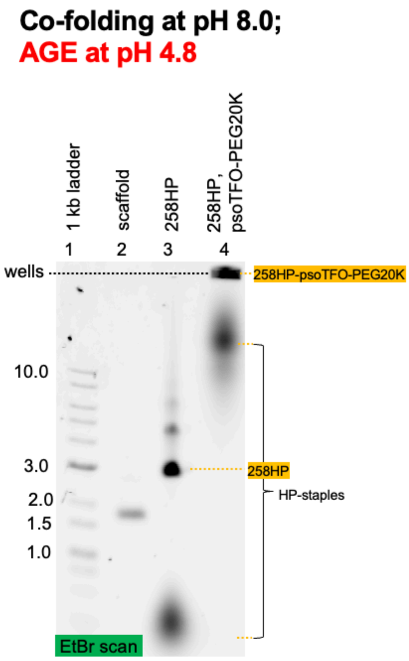

Fig S7. Functionalization of origami with pso-TFO carrying a bulky 3′ modification. 258HP-pso-TFO-PEG20K complexes were folded in OB-8.0 and the folded mixtures run on a 1% agarose gel in a pH 4.8 running buffer. The complexes were run for 5 hours alongside 258HP (TFO-free) origami, ssDNA scaffold and dsDNA length markers. Electrophoresis bands were visualised by scanning for ethidium bromide fluorescence.

Commentary: The mobility of the 258HP-psoTFO-PEG20K complex was severely retarded due to the attachment of multiple PEG20K moieties to the structure.

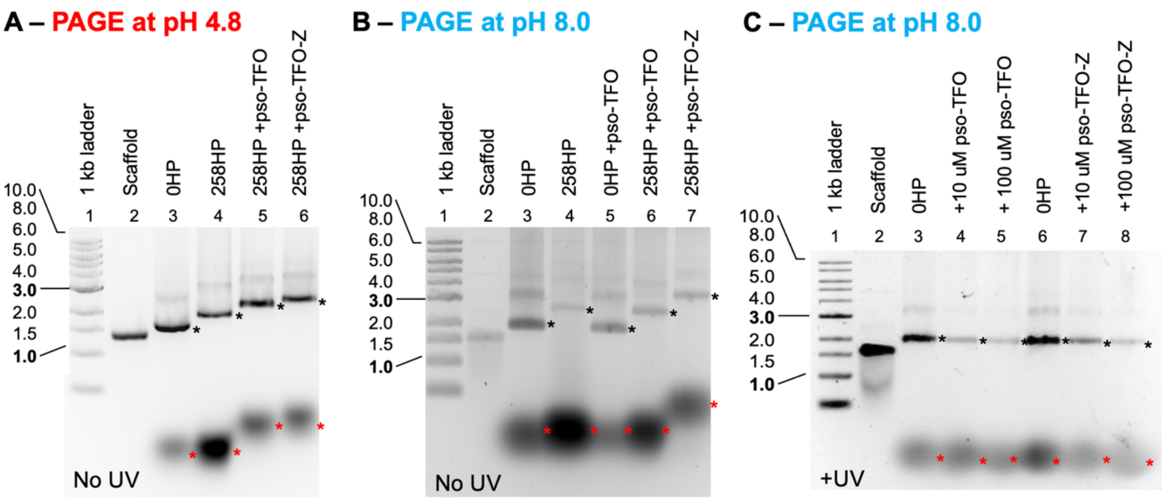

Fig S8. Targeting of origami hairpins by a TFO bearing a modified nucleobase analogue. AGE analysis of unpurified origami loaded with an unmodified (pso-TFO) and the Z-base-modified pso-TFO (pso-TFO-Z) bearing a 3′-biotin modification. Origamis were prepared in OB-8.0 by annealing 50 nM scaffold with 150 nM hairpinned staples (HP) in absence, or the presence of 100 uM pso-TFO. The samples in (C) were also subjected to irradiation at 365 nm for 60 seconds. Samples were then analysed by AGE at pH 4.8 (A) and pH 8.0 (B+C). The positions of the expected HP-staples and HP staple-TFO complexes are shown using a red asterisk, whilst the positions of the origami and origami-TFO complexes are shown using a black asterisk. The bands located above the origami band in each lane are due to misfolded aggregates (expected to form during origami foldingd[42)] and can be removed by ultracentrifugation during purification. The gels were post stained with GelRed and imaged using a GelDoc imager.

Commentary: Unlike the unmodified pso-TFO, the pso-TFO-Z containing the nucleobase analogue binds to the origami and remains stable during the electrophoresis experiment at pH 8.0 (B, lane 7). In contrast, both TFOs remain stable at pH 4.8 (A, lanes 5 and 6). As expected, no crosslinking was observed for either TFO (even after 60 seconds of UV exposure) when incubated with the 0HP origami structure that contains no TFO-binding hairpins and no oligopurine-oligopyrimidine target sites within the scaffold sequence (C, lanes 4,5 and 7,8). This corroborates the oligonucleotide experiments in Fig S5 that show a fully complementary target sequence is required for TFO binding and for crosslinking.

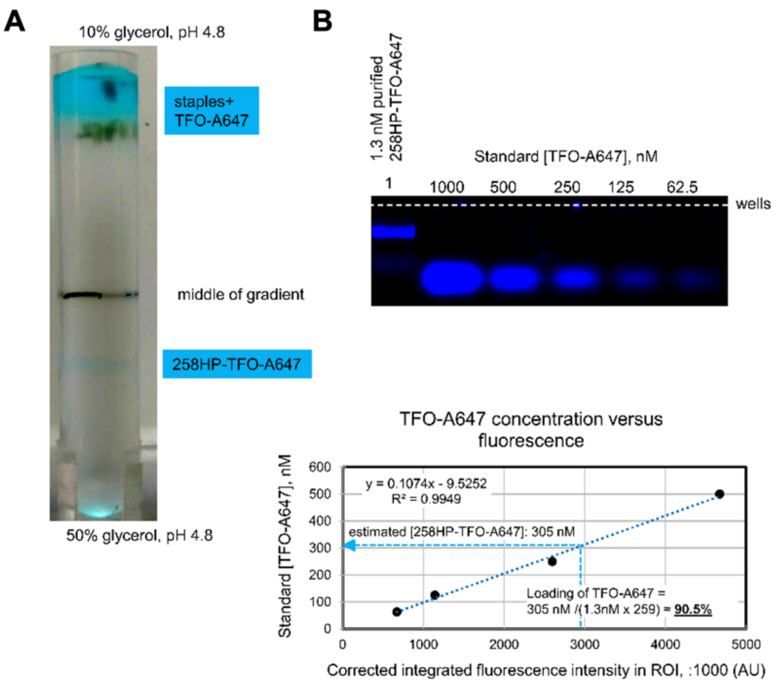

Figure S9. **Estimating loading of 258HP by TFO after purification by ultracentrifugation at pH 4.8.** 258HP origami was folded from 50 nM 8064 ssDNA scaffold and 150 nM HP-containing staples in OB-8.0 in the presence of 100 μM TFO labelled at the 3′ end with Alexa Fluor 647 (TFO-A647). **(A)** Migration of the 258HP-TFO-A647 complex in a 4 ml 10-50% glycerol gradient in OB-4.8, following a 2-hour ultracentrifugation. The 4-ml ultracentrifuge tube was photographed with a handheld smartphone camera. The band of the 258HP-TFO-A647 is visible to the naked eye in the bottom half of the gradient. **(B)** Estimation of Alexa 647-origami stoichiometry of 258HP-TFO-A647 complexes. The concentration of origami core DNA in the merged peak fractions was estimated spectrophotometrically (OD_{260}). The complex was run on a 1% AGE in a pH 4.8 running buffer alongside the same volumes of standard concentrations of free TFO-A647 oligonucleotide. Fluorescent bands were visualised by scanning for Cy5 fluorescence (top panel). Intensities of the standard bands were quantified and background-corrected, plotted against TFO-A647 concentration, and fit to a linear regression. The fit parameters and the Alexa 647 fluorescence intensity of the origami band were used to estimate the concentration of Alexa 647 in the purified origami sample (free of the unbound TFO-A647). The loading efficiency was estimated by dividing the Alexa 647 concentration in the origami sample by the theoretical Alexa 647 concentration expected at 100% loading (origami core concentration multiplied by 259, where the extra unit includes the fluorescence of the reporter staple Cy5).

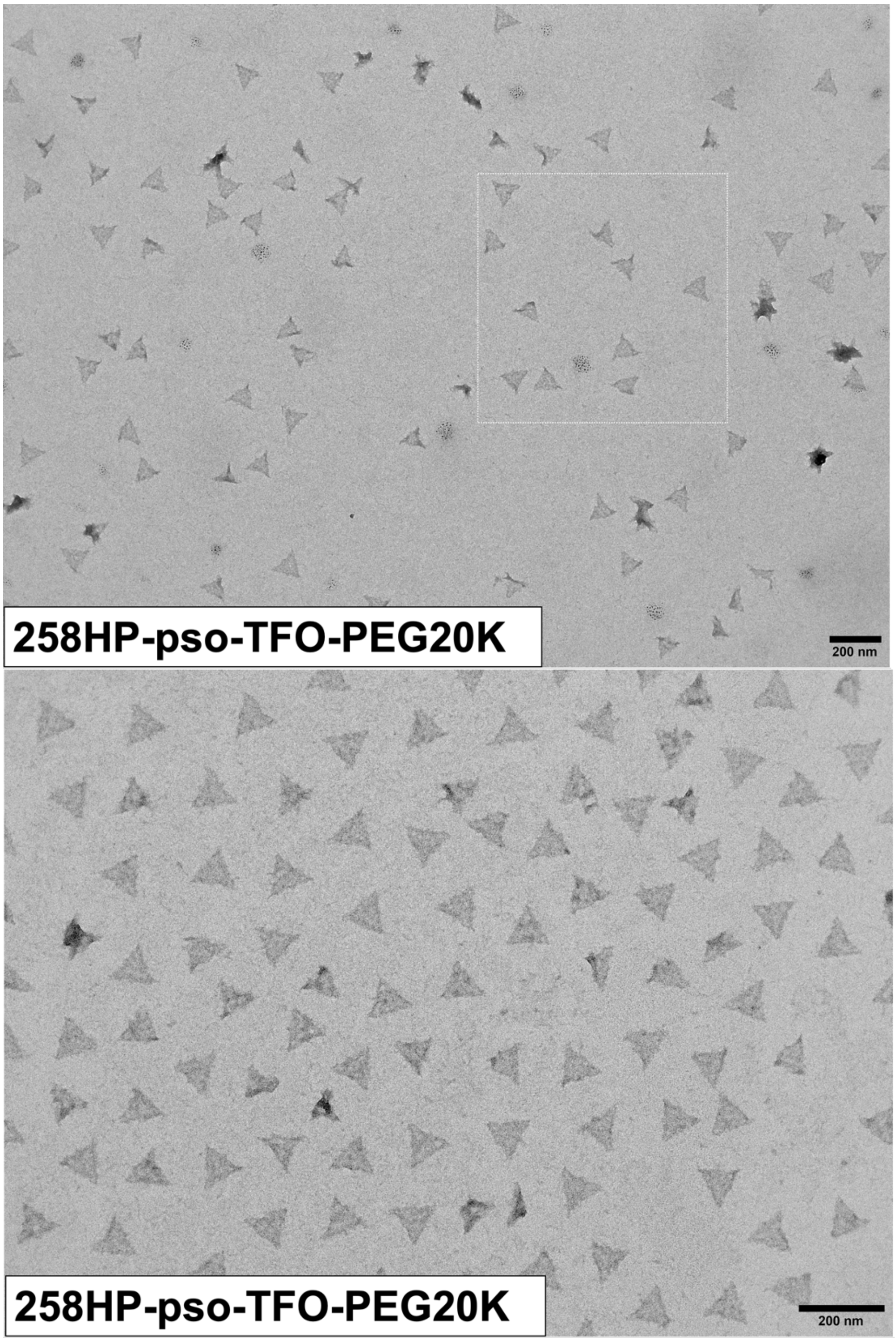

Figure S10. Representative TEM images of purified and crosslinked 258HP-pso-TFO-PEG20K. The dashed box indicates the cut-out region used in the main text.

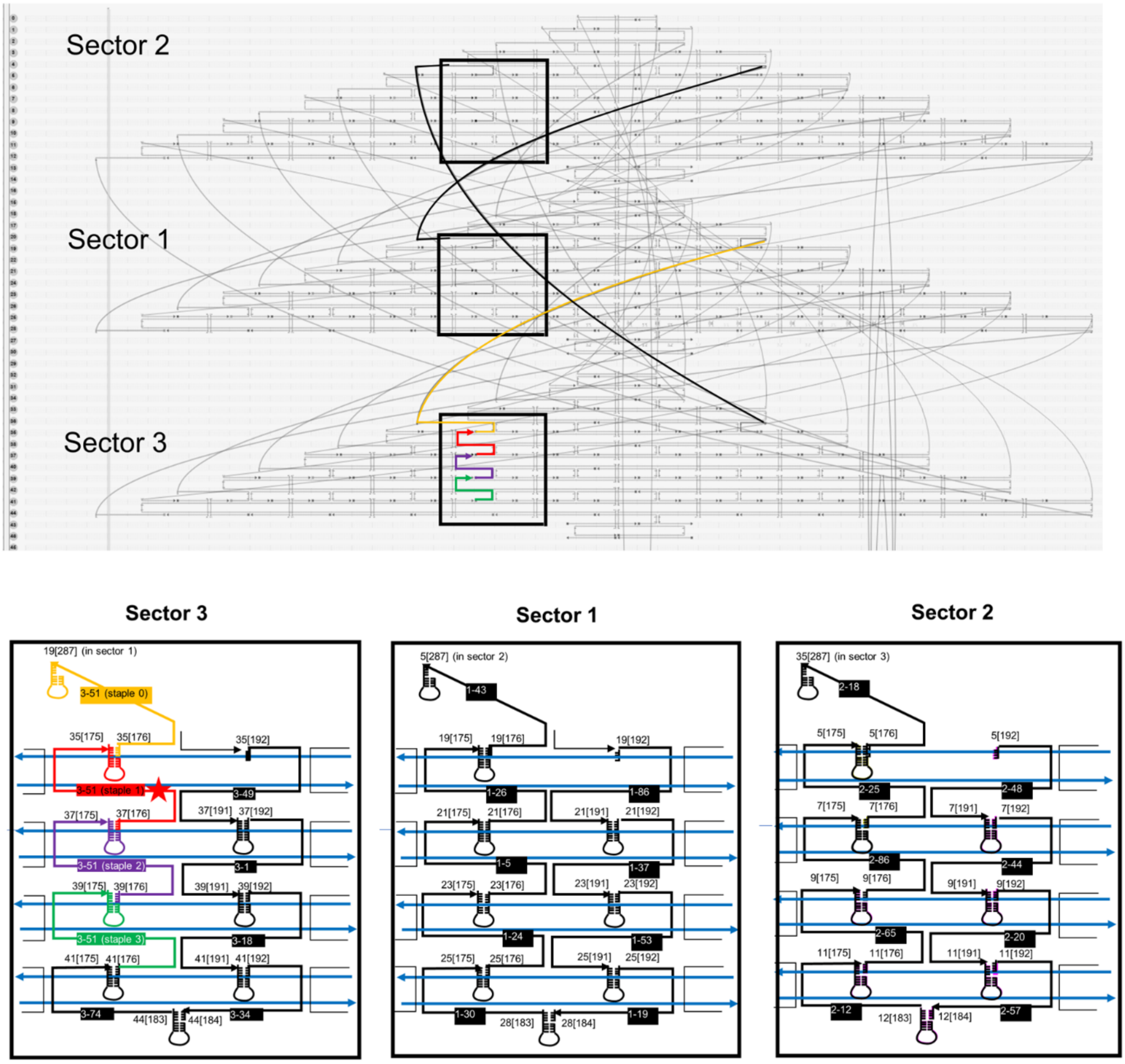

Figure S11. Routing and placement of adjacent HP staples in 1, 2, 3HP and 27HP origami. Staple routing for the hairpin-free triangular 0HP origami was displayed in CadNano 2 using the square lattice mode (top panel). The triangle is comprised of 45 helices in three sectors (15 helices per sector), with sectors linked together via poly(T)-containing linker staples. To design 1, 2, and 3 HP origamis, the coordinates of the 5′ and 3′ ends of the Cy5-labeled staple 1 (red, "start" and "end", respectively) were identified in Sector 3. Then, staple 0 was identified (orange) that starts next to the end of staple 1 and contains a polyT linker that extends into Sector 1 (long orange curve). Then, staple 2 was identified (purple, ends next to the start of staple 1), followed by staple 3 (green, ends next to the start of staple 2). CadNano coordinates of staple starts and ends are shown in the bottom panel as "helix[base-pair]". To design 27HP origamis, the process was continued as described for 1, 2, 3HP, starting at the start of staple 2 and working backwards to identify 6 additional sequential staples (black). In total, 9 staples originating in Sector 3 of 27HP (staples 0, 1, 2, 3 and the six additional ones) received hairpin spacer extensions. The process was then repeated for Sectors 1 and 2, using symmetrical coordinates of 9 staples in the respective helices. In 27HP, staple 0 had both the 5′ and the 3′ extensions, whereas in 1, 2, and 3 HP, staple 0 had only the 5′ 4-nt extension and no 3′ extension. See **Table 1** for all sequences and coordinates.

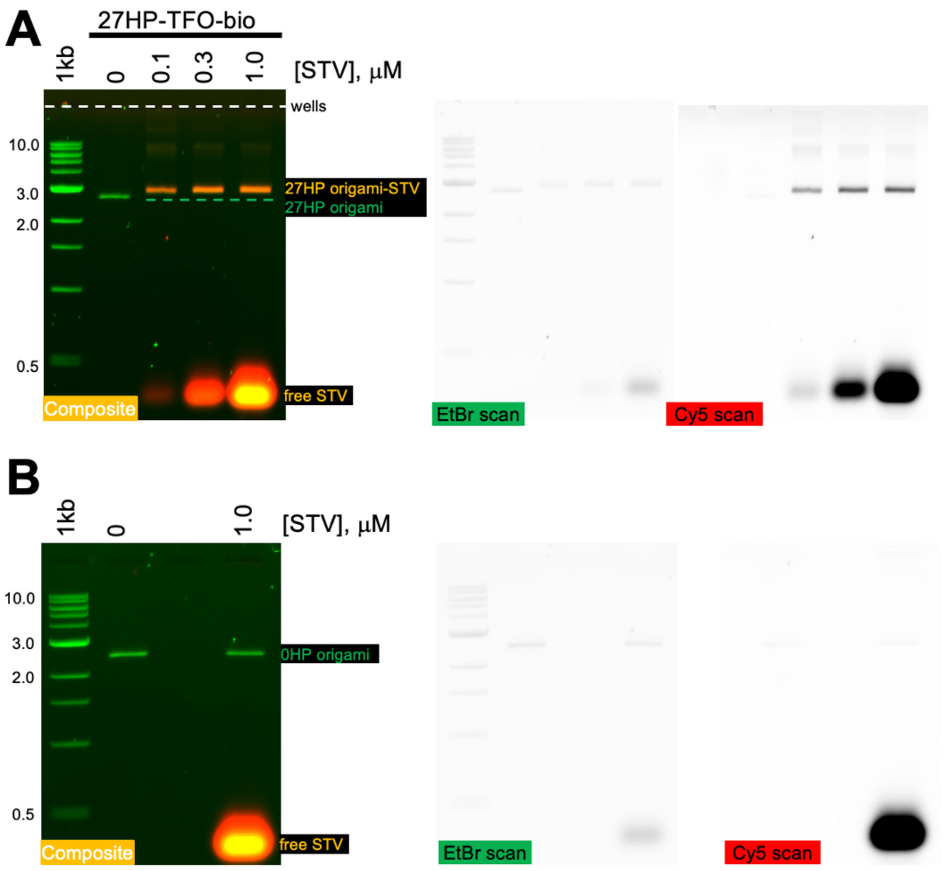

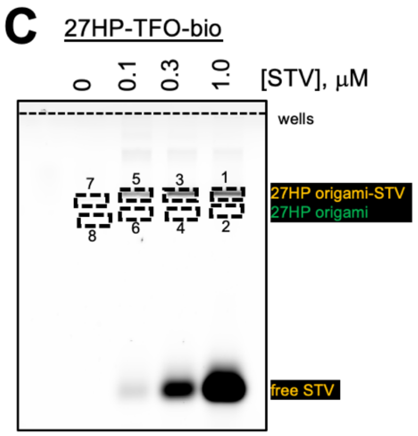

Region of Interest (ROI) ID	Raw integrated intensity of ROI in the 16-bit grayscale image of gel in A.	Streptavidin concentration, uM	Raw integrated density after background substraction	Ratio to backtround-substracted ROI 7, rounded to first digit
1	17284706	1	16809434	27.7
2	475272	background for ROI 1	0	
3	15735656	0.3	15304933	25.3
4	430723	background for ROI 3	0	
5	10324364	0.1	9890901	16.3
6	433463	background for ROI 5	0	
7	961251	0	606089	1
8	355162	background for ROI 7	0	

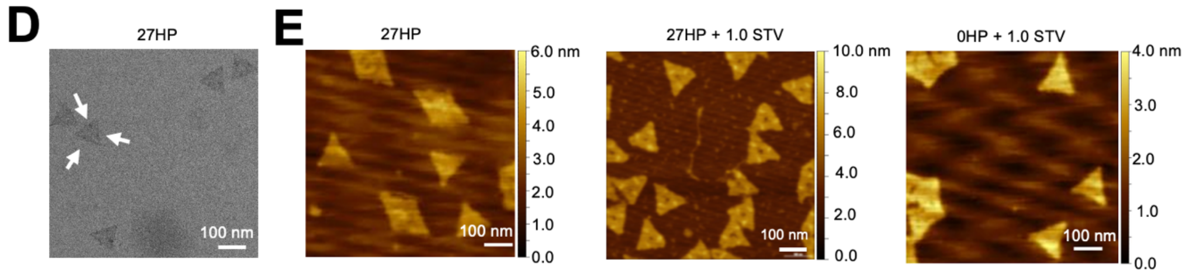

Figure S12. Site-specific loading of 27HP origami with pso-TFO-biotin and streptavidin. (A) 27HP-pso-TFO-biotin complexes were co-folded in OB-8.0 and crosslinked by irradiation for 10 seconds, purified by ultracentrifugation in OB-4.8, and incubated with recombinant

streptavidin tetramer (STV) at 0.1, 0.3 and 1 uM at pH 8.0. The complexes were then further incubated with an excess of oligonucleotide double-functionalized with biotin and Alexa Fluor 647 and run on a 1% AGE in a pH 8.0 running buffer, alongside dsDNA length markers. Electrophoresis continued until the unbound double-functionalized oligonucleotide ran out of the gel. Nucleic acid bands were visualized by scanning for ethidium bromide fluorescence (green colour map), and Alexa 647/Cy5-labeled bands were visualized by scanning for Cy5 fluorescence (red colour map); (B) Same as (A), but 0HP origami were folded and purified without pso-TFO-biotin, and only one streptavidin-loading reaction was performed at 1 uM STV, the highest STV concentration in (A); (C) Quantification of origami band intensities in gel in A (Cy5/Alexa 647 channel only) to estimate the STV loading in 27HP-pso-TFO-biotin complexes at different concentrations of STV. Regions of interest (ROIs) are shown with dashed lines. Odd-numbered ROIs contain origami bands, even-numbered ROIs are used for background subtraction. As a reference, the intensity of the origami band in the 0 [STV] lane (region of interest 7) was used: in this lane the only source of origami fluorescence is the single Cy5-labeled reporter staple 1 in origami. Origami fluorescence in lanes 0.1, 0.3, and 1.0 mM [STV] is the sum of the emission of biotin/Alexa 647 oligo bound to the STV-origami complex and the emission of the Cy5-labeled reporter staple 1. The ratio of intensity of origamis in the 0.1, 0.3, 1.0 [STV] lanes to the intensity in the 0 [STV] lane estimates the streptavidin loading (see Materials and Methods for details); (D) TEM imaging of 27HP origami highlighting the visibility of HPs; (E) AFM imaging of origamis targeted with STV. Co-fold assembly conditions were used to prepare 27HP origami loaded and crosslinked with pso-TFO-biotin as described in (A). In parallel, 0HP were folded and purified as in B. *Left*: AFM image of streptavidin-free 27HP-pso-TFO-biotin complexes (negative control). *Centre*: AFM image of 27HP-pso-TFO-biotin incubated with 1 uM streptavidin for 30 min. *Right*: AFM image of 0HP incubated with 1 uM streptavidin for 30 min (negative control). The central panel is identical to the AFM image in Figure 2E and is shown for side-by-side comparison with the negative controls.

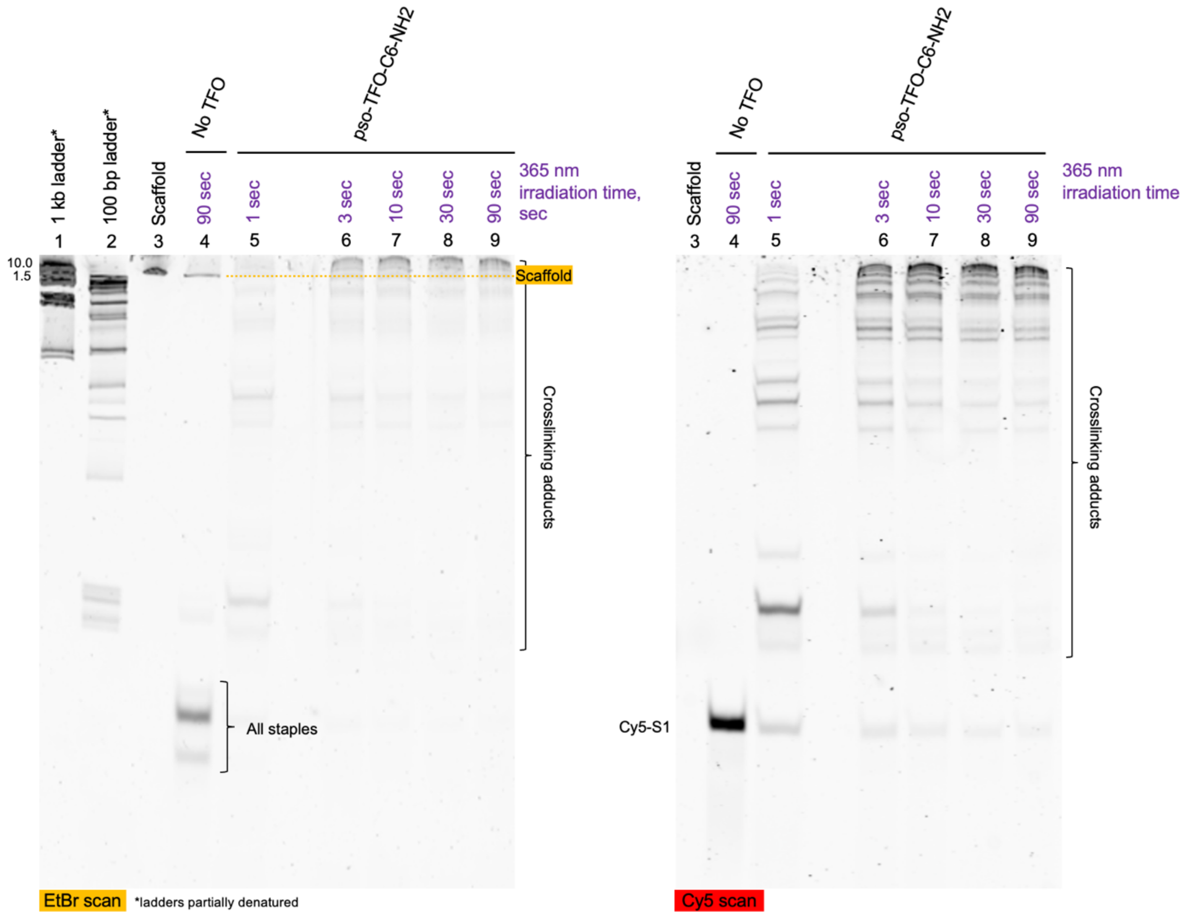

Figure S13 – Crosslinking of 258HP origami. Origami were co-folded in the presence of pso-TFO, purified by ultracentrifugation at pH 4.8, and then subjected to irradiation at 365 nm for the time points shown. The products of the reaction were denatured and then separated on an 8% denaturing PAGE gel. Bands were visualized by scanning for either EtBr (left gel) or Cy5-reporter staple 1 (right gel) fluorescence.

Commentary: Whilst it was not possible to assign the products of the crosslinking reaction due to the complexity of the system, it was evident that the crosslinking adducts (or "super-staples") are several hundred of nucleotides in length. In addition, the crosslinking reaction appears to be completely over in 10 seconds and longer UV exposure times did not lead to degradation, or non-specific cross-linking, of the scaffold strand (compare lane 3 and 4 in the EtBr gel on the left).

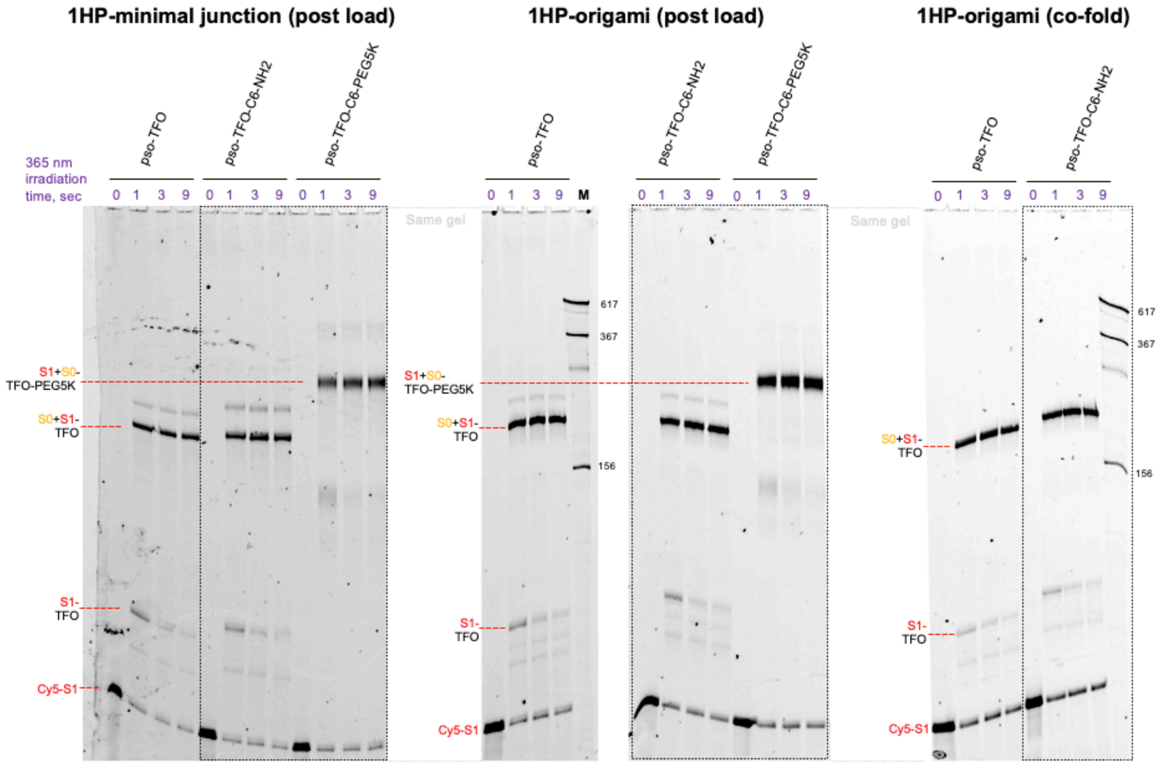

Figure S14 – Mechanism and specificity of pso-TFO-driven crosslinking. Experiments were undertaken on 1HP-origami and on 1HP-minimal junction as a control. Both contained one TFO-binding hairpin assembled using staple 0 (S0; orange) and the 3′-hairpin-modified staple 1 (S1; red). The 1HP minimal junction used a short minimal "pseudo-scaffold" oligo which simulated the origami scaffold. The complexes were either post loaded or co-folded with the pso-TFOs (pso-TFO, pso-TFO-C6-NH_2, or pso-TFO-C6-PEG5K) as indicated. Samples were subjected to irradiation at 365 nm for the time points shown, and the products of the reaction were denatured and then separated by 8% denaturing PAGE. Bands were visualized by scanning for Cy5-labeled staple 1 fluorescence. Lane M contains linear Cy5-end-labeled PCR products of the indicated lengths.

Commentary: For each TFO the same mono (S1-TFO) and bis-adducts (S+S1-TFO) are evident regardless of either the construct (the origami free 1HP-minimal junction or 1HP-origami) or the method of assembly (co-fold with the TFOs or post load with the TFOs). The dashed boxes indicate the cut-out regions used in the main text and due to the markers can be directly compared between gels and the reactions shown in Fig S15.

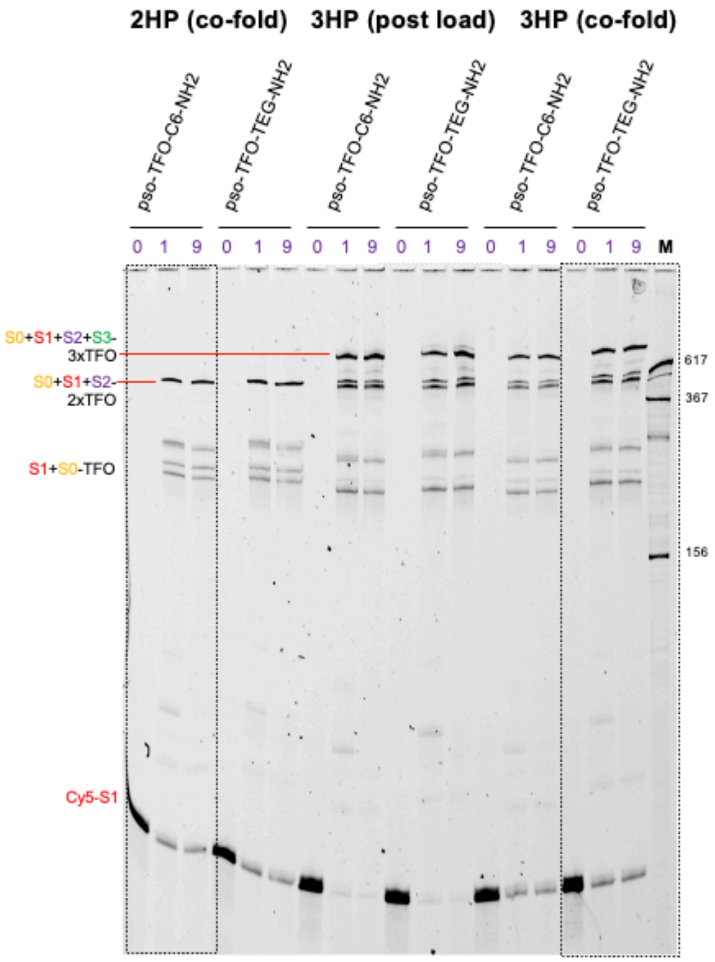

Figure S15 – Mechanism and specificity of pso-TFO-driven crosslinking. Experiments were undertaken on 2HP and 3-HP origami that contained two or three TFO binding hairpins, respectively. HP2 was assembled using 3′-hairpin-modified staples 0, 1, and 2, and HP3 – using 3′-hairpin-modified staples 0, 1, 2, and 3. Staples 0, 1, 2, and 3 are shown as orange, red, purple and green labels, respectively. The complexes were either post loaded or co-folded with the pso-TFOs (pso-TFO-C6-NH_2 or pso-TFO-TEG-NH_2) as indicated. Samples were subjected to irradiation at 365 nm for the time points shown, and the products of the reaction were denatured and then separated by 8% denaturing PAGE. Bands were visualized by scanning for Cy5-labeled staple 1 fluorescence. Lane M contains linear Cy5-end-labeled PCR products of indicated lengths.

Commentary: For each TFO the same adducts are evident for each origami structure, regardless of the method of assembly (co-fold or post load). The assignment of intermediate adduct is complicated by incomplete mono- and bis-adduct formation for each of the 1, 2 and 3HP structures. The slowest migrating species for each of the 2 (S0+S1-TFO), 3 (S0+S1+S2-2xTFO) and 4 staple complexes (S0+S1+S2+S3-2xTFO) are shown. Fig S16 highlights the potential adducts/products that can be formed for 1, 2, 3HP structures due to incomplete crosslinking. The dashed boxes indicate the cut-out regions used in the main text and due to the markers can be directly compared to the reactions shown in Figure S14.

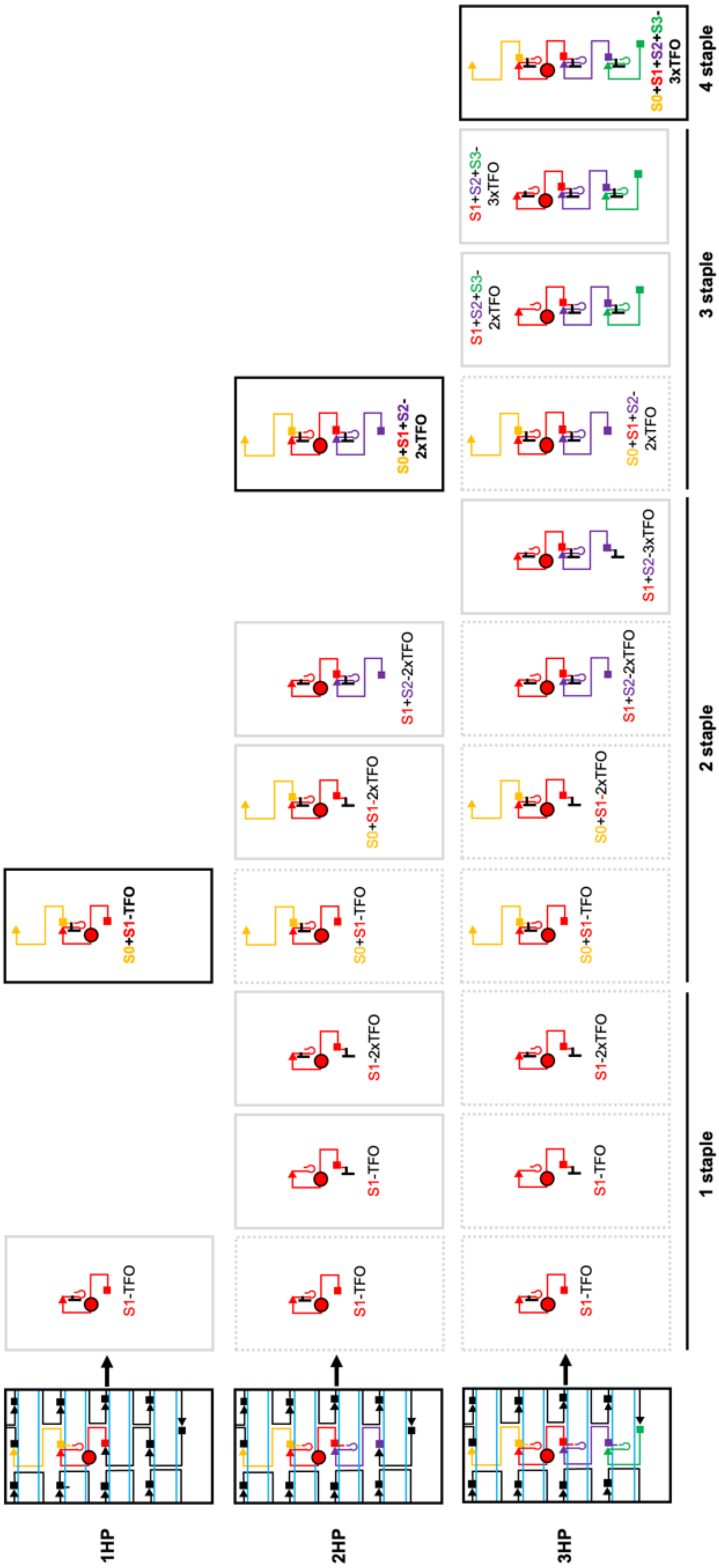

Figure S16 – Complexity of adduct formation with 1, 2, and 3HP origami due to incomplete cross-linking. Boxed adducts are the intended products of the reaction.

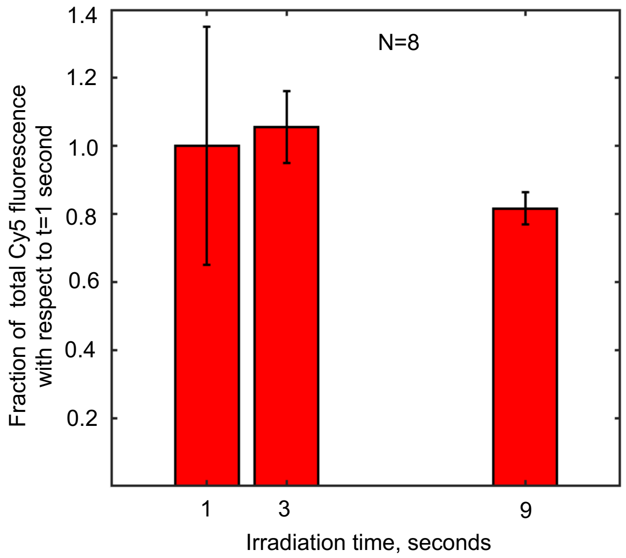

Figure S17. **Photobleaching of Cy5-labeled 1HP structures in conditions inducing efficient pso-TFO crosslinking.** 1HP origami and 1 HP minimal scaffold were loaded with pso-TFO, irradiated for 1, 3, and 9 seconds and analysed with 8% denaturing PAGE. The gel was scanned for Cy5 fluorescence, and intensities of all detectable bands in lanes corresponding to the 1-, 3-, and 9-second samples were measured in Image J and corrected by background subtraction. Sum of intensities of all bands in each lane was calculated and normalized for the sum of intensities of bands in the lane with the 1-second sample (to account for variation in the volumes of samples loaded). Averages of eight irradiation experiments are shown, with the error bars corresponding to the standard deviations.

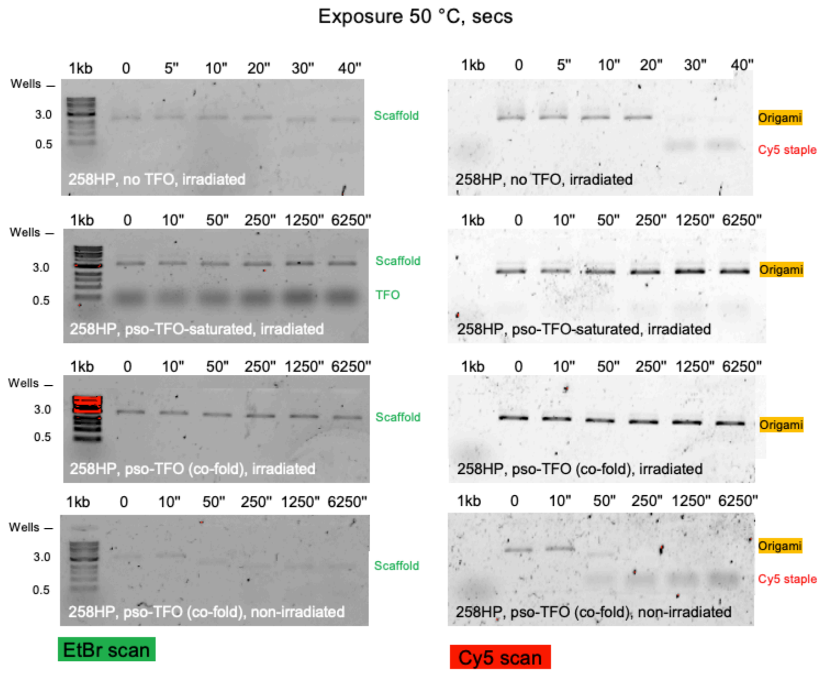

Figure S18. **Heat challenge of 258HP origami subjected to pso-TFO driven crosslinking (EtBr and Cy5 scans).** Experiments were undertaken on origami folded in the absence (top panel) or presence of pso-TFO-PEG$_4$-NH$_2$ under TFO-saturating (upper middle panel) or co-folding (lower middle and bottom panel) conditions. Origami containing pso-TFOs were subjected to irradiation at 365 nm for 10 seconds. Samples of 2 nM folded origami were then subjected to heat challenge at 50 °C for the timepoints indicated. Samples were run on a 1% AGE in a pH 4.8 running buffer at room temperature. Bands for the origami and “super-staples” were visualised by scanning for Cy5-reporter staple fluorescence, whilst the scaffold strand, free TFO and dsDNA markers were visualised by scanning for ethidium bromide fluorescence.

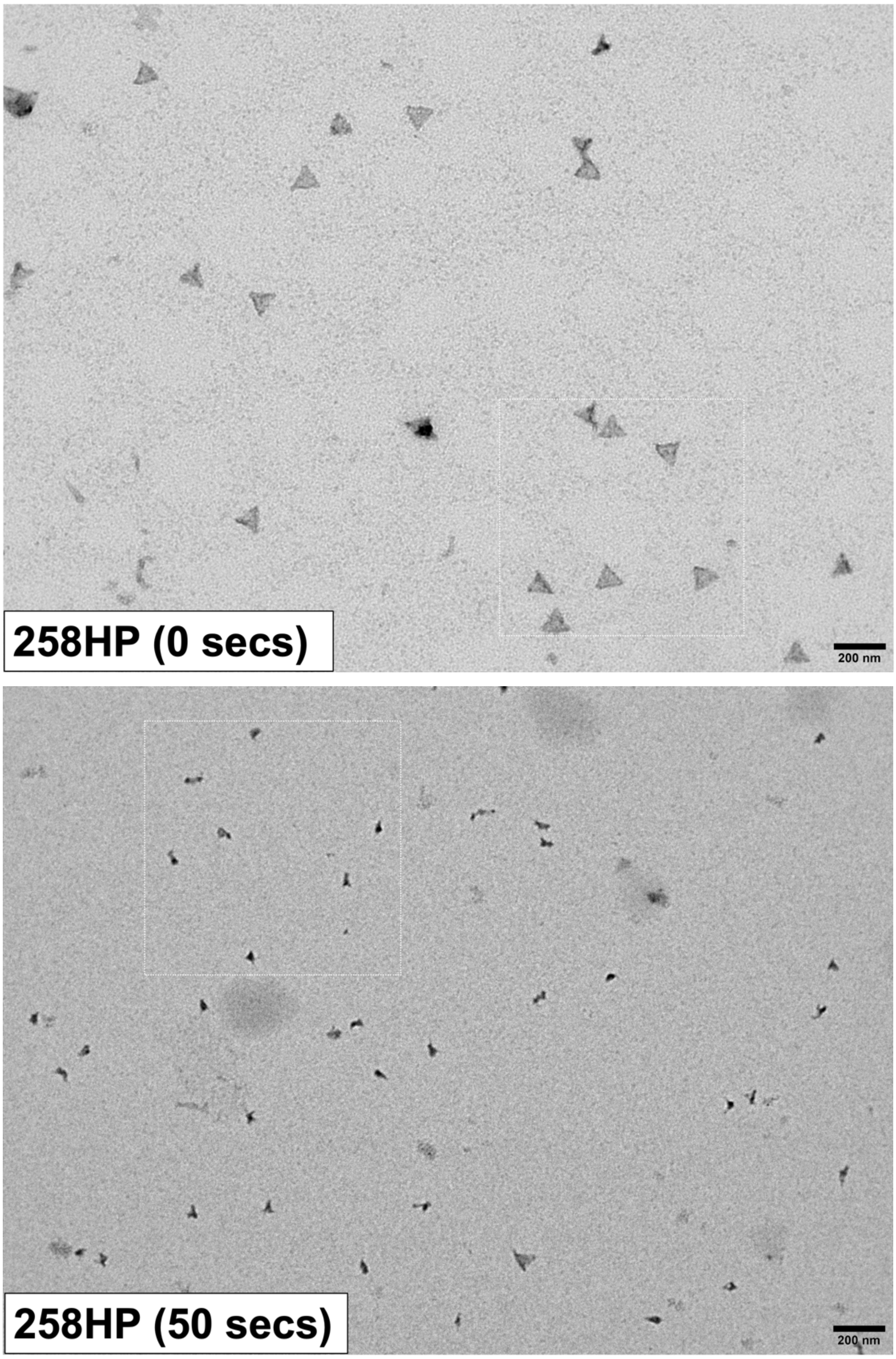

Figure S19 - Representative TEM images of purified 258HP origami triangles after heat treatment at 50 °C for 0 (top image) and 50 (bottom image) seconds. The dashed box indicates the cut-out region used in the main text.

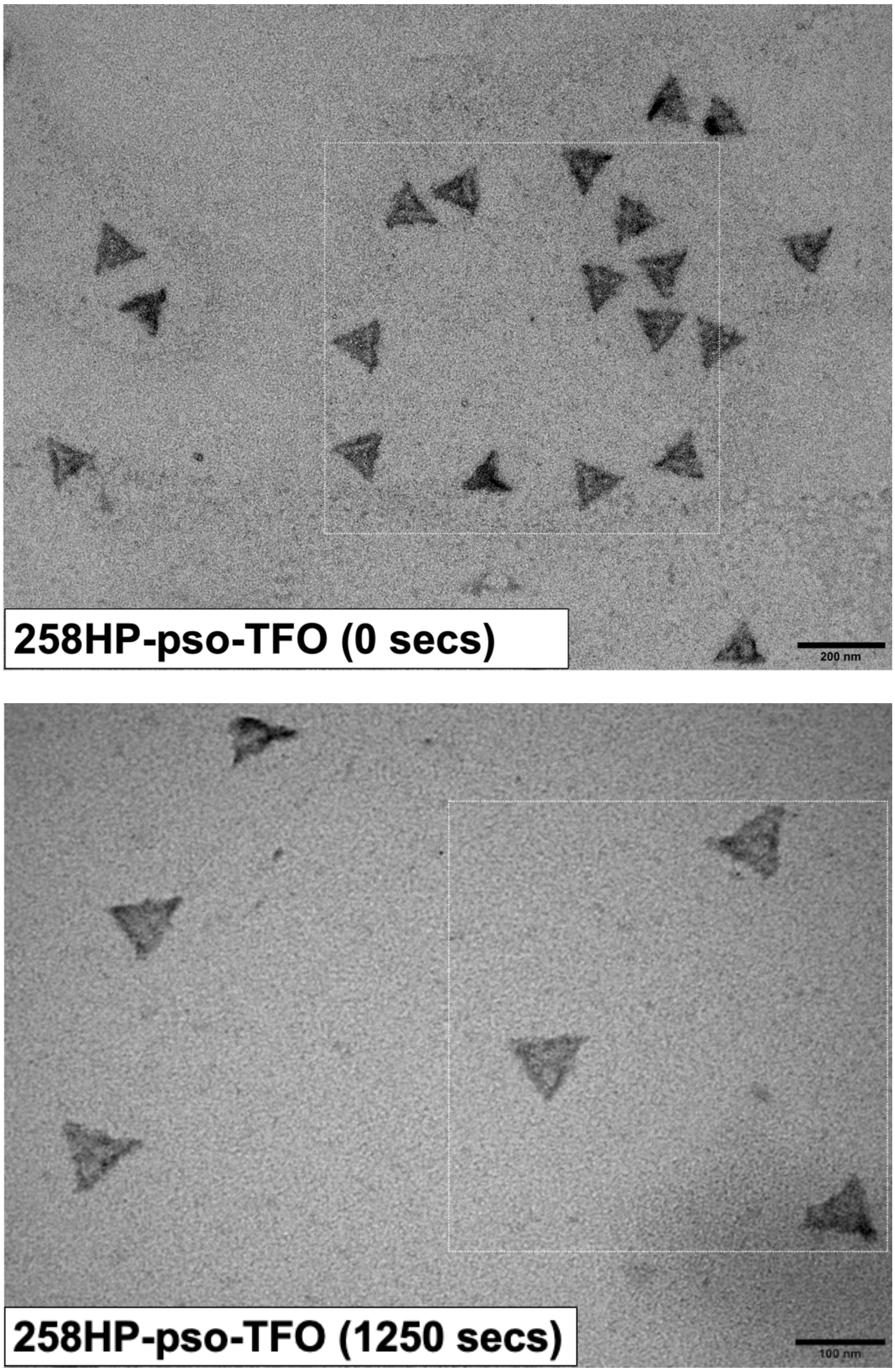

Figure S20 - Representative TEM images of purified and crosslinked 258HP-pso-TFO origami triangles after heat treatment at 50 °C for 0 (top image) and 50 (bottom image) seconds. The dashed box indicates the cut-out region used in the main text.

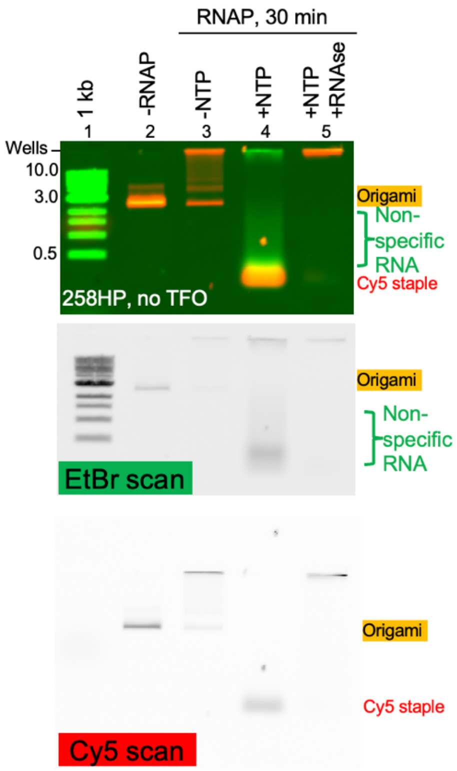

Figure S21. RNAP challenge of 258HP origami (Cy5 and EtBr scans). Experiments were undertaken on origami folded in the absence of a pso-TFO. Samples of 2-5 nM folded origami were then incubated with 2.5 µM T7 RNAP in the presence or absence of 1 mM NTPs at 37 °C for 30 minutes and then subjected to proteinase K treatment for 15 minutes to degrade the protein. Samples were run on a 1% AGE in a pH 4.8 running buffer at room temperature. Bands for the origami and the Cy5-labelled staple 1 were visualised by scanning for Cy5 fluorescence, whilst the scaffold, RNA transcripts and dsDNA markers were visualised by scanning for ethidium bromide fluorescence.

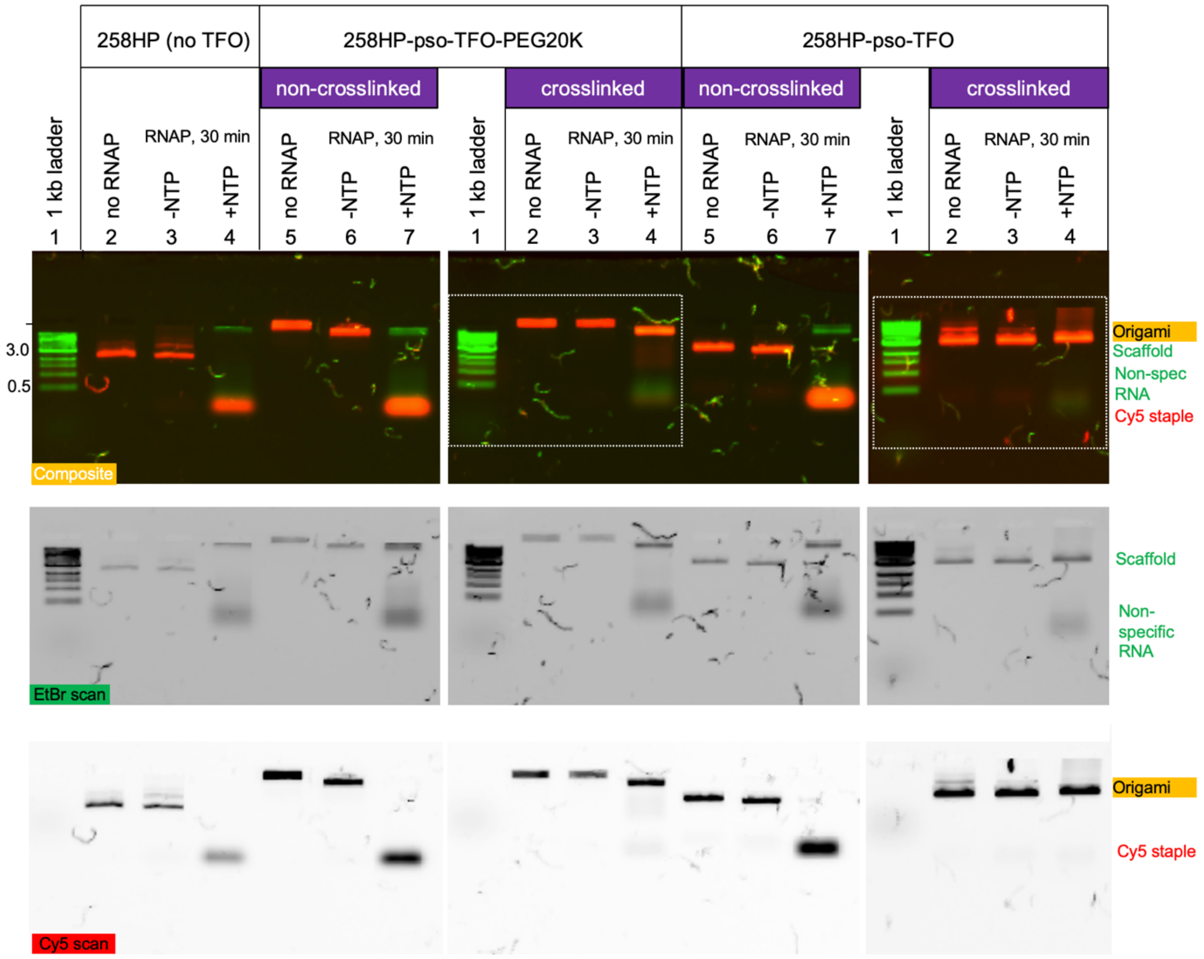

Figure S22. RNAP challenge of 258HP origami subjected to pso-TFO-driven crosslinking (Cy5 and EtBr scans). Experiments were undertaken on origami folded in the absence or presence of a pso-TFO or its PEG20K 3′-conjugate. These were prepared under co-fold annealing conditions, and the origami containing pso-TFOs were subjected to irradiation at 365 nm for 10 seconds. Samples of 2-5 nM folded origami were then incubated with 2.5 μM T7 RNAP in the presence or absence of 1 mM NTPs at 37 °C for 30 minutes and then subjected to proteinase K treatment for 15 minutes to degrade the protein. Samples were run on a 1% AGE in a pH 4.8 running buffer at room temperature. Bands for the origami and the Cy5-labelled staple 1 were visualised by scanning for Cy5 fluorescence, whilst the scaffold, RNA transcripts and dsDNA markers were visualised by scanning for ethidium bromide fluorescence.

Commentary: Complexes in the absence or presence of TFO that were not crosslinked were disassembled by RNAP and the released scaffold strand remained high up in the gel during electrophoresis (presumably due to non-specific binding). In contrast, TFO-258HP complexes that were crosslinked remained protected from disassembly by RNAP. The dashed box indicates the cut-out region used in the main text.

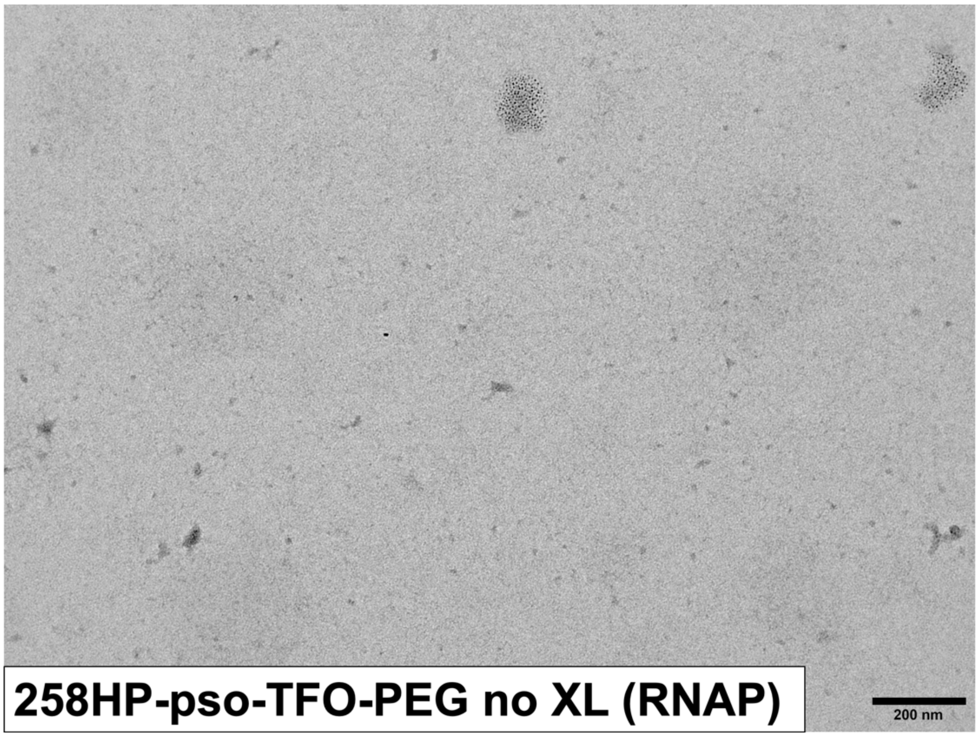

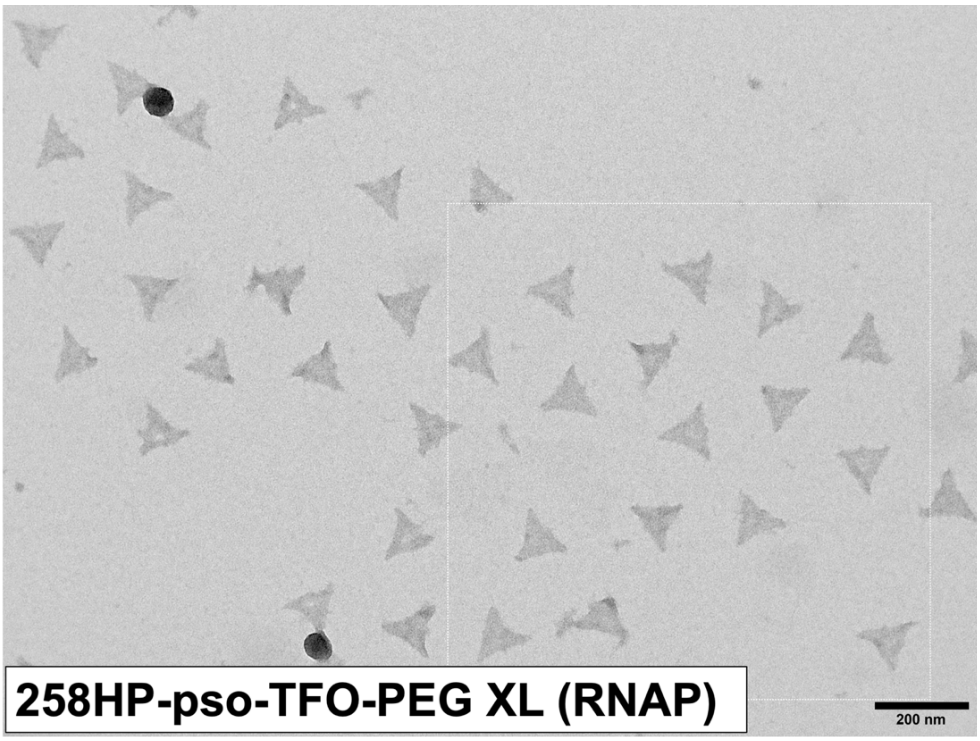

Figure S23 - Representative TEM images of non-irradiated (top) and irradiated (bottom) 258HP-pso-TFO-PEG20K origami that had been subjected to RNAP challenge in the presence of NTPs. The dashed box indicates the cut-out region used in the main text.

Supplementary References

(1) Douglas, S. M.; Marblestone, A. H.; Teerapittayanon, S.; Vazquez, A.; Church, G. M.; Shih, W. M. Rapid Prototyping of 3D DNA-Origami Shapes with CaDNAno. *Nucleic Acids Res.* **2009**, *37* (15), 5001–5006. https://doi.org/10.1093/nar/gkp436.

(2) Rajendran, A.; Endo, M.; Katsuda, Y.; Hidaka, K.; Sugiyama, H. Photo-Cross-Linking-Assisted Thermal Stability of DNA Origami Structures and Its Application for Higher-Temperature Self-Assembly. *J. Am. Chem. Soc.* **2011**, *133* (37), 14488–14491. https://doi.org/10.1021/ja204546h.

(3) TBE Buffer. *Cold Spring Harb. Protoc.* **2006**, *2006* (1), pdb.rec8458. https://doi.org/10.1101/pdb.rec8458.

(4) Zhang, Z.; Revyakin, A.; Grimm, J. B.; Lavis, L. D.; Tjian, R. Single-Molecule Tracking of the Transcription Cycle by Sub-Second RNA Detection. *eLife* **2014**, *3*, e01775. https://doi.org/10.7554/eLife.01775.

(5) Faas, F. G. A.; Rieger, B.; Van Vliet, L. J.; Cherny, D. I. DNA Deformations near Charged Surfaces: Electron and Atomic Force Microscopy Views. *Biophys. J.* **2009**, *97* (4), 1148–1157. https://doi.org/10.1016/j.bpj.2009.06.015.

(6) Cherny, D. I.; Jovin, T. M. Electron and Scanning Force Microscopy Studies of Alterations in Supercoiled DNA Tertiary Structure. *J. Mol. Biol.* **2001**, *313* (2), 295–307. https://doi.org/10.1006/jmbi.2001.5031.

(7) Lin, C.; Perrault, S. D.; Kwak, M.; Graf, F.; Shih, W. M. Purification of DNA-Origami Nanostructures by Rate-Zonal Centrifugation. *Nucleic Acids Res.* **2013**, *41* (2), e40–e40. https://doi.org/10.1093/nar/gks1070.
